# Techno-economic assessment of different small-scale electrochemical NH_3_ production plants†

**DOI:** 10.1039/d4ee03299c

**Published:** 2024-10-03

**Authors:** Boaz Izelaar, Mahinder Ramdin, Alexander Vlierboom, Mar Pérez-Fortes, Deanne van der Slikke, Asvin Sajeev Kumar, Wiebren de Jong, Fokko M. Mulder, Ruud Kortlever

**Affiliations:** a Process and Energy Department, Faculty of Mechanical Engineering, Delft University of Technology 2628 CB Delft The Netherlands R.Kortlever@tudelft.nl; b Engineering, Systems and Services Department, Faculty of Technology, Policy and Management, Delft University of Technology 2628 BX Delft The Netherlands; c Chemical Engineering Department, Faculty of Applied Sciences, Delft University of Technology 2629 HZ Delft The Netherlands

## Abstract

Electrochemical ammonia synthesis *via* the nitrogen reduction reaction (NRR) has been poised as one of the promising technologies for the sustainable production of green ammonia. In this work, we developed extensive process models of fully integrated electrochemical NH_3_ production plants at small scale (91 tonnes per day), including their techno-economic assessments, for (Li-)mediated, direct and indirect NRR pathways at ambient and elevated temperatures, which were compared with electrified and steam-methane reforming (SMR) Haber–Bosch processes. The levelized cost of ammonia (LCOA) of aqueous NRR at ambient conditions only becomes comparable with SMR Haber–Bosch at very optimistic electrolyzer performance parameters (FE > 80% at *j* ≥ 0.3 A cm^−2^) and electricity prices (<$0.024 per kW h). Both high temperature NRR and Li-mediated NRR are not economically comparable within the tested variable ranges. High temperature NRR is very capital intensive due the requirement of a heat exchanger network, more auxiliary equipment and an additional water electrolyzer (considering the indirect route). For Li-mediated NRR, the high lithium plating potentials, ohmic losses and the requirement for H_2_, limits its commercial competitiveness with SMR Haber–Bosch. This incentivises the search for materials beyond lithium.

## Abstract 2

Electrochemical ammonia synthesis holds promise for sustainable ammonia production. Here, we compare process models and techno-economic assessments for small scale electrochemical NH__3__ production plants to electrified and SMR Haber–Bosch processes.

## Graphical abstract

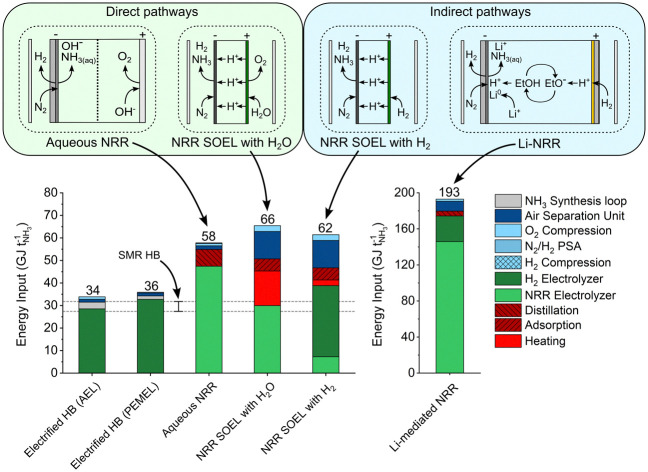

**Broader context**

Electrochemical ammonia synthesis based on the nitrogen reduction reaction (NRR), wherein nitrogen gas and a proton source are electrochemically reduced with renewable electricity, holds the promise of enabling the production of carbon-free ammonia. The NRR can occur at ambient conditions, elevated temperatures in a solid oxide cell and *via* lithium as an active mediator the proton source can originate “direct” from water or “indirect” *via* hydrogen gas. A large majority of the current research in the field focusses on improving the electrolyzer performance instead of exploring the overall techno-economic feasibility of the mentioned NRR pathways. Here, we have developed comprehensive conceptual process models of direct and indirect NRR pathways at ambient and elevated temperatures, Li-mediated NRR and the electrified Haber–Bosch process as a sustainable benchmark. This gives key insights into the required electrolyzer performance metrics to reach economic parity with SMR Haber–Bosch. Overall, we find that the inherently low energy efficiencies of the electrolysis steps (NRR or water electrolyzers) are limiting the economic competitiveness of electrochemical ammonia synthesis.

## Introduction

Ammonia (NH_3_) ranks among the largest produced synthetic chemicals in the world with an annual market size of ∼180 Mt, total market capitalization of around $76 billion USD and an expected annual growth of 3–5%.^1,2^ The majority of NH_3_ (80%) is processed into N-based fertilizers such as urea and ammonium nitrate, where the latter is also used for the production of explosives (5%). Other applications are in the manufacturing of cleaning detergents, pharmaceuticals, rubber and other polymers (15%).^3,4^ The vast majority of NH_3_ is produced by the conventional thermochemical Haber–Bosch process, where high temperatures (300–500 °C) and pressures (200–300 atm) are required to reach sufficient NH_3_ conversions from N_2_ and H_2_ over an iron catalyst.^5^ Due to these intensive process conditions, this process requires substantial capital investments, with costs reaching billions of USD for plants producing >2000 tonnes ammonia per day to minimize costs by economy of scale.^6^ The downside of these centralized plants are the increasing transportation costs, especially to remote areas. However, small scale plants (typically <100 tonnes per day) catering to local markets with regional price agreements have been reported.^7^

The most energy efficient method for H_2_ feed production is steam methane reforming (SMR) based on natural gas, but this has significant environmental consequences as it releases 1.22 tCO_2_ per tNH_3_ alongside additional emissions related to burning fuel, natural gas extraction and other losses.^8^ Approximately 1.2% of the anthropogenic CO_2_ emissions are caused by the NH_3_ sector, necessitating a transition to greener production alternatives to meet the net-zero emissions goal in 2050.^9^ A significant reduction in emissions can be accomplished if the SMR or coal gasification plant is substituted by greener alternatives, such as water electrolysis. This “electrified” version of the Haber–Bosch process, first implemented in 1928 (Rjukan, Norway), was discontinued in the 1960's when SMR became more competitive because of the cheap availability of natural gas; however, it is now poised for a comeback.^10^ This is mainly due to the decreasing costs for renewable electricity from onshore wind and solar photovoltaics.^11^ Moreover, the expected decline in manufacturing costs of alkaline and proton-exchange membrane electrolyzers (decreasing 3.0% and 4.8% each year)^12^ for water electrolysis further enhances the competitiveness for the electrified Haber–Bosch in the near future.^8,13,14^

Alternative technologies for sustainable NH_3_ production are based on the electrochemical nitrogen reduction reaction (NRR), where nitrogen gas in combination with a proton source can in theory be electrochemically reduced with electricity from renewable energy sources. The proton source can be “direct” from water or “indirect” from hydrogen produced by water electrolysis. Both the direct and indirect NRR electrolyzer can in theory operate at ambient temperatures and pressures, thereby saving energy and capital expenditure on compressors and heat exchangers. Another promising approach is NRR at elevated temperatures, harvesting waste heat from the chemical industry to produce NH_3_ at higher rates and energy efficiencies.

Most of the current research in electrochemical NRR emphasizes the development of active, selective and stable electrocatalysts for the electrolyzers. Only a handful of studies have assessed the techno-economic feasibility of NRR technologies on a system level,^15^ and focus mostly on the electrolyzer costs.^16,17^ Particularly, there is a lack of knowledge about the future design, energy consumption and techno-economic feasibility of a fully integrated electrochemical NH_3_ process plant, including upstream and downstream separation units, heat integration and storage. To that end, we have developed comprehensive conceptual process models of direct and indirect NRR pathways at ambient and elevated temperatures, Li-mediated NRR and the electrified Haber–Bosch process as a sustainable benchmark. Moreover, we have used a consistent set of assumptions to perform a comparative analysis between these technologies, which gives key insights into the required electrolyzer performance metrics and the minimum ammonia production price necessary to enable carbon emission-free ammonia.

## Process design assumptions and descriptions

It is expected that electrochemical ammonia plants operate in a decentralized manner.^15,18^ Therefore, a small capacity of 91 tNH_3_ per day is considered, which is based on the smallest commercial SMR HB plant that supplies only to local markets.^7^ The synthesis process is assumed to be continuous, which means that a variable availability of renewable energy is outside the scope of the current study and capacity factors of the process are high.

The majority of the mass balance and economic calculations were performed in conventional spreadsheet software. Aspen Plus^TM^ was used to model distinct unit operations, such as distillation and adsorption columns, flash evaporation, pump and compressor duties and heat integration if necessary. All NRR electrolyzers are considered as stoichiometric black box models. The total cell voltage (*E*_cell_) is defined as:

1 *E*_cell_ = *E*_eq_ + *η*_cat_ + *η*_an_ + *η*_mem_ + *η*_ohmic_,

which summates the equilibrium potential (*E*_eq_), cathodic (*η*_cat_) and anodic (*η*_an_) half-reaction overpotentials, ionic transport resistance in the membrane (*η*_mem_) and electrolyte (*η*_ohmic_). We used the Nernst equation to calculate the *E*_eq_ from the standard equilibrium potential (*E*_0_). The activation overpotentials (*η*_cat_ and *η*_an_) were estimated by using approximations of the Butler–Volmer equation. In case the exchange current density (*j*_0_) is relatively small with respect to the applied current density (*j*/*j*_0_ > 4), which is often the case for electrocatalytic processes at ambient conditions, the activation overpotential can be calculated with the Tafel equation:2
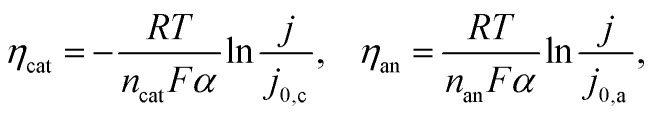
where *n*_cat_ and *n*_an_ are the number of electrons transferred per mole of product by the reduction and oxidation reactions (3 for the nitrogen reduction reaction, 4 for the oxygen evolution reaction, 2 for the hydrogen oxidation reaction), *F* is the Faraday constant (*F* = 96 485 C mol^−1^) and *α* the transfer coefficient (*α* = 0.5). Electrocatalytic reactions occurring at higher temperatures have typically an *j*_0_ larger than *j*, meaning that the hyperbolic sine approximation stated in eqn (3) is more appropriate.^19^3
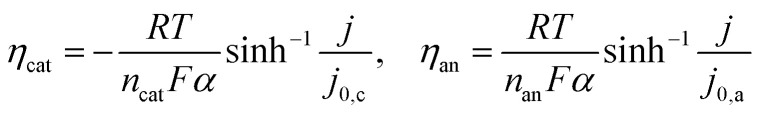
Due to the lack of reliable kinetic data on the NRR in aqueous media, we estimated the *j*_0_*via* the Tafel equation and used 0.4 V as the minimum required *η*_NRR_ at *j* = 0.1 A cm^−2^.^5,20^ For the NRR at high temperature (550 °C), the *j*_0_ of the hydrogen evolution reaction was assumed and taken as 0.4 A cm^−2^.^21^ Expressions for the ohmic losses and more assumptions related to the electrochemical models are provided in the “Supplemental Methods” in the ESI.†

The power consumption in watt of the electrolyzer (*P*_elect_) is a function of *E*_cell_ and the total current in amperes (*I*):4
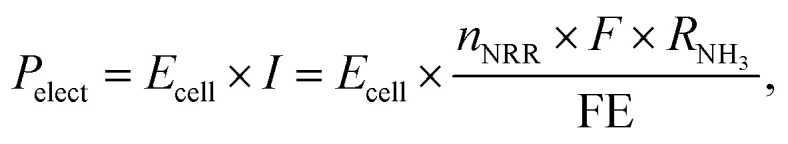
wherein the latter can be expressed in the NH_3_ mole-based production capacity per seconds (*R*_NH_3__), and the faradaic efficiency (FE) defined as the ratio between the charge consumed by the NRR and the total charge, to include the losses from the hydrogen evolution reaction (HER). Losses associated with power electronics, connections and cables are excluded.

**Fig. 1:**
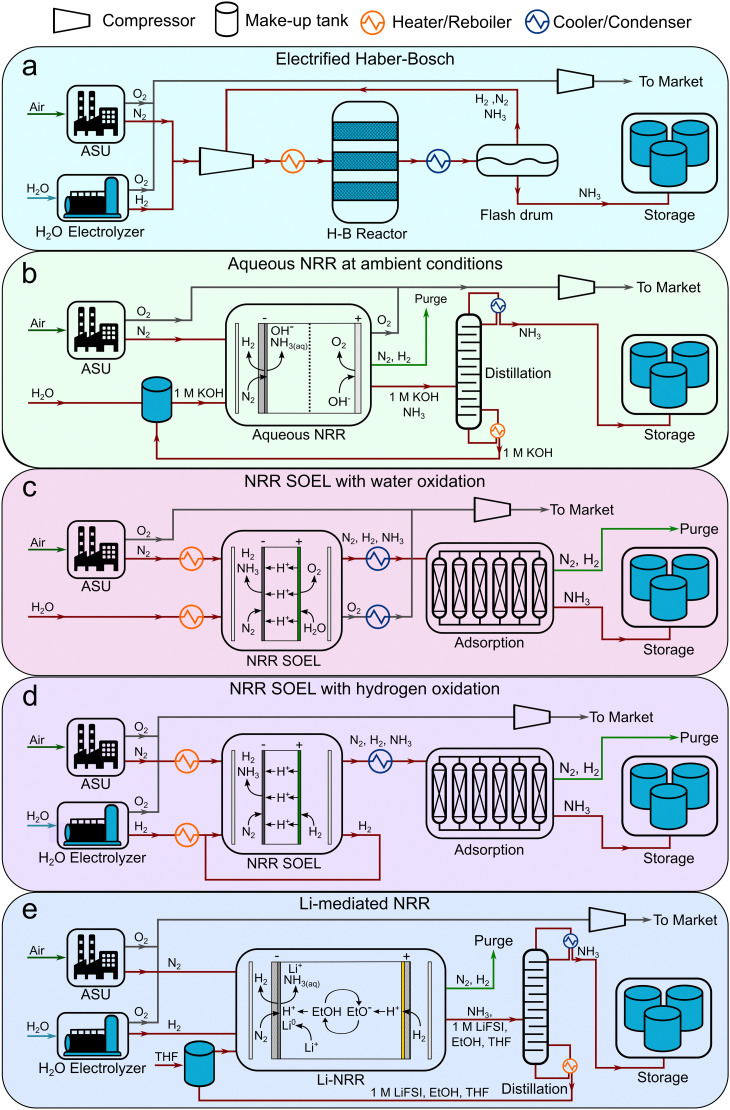
Schematic process diagrams of (a) electrified Haber–Bosch, (b) aqueous NRR at ambient conditions using a hybrid flow cell configuration, (c) high temperature NRR with water oxidation in a SOEL, (d) high temperature NRR with hydrogen oxidation in a SOEL, (e) Li-mediated NRR with hydrogen oxidation in a hybrid flow cell configuration.

We defined the energy efficiency (EE) of the electrolyzer or the entire process as the ratio between the LHV of NH_3_ (18.6 GJ per tNH_3_) and the total energy input (*e*_in_):5
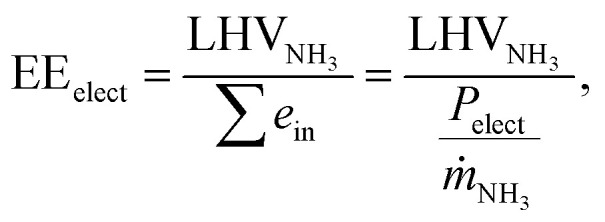
where *ṁ*_NH_3__ is the production capacity in tonnes per seconds. Fig. 1 shows basic representations of the envisioned process flow diagrams (PFDs). More detailed PFDs are illustrated in Fig. S1–S6 (ESI†), including stream data and equipment specifications, which can be found in Tables S1–S14 (ESI†). The processes can be generalized into three segments: (1) feed pretreatment, (2) NH_3_ synthesis and (3) NH_3_ separation. The exact unit operations for each segment depend on the NH_3_ synthesis configuration.

Methane-fed Haber–Bosch with a single SMR unit, electrified Haber–Bosch and the NRR based processes require an air separation unit (ASU) to produce a high purity N_2_ feed for the synthesis loop and the NRR electrolyzers. Otherwise, small quantities of oxygen will poison the iron catalyst (in the synthesis reactor) or will initiate the oxygen reduction reaction (ORR) as a parasitic side reaction in the NRR electrolyzers. The selection of a specific ASU technology depends on the N_2_ capacity, where pressure swing adsorption (PSA) is more economical below 500 tN_2_ per day and cryogenic distillation above 500 tN_2_ per day.^22^ If a PSA is integrated in the process, the oxygen waste stream can not be sold as commodity because its purity is below market grade (<99.9%), which is not the case for cryogenic distillation. Argon is also a by product of the ASU, but is excluded from the analysis.

For the electrified Haber–Bosch process (see Fig. 1a), an alkaline electrolyzer (AEL) or proton exchange membrane electrolyzer (PEMEL) is integrated in the model to produce H_2_. The main advantage of PEMEL over AEL is its load flexibility, compact design, high pressure operation and a better energy efficiency, but PEMEL is significantly more costly.^23^ Therefore, it is valuable to understand the economic benefit of both scenarios. The implementation of a solid-oxide electrolyzer (SOEL) in the electrified Haber–Bosch process was excluded from this analysis because of reported issues with thermal degradation of the electrode material reducing its overall lifetime (<5000 h) in comparison with AEL (55 000–96 000 h) and PEMEL (60 000–100 000 h), respectively.^8,23^ Additionally, it is estimated that the capital costs of the SOEL will be relatively high due to expensive material costs, which limits its economic attractiveness as a water electrolyzer for the electrified Haber–Bosch process.^24,25^

N_2_ and H_2_ are both pressurized to 155 bar in an intercooled multi-stage compressor before entering the Haber–Bosch reactor. The thermocatalytic NH_3_ reaction is exothermic (−53.8 kJ mol^−1^ at 155 bar, 400 °C) and excess heat can be harnessed to pre-heat the reactor feed. Hence, no additional heat source is required. The N_2_/H_2_/NH_3_ mixture is cooled down to −5 °C and separated by flash evaporation into a 99.5 mol% NH_3_ product stream and 4 mol% NH_3_/N_2_/H_2_ gaseous mixture. The latter is recycled back to the compressor and mixed with the other feed gases.

Fig. 1b illustrates our proposed design for aqueous based electrochemical ammonia synthesis at ambient conditions (aqueous NRR). The aqueous NRR electrolyzer is modelled as a hybrid gas–liquid flow cell with a N_2_ gas compartment, a catholyte and anolyte compartment. A gas diffusion electrode (GDE) separates the gas and liquid compartments. The NRR occurs at the triple phase boundary (TPB) at the liquid catholyte side of the GDE, where, it is assumed that produced NH_3_ will directly dissolve into the electrolyte due to its high solubility (540 g per L_H_2_O_ at 20 °C).^26^ H_2_ is formed as a byproduct at the TPB and flows back through the GDE into the gas compartment. Two design alternatives for the utilization of the gaseous N_2_/H_2_ product stream were considered; the N_2_/H_2_ product stream can simply be purged (referred as “purge scenario”) or partly separated *via* the N_2_/H_2_ PSA to sell H_2_ as a commodity (“PSA scenario”). However, N_2_/H_2_ separation is non-trivial and may require at least 60 mol% H_2_ in the PSA feed to be technically feasible.^27^ Therefore, we incorporated an accumulation loop in the PSA scenario that recycles a N_2_/H_2_ mixture back to the GDE to satisfy this requirement (see Fig. S3, ESI†). Another potential strategy is to harvest the energy of the N_2_/H_2_ mixture by the generation of heat *via* combustion. The latter is not desirable because N_2_ forms NO_*x*_-related greenhouse gases upon combustion,^28^ which require additional DeNO_*x*_ installations. Dissolved NH_3_ in 1 M KOH aqueous solution is separated by distillation with a distillate purity of 99.5 mol% and 99.9% NH_3_ recovery. The energy consumption of the column depends mainly on the NH_3_ composition in the feed (see Fig. S7, ESI†). From our analysis, a minimum of 10 mol% NH_3_ is implemented to limit the distillation energy consumption.

High temperature NRR occurs in a solid oxide electrolyzer (SOEL) that operates at 550 °C and 1 atm. This pathway is divided into two similar process variations, wherein the SOEL reduces N_2_ with water oxidation (NRR SOEL with water oxidation, Fig. 1c) or hydrogen oxidation including an additional water electrolyzer for H_2_ production (NRR SOEL with hydrogen oxidation, Fig. 1d). The SOEL operates in thermoneutral mode, meaning that the heat balance within the cell is in equilibrium.^23^ A heat exchanger network is designed to minimize the required heat input for the SOEL feed, by integrating inlet with outlet streams, as can be seen in the PFDs (Fig. S4 and S5, ESI†). The NH_3_/N_2_/H_2_ product mixture cannot be separated by flash evaporation because the stream is at atmospheric pressure. NH_3_ condensation is only techno-economically feasible when higher pressures (≥150 bar) are considered (as for the electrified Haber–Bosch process).^8^ For low pressure systems, adsorption by zeolites or absorption in alkaline earth metal salts are poised as promising separation technologies.^29^ In this process, NH_3_ is separated by an adsorption step with an NH_3_ product purity of 99.5 mol% and recovery of 90%. The other 10% cannot be recycled because NH_3_ will decompose directly (>400 °C). Due to the complexity of the heat integration system, it was not possible to further separate the N_2_/H_2_ stream in a similar fashion as the aqueous NRR process (PSA scenario).

The electrolyzer design in the Li-mediated NRR process (Fig. 1e) is inspired on the continuous flow cell recently developed by Chorkendorff and coworkers.^30^ The electrolyzer is modelled as a hybrid flow cell with two gas compartments (for N_2_ and H_2_), which are separated by an organic electrolyte that contains 1 M lithium bis(fluorosulfonyl)imide (LiFSI) in 0.25 vol% EtOH/THF. The gas and liquid compartments are separated by GDEs. We selected 1 M LiFSI due to its high conductivity with respect to other Li salts, while we are aware that the highest FEs in a batch-type cell were obtained with 2 M LiBF_4_ and 2 M LiTFSI.^31,32^ Again, it is assumed that NH_3_ will directly dissolve in the organic electrolyte and can be separated by distillation with a distillate purity of 99.5 mol% and 99.5% NH_3_ recovery.

## Identification of energy losses in different NRR electrolyzers

**Fig. 2:**
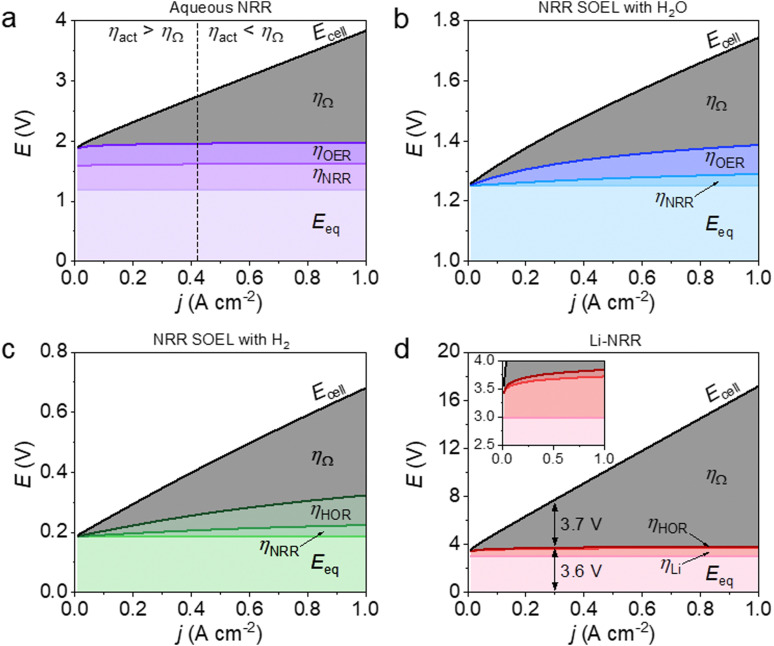
Build-up of the current–voltage relationship by stacking the individual voltage contributions of the equilibrium potentials, overpotentials and ohmic losses for: (a) aqueous NRR, (b) NRR SOEL with water, (c) NRR SOEL with hydrogen and (d) Li-NRR. Relevant input data is listed in Table S15 (ESI†) and assumptions are discussed in “Process Design Assumptions and Descriptions” in the main text and “Supplemental methods” in the ESI.†

The energetics of the electrolyzer often dominate the overall energy input of an electrochemical process. Here we used a simple modelling approach by using the Nernst law, approximations from the Butler–Volmer equation and expressions for the ohmic losses, to estimate the current–voltage relationship (Fig. 2) and energy losses of the considered NRR electrolyzers (with eqn (1)–(5), respectively). This gives us a preliminary estimate of the energy efficiency of each process and how this relates to the energy efficiency of SMR Haber–Bosch.

**Fig. 3:**
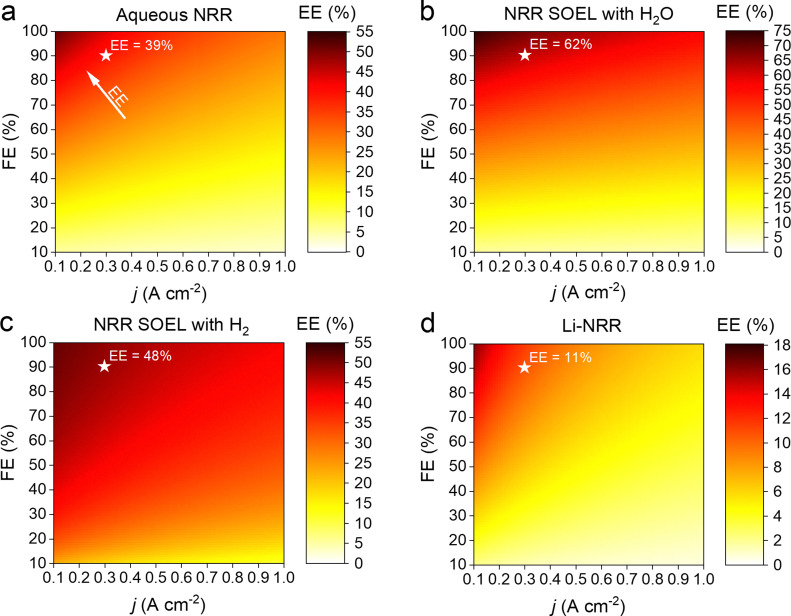
Contour plots of the electrolyzer's EE as a function of *j* and FE for: (a) aqueous NRR at ambient conditions, (b) high temperature NRR in a SOEL with water oxidation, (c) high temperature NRR in a SOEL with hydrogen oxidation and (d) Li-NRR with hydrogen oxidation. Both (c) and (d) include an additional energy input term for H_2_ production with an alkaline H_2_O electrolyzer (28.4 GJ per tNH_3_ based on the commercial AEL type “A484” of Nel Hydrogen).^23^ Star symbol indicates the calculated EE at the US DoE Arpa-e electrolyzer aspirational values (0.3 A cm^−2^ and 90% FE). It is important to note that the plots imply a low *j* (<0.1 A cm^−2^) seems appealing. However, there is an economic trade-off between the EE and *j*, where the former has an effect on the OPEX and the latter on the capital costs. The optimal electrolyzer operation parameters will be discussed in the economic analysis.

An important advantage of the aqueous NRR compared to the electrified Haber–Bosch is the process intensification step, where NH_3_ can potentially be synthesized in a single electrolyzer with a considerably lower *E*_0_ (1.17 V) *versus* 1.23 V for H_2_O electrolysis, with a thermodynamic minimum of 19.9 GJ per tNH_3_ with respect to 21.3 GJ per tNH_3_ for H_2_O electrolysis (based on the LHV of stoichiometric amount of H_2_).^8^ However, NRR involves six proton-coupled electron transfer steps, where the intermediates impose thermodynamic constraints. As a result, a minimum barrier in the form of an *η*_NRR_ (0.4–0.6 V) is required to activate the reaction.^5,20,33^ The *j*–*E* curve in Fig. 2a indicates that below 0.42 A cm^−2^, the activation overpotentials (*η*_NRR_ and *η*_OER_) are higher than the ohmic losses (*η*_Ω_). At higher *j*, ohmic losses become more significant due to the relatively low conductivity of the 1 M KOH electrolyte (0.215 S cm^−1^ at 25 °C). At 0.3 A cm^−2^ and 90% FE, taken as electrolyzer aspirational values from the US Department of Energy ARPA-e REFUEL program,^34^ the ohmic losses are so severe that the electrolyzer's EE decreases to 39% (see Fig. 3a). This can partly be circumvented by considering a 25 wt% KOH aqueous solution as a more conductive electrolyte, thereby increasing the EE with +9%.

An advantage of high temperature NRR is the lower activation barrier for both the NRR (0.04 V at 1 A cm^−2^) and the H_2_O oxidation reaction (0.1 V at 1 A cm^−2^) as illustrated in Fig. 2b. In contrast to a water SOEL, the *E*_0_ of NRR increases with temperature (1.17 V at 25 °C to 1.21 V at 550 °C) due to a negative change in reaction entropy (see Fig. S8–S10, ESI†). The main reason why SOELs operate at such high temperatures is to increase the conductivity of the solid electrolytes. Ce_0.8_Sm_0.2_O_2_ is commonly used as an electrolyte and it has a conductivity of 0.014 S cm^−1^ at 650 °C, which is an order of magnitude lower than 1 M KOH (0.215 S cm^−1^), but this is typically compensated by using a thin slab of 0.05 mm. At 0.3 A cm^−2^, the voltage losses account for 13% of *E*_cell_, thus the *E*_0_ dictates the energy efficiency. By substituting the oxygen evolution reaction (OER) for the hydrogen oxidation reaction (HOR), the *E*_0_ decreases to 0.19 V (see Fig. 2c). Nevertheless, the net energy gain of the cell voltage is compensated by the additional requirement for H_2_ (alkaline water electrolysis consumes 28.4 GJ per tNH_3_ based on the Nel Hydrogen type “A485”). Fig. 3b and c clearly demonstrates that the indirect approach is more energy intensive (without considering the up- and downstream units), where the EE of NRR SOEL with water is +14% higher than NRR SOEL with H_2_ (including AEL).

Li-mediated NRR is fundamentally energy intensive due to the required presence of metallic Li, with an *E*_0_ of −3 V *vs.* SHE for Li-plating. This results in a thermodynamic minimum of 51 GJ per tNH_3_ when Li-plating is combined with hydrogen oxidation (at 0 V *vs.* SHE), which is already 16 GJ per tNH_3_ higher than electrified Haber–Bosch. Fig. 2d shows that the actual energy input will be even more severe due to activation overpotentials and ohmic losses. Among the Li-salts, Li bis(trifluoromethanesulfonyl)imide (LiTFSI) and Li bis(fluorosulfonyl)imide (LiFSI) are reported as having the highest conductivities in organic solvents and contain fluorinated functional groups. Especially the latter is important for the formation of a stable and selective SEI.^31^ By assuming 1 M LiFSI dissolved in 0.1 M EtOH/THF as electrolyte with a conductivity of 0.015 S cm^−1^ (electrolyte gap = 2 mm), the ohmic resistance becomes so significant, that ohmic losses start to dominate *E*_cell_ at current densities >0.3 A cm^−2^. Unsurprisingly, the EE diagram in Fig. 3d indicates that Li-NRR (including AEL for green H_2_ production) has the lowest EE in comparison with other NRR electrolyzers.

## Energy losses in sustainable NH_3_ processes

**Fig. 4:**
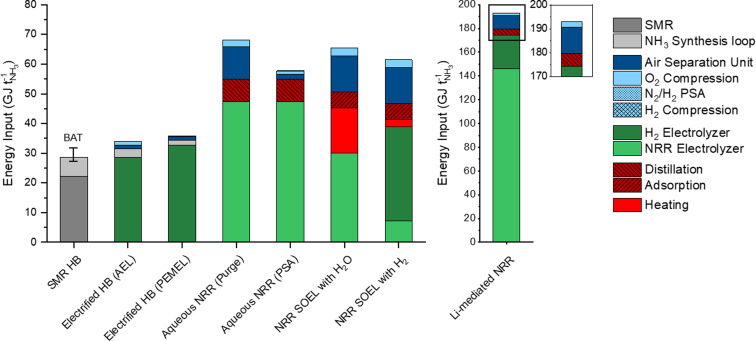
Indicative overview of the estimated energy input of each process. Values above the LHV of NH_3_ (18.6 GJ per tNH_3_) can be considered as energy losses. The energy input of the NRR electrolyzer was calculated at the aspirational values (*j* = 0.3 A cm^−2^ and FE = 90%). Generally, the energy losses will increase at FE < 90% and *j* > 0.3 A cm^−2^.

The total energy inputs for the NH_3_ production processes, including the electrolyzers, upstream and downstream unit operations, are illustrated in Fig. 4. For comparison, the energy requirement of SMR Haber–Bosch is also included and was taken from previous literature reports.^8,35^ The energy input of the AEL (28.4 GJ per tNH_3_) and PEMEL (32.8 GJ per tNH_3_) for the electrified Haber–Bosch process and indirect NRR pathways are based on commercially available models from Nel Hydrogen (A485) and Siemens Energy (Silyzer 300) with an EE of 75% and 65% (using the LHV of the stoichiometric amount of H_2_).^23^ The energy requirements for the NRR electrolyzers were calculated with our electrochemical model using the US DoE ARPA-e aspirational values (0.3 A cm^−2^ and 90% FE) as input parameters. The following highlights the main findings from our energy analysis and discusses several energy saving strategies.

The energy consumption in the synthesis loop is significantly higher in the methane fed Haber–Bosch process (6.45 GJ per tNH_3_) than in the electrolysis based process.^8,35^ These losses in SMR Haber–Bosch can be assigned to low efficiencies of the steam turbine cycles (42–48%) that drive the feed-gas, recycle and refrigeration compressors.^8^ Additional losses of 1.7 GJ per tNH_3_ are associated with the necessity to purge a part of the product mixture for the recycle loop. In electrified Haber–Bosch, the losses in the NH_3_ synthesis loop are solely related to the compressor duty since there is no requirement for purging. These compressors need to be electrically driven due to the absence of a high pressure steam network in the electrified Haber–Bosch process. These types of compressors have a significantly higher driver efficiency (up to 95%), meaning that losses in the synthesis loop are limited. Additionally, commercially available PEMEL systems have the ability to produce H_2_ at 35–50 bar, which can save up to 56% of the compressor duty. After the synthesis loop, the NH_3_/N_2_/H_2_ mixture is separated by condensation (typically at −5 °C and 145 bar).^36^ Although the temperature gradient between the condenser and the synthesis reactor seems large, heat integration in the synthesis loop (Section S4.8 in the ESI†) recovers most of the heating and cooling duties. The energy input of the ASU is directly proportional to the stoichiometric demand of N_2_ for the reactor because unreacted N_2_ is separated and recycled back to the synthesis loop. Therefore, the ASU energy demand is limited to 1.3 GJ per tNH_3_. Although the electrified version of the Haber–Bosch is less energy efficient (33.9 GJ per tNH_3_) than the best available technology (BAT) (27.4–31.8 GJ per tNH_3_) due to the water electrolyzers, it is expected that innovations in the PEMEL system will improve the EE in the foreseeable future.^37^

The aqueous NRR electrolyzer consumes 47.4 GJ per tNH_3_, which accounts for 57% of the total energy loss. It is assumed that NH_3_ dissolves directly into the electrolyte after electrosynthesis and has to be separated downstream by distillation. The NH_3_ feed composition plays an important role in determining the energy input of the distillation unit. Fig. S7 (ESI†) shows that the reboiler duty decays exponentially with increasing NH_3_ feed concentration, with a minimum at approximately 10 mol%. Even at 10 mol%, the duty of both the reboiler and condenser are still considerable (7.5 GJ per tNH_3_). Implementing a N_2_ recycle stream can reduce the demand of “fresh” N_2_ from the ASU. To study this effect, we considered two process design alternatives for the N_2_/H_2_ product stream, where the product stream is simply purged (Fig. S2, ESI†) or separated by a PSA with N_2_ recycling and H_2_ recovery (Fig. S3, ESI†). The purge scenario shows a 7-fold increase of the ASU duty (10.9 GJ per tNH_3_) with respect to the PSA scenario. This indicates the importance of a recycle stream in order to save up to −9.3 GJ per tNH_3_.

NRR SOEL with water oxidation is the most energy efficient NRR electrolyzer (62%), meaning that the voltage losses at high temperature electrolysis are minimal. However, this is a false minimum since roughly 15 GJ per tNH_3_ of additional heat is necessary to sustain the NRR SOEL operational temperature (550 °C). Even though heat integration is included, there is a mismatch between the heat capacities of the reactants (N_2_, H_2_O_(g)_) and products (N_2_, NH_3_, O_2_). This implies that external heat must be supplied. By combining the additional heat and energy input of the SOEL, the EE decreases to 41%. Unfortunately, 3.8 GJ per tNH_3_ of this heat is labelled as “high quality heat” (to raise the feed temperature to 550 °C), which is challenging to obtain by steam, but can be harnessed from electric heating or waste heat from neighbouring chemical plants.^38^ For a stand-alone plant, a “green” furnace based on biogas or H_2_ from water electrolysis are also possible options. For now, it is assumed that the heat is imported from neighbouring chemical industries for the natural gas price. To save energy on the ASU (−10.2 GJ per tNH_3_), the N_2_ enriched waste stream from the separation step can in principle be recycled and mixed with the reactant stream. However, an additional 3.3 GJ per tNH_3_ of high quality heat is necessary to elevate the recycle temperature from 200 °C to 550 °C. This means that there is a trade-off between the import of heat and ASU energy savings. For simplicity, the recycle stream is excluded from further analysis. Separating the low pressure N_2_/H_2_/NH_3_ product mixture of the NRR SOEL by condensation is not economically attractive due to the excessive compressor costs.^8^ While still in the research phase, adsorption with zeolites is a promising approach for low pressure NH_3_ separation.^29,39^ The energy input for separation by adsorption (5.5 GJ per tNH_3_) depends on the heat of adsorption (2.76 GJ per tNH_3_), feed compression (2.32 GJ per tNH_3_) and desorption vacuum swing (0.46 GJ per tNH_3_). The compression duty is required to overcome the large pressure gradient (around 2 bar) across the densely packed column. The heat of adsorption depends on the interaction strength between the adsorbent and adsorbate. Since NH_3_ binds strongly to zeolites, a significant amount of heat must be supplied for desorption, although this is much less than would be required with metal halides.^29^

The enormous energy input of the Li-NRR electrolyzer (146 GJ per tNH_3_) accounts for 84% of the process losses, which are inherently related to the Li-plating potential and the low conductive nature of organic Li-salt electrolytes. These specific physical properties cannot be improved, but the electrolyte gap between the electrodes can be minimized by implementing a zero-gap membrane electrode assembly (MEA).^40^ Fig. S11 (ESI†) indicates that the electrolyzer EE can be increased by 8% when the electrolyte gap is completely eliminated. An alternative strategy is to find an active mediator with a lower plating potential than Li.^41^ Ca has recently been identified as an active mediator besides Li.^42^ However, the net energy gain of using Ca is limited since its plating potential only differs ∼0.2 V from Li. Theoretical work of Bagger, Stephens and coworkers have proposed Mg and Al as promising alternatives.^43,44^ Experimental work performed by Krebsz *et al.* proved that electroplated Mg is able to activate N_2_ by forming a surface layer of MgN_*x*_, which can be hydrolysed into ammonia *via* a two-step approach.^45^ An overview of the *E*_0_ and Δ*G* of these mediators paired with hydrogen oxidation is displayed in Fig. S12 (ESI†). When assuming an *E*_0_ of −2.36 V *vs.* SHE for Mg plating and 0 V *vs.* SHE for hydrogen oxidation, the thermodynamic minimum of this electrolyzer would be 40.2 GJ per tNH_3_, which is still relatively high in comparison with other NRR electrolyzers. Al is in terms of its low plating potential the most propitious element, but remains yet to be experimentally explored and verified.

## Assumptions for the techno-economic analysis

The techno-economic analysis is based on small scale NH_3_ plants with the same capacity (91 tNH_3_ per day) that operate 333 days per year, with a life time of 20 years for electrolysis based ammonia processes and 40 years for SMR Haber–Bosch. It is assumed that the electrolyzer stacks do not have to be replaced during the life time of the plant. The investment cost for a 91 tNH_3_ per day SMR Haber–Bosch plant is $_2022_ 936 M taken from ref. 7 (with inflation correction). The capital costs of the sustainable ammonia processes were estimated based on the equipment costs of all the process units in the plant. Standard process equipment, such as compressors, heat exchangers, pumps and columns are designed based on industrial heuristics. The equipment costs (*C*_E_) were calculated *via* different equipment capacity (*S*) correlation functions:6
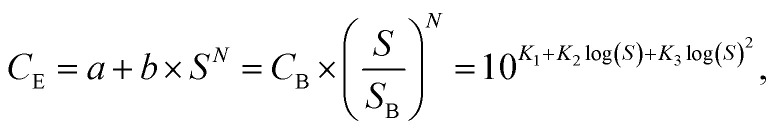
where the coefficients (*a*, *b*, *N*, *K*_1_, *K*_2_, *K*_3_) are tabulated in chemical engineering handbooks and summarized in Table S16 (ESI†).^46–49^ The costs for cryogenic distillation (ASU) and N_2_/H_2_ PSA were calculated based on the 6th tenth rule with base estimates from Morgan *et al.* and Mivechian *et al.*^50,51^ For the ASU PSA, a modular cost estimation was applied (*N* = 1) with a base estimate from Banares-Alcantara *et al.*^52^ The equipment cost of the electric steam boiler was assumed to be $60 per kW.^53,54^

**Fig. 5:**
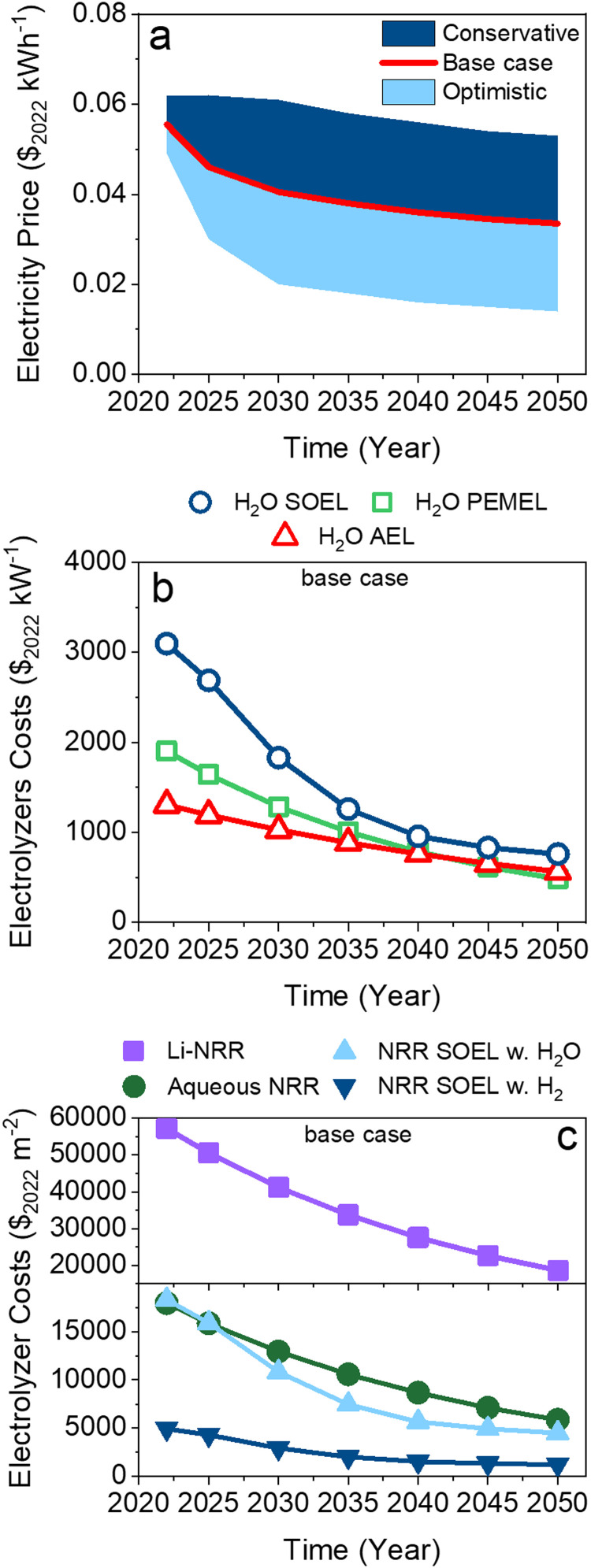
Price projections for; (a) renewable electricity for a conservative, base case and optimistic scenario; (b) base case water electolyzer costs of PEMEL, AEL and SOEL in $ per unit power; (c) base case electrolyzer costs of NRR electrolyzers in $ per unit area. Data used for these figures is listed in Table S17 (ESI†).

The electrolyzer costs, electricity and hydrogen prices for the base case scenario are inter- and extrapolated from 2022–2050 cost projections taken from numerous available sources (see Fig. 5 and Table S17 for referencing, ESI†).^11,55–58^ Other base case parameters, such as the price of O_2_ ($0.14 per kg),^59^ natural gas ($3.78 per GJ),^60^ H_2_O ($7.5 per m^3^),^61^ CO_2_ tax ($58 per tCO_2_),^62^ labor and O&M (3% of total capital costs) are kept constant.^23,36^ These numbers are mostly based on North American price indexing if available. For each cost parameter, more conservative and optimistic price projections reported by other literature sources were also included in the analysis (see Table S17 for more details, ESI†). This wide range of model input data allows us to predict under what conditions green NH_3_ becomes feasible and in which timeframe.

It is important to note that there is no available capital cost data of NRR electrolyzers. Therefore, their capital costs were derived from commercial H_2_O electrolyzers and compared with other cost data from the literature for validation.^25,63^ Electrolyzer costs, often expressed in $ per unit power, were converted with their respective power density (kW per m^2^) to $ per unit area to include the effect of the current density on the economics. The power density is related to the *j–E* characteristics of the electrolyzer, hence the $ per m^2^ is different for each particular system as can be seen in Fig. 5c. Additional statements regarding the electrolyzer capital cost assumptions and an extended discussion on the calculations are available in the ESI† (Section S4.9).

The inflation was corrected with the chemical engineering plant cost index (CEPCI). The total capital cost was estimated from the equipment cost with the Lang factorial method.^47^ These factors include the installation costs, contingency and working capital (more details can be found in Table S18, ESI†). It is important to note that the installation costs in the “inside battery limit” (ISBL) are temperature, pressure and material dependent, therefore the ISBL was calculated for each piece of equipment independently. The electrolyzer installation costs were not estimated *via* the Lang factors, but were assumed to be 10% of the equipment costs.^23^ General assumptions regarding the OPEX are mentioned in Table S19 (ESI†). The end-of-life net present value (NPV) was calculated using eqn (7) with 25% tax rate, 25% salvage value and a linear depreciation scheme by taking the cumulative sum of the cash flow (CF) discounted with 4.28% interest rate (median between 1954–2023 US interest rates):^64^7
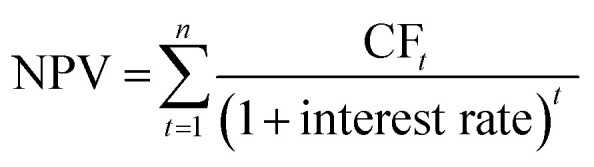


The levelized cost of ammonia (LCOA) is used as an indicator to estimate and compare the economic feasibility of the different ammonia production plants. The LCOA is a function of the product revenue present value (eqn (8)), which can be obtained by adjusting the NH_3_ selling price until the NPV is equal to zero.^15,65^ The total capital cost is incurred during the first construction year of the plant (*t* = 0), where it assumed that the plant is fully operational at *t* ≥ 1.

8 NPV = 0 = Product revenue PV (LCOA) − Operating cost PV − total capital costs

## Economic comparison of methane-based *versus* electrified Haber–Bosch

**Fig. 6:**
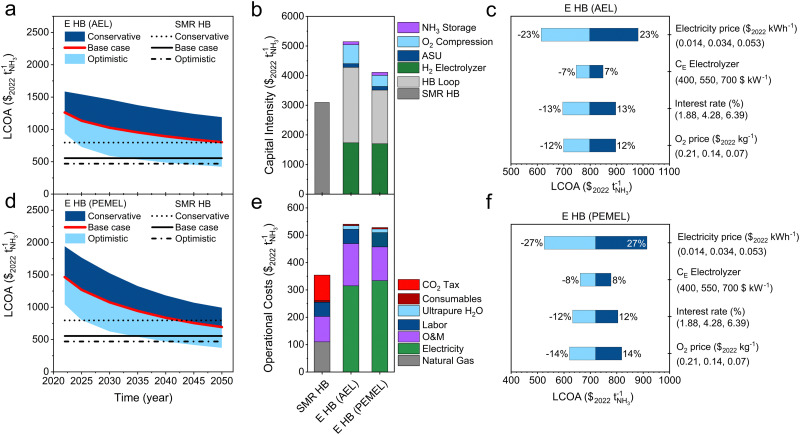
Economic analysis of electrified Haber–Bosch. (a) Levelized cost of ammonia (LCOA) of electrified Haber–Bosch with AEL at conservative, base case and conservative assumptions. (b) Capital intensity calculated with the base case assumption in 2050. (c) Sensitivity analysis of electrified Haber–Bosch with AEL. (d) LCOA of electrified Haber–Bosch with PEMEL at conservative, base case and conservative assumptions. (e) Operational costs estimated with the base case assumptions in 2050. (f) Sensitivity analysis of electrified Haber–Bosch with PEMEL. Black lines in (a) and (d) indicate the LCOA of SMR HB calculated with optimistic (NG price = $2.79 per GJ, CO_2_ tax = $23 per tCO_2_), base case (NG price = $3.77 per GJ, CO_2_ tax = $58 per tCO_2_) and conservative (NG price = $5.66 per GJ, CO_2_ tax = $175 per tCO_2_) price scenarios.

With the implementation of the base case assumptions, the LCOA of the small scale SMR Haber–Bosch plant is $555 per tNH_3_ as illustrated in Fig. 6a and d, which is in line with previous literature reports.^15,66^ These figures indicate that the price for grey ammonia (from SMR Haber–Bosch) remains considerably lower than ammonia from sustainable sources, such as the electrified Haber–Bosch process. The sensitivity analysis implies that the natural gas price and carbon tax are the main cost drivers for SMR Haber–Bosch (Fig. S13, ESI†). For a while, these plants have benefitted from relatively low natural gas prices (∼$3 per GJ), but the 2022 energy crisis in Europe has shown that SMR Haber–Bosch can be vulnerable.^67^ The US EIA states that natural gas prices can rise above $5 per GJ by 2050 which will put a lot of pressure on conventional Haber–Bosch economics.^17^ Additionally, societies demand more compensation for emitted greenhouse gases from the chemical industries in the form of an emission trading system or tax to stimulate the transition towards renewable alternatives. The latest IPCC report predicts that a carbon tax of $58 per tCO_2_ is necessary to incentivise the implementation of carbon capture and storage technologies by the chemical industry.^62^ Other economists and climate scientists claim that the CO_2_ tax should increase even further to $174–417 per tCO_2_.^68–70^ Hence, a more conservative price scenario ($175 per tCO_2_, $5.66 per GJ) is necessary to incentivise a shift towards carbon free NH_3_.

Fig. 6a and d illustrate that electrified Haber–Bosch is too expensive under the current economic conditions (in 2022) compared with SMR Haber–Bosch, even when considering conservative price assumptions (∼$800 per tNH_3_). The sensitivity analysis in Fig. 6c and f show that the electricity price has the largest influence on the LCOA. By saving $0.01 per kW h on the OPEX, the LCOA reduces with roughly $100 per tNH_3_ (electrified HB with PEMEL), while a cost reduction of $100 per kW of the stack, lowers the LCOA with only $60 per tNH_3_ (approximately −5%). When considering the base case cost projection, electrified HB with PEMEL becomes cost competitive with SMR HB at $615 per kW (PEMEL investment costs) and $0.035 per kW h (electricity price). This means that the manufacturing of PEMEL systems and the cost of electricity has to be reduced by −68% and −38% within the upcoming decades. Other combinations of electrolyzer CAPEX and electricity prices can also lead to cost competitive ammonia production (see Fig. S14b, ESI†). Replacing PEMEL with AEL demands an additional investment of 29% for larger compressors because commercially available AELs deliver H_2_ at atmospheric pressure. Therefore, PEMEL is in this context a more suitable source for H_2_.

## Economic analysis of aqueous NRR at ambient conditions

**Fig. 7:**
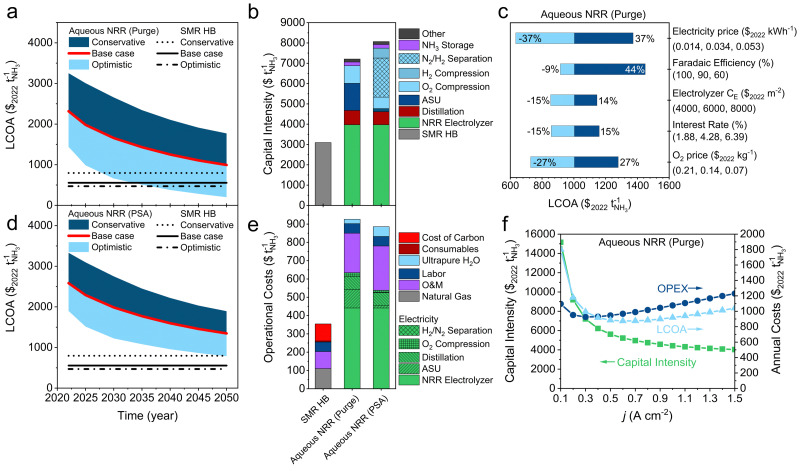
Economic analysis of aqueous NRR at ambient conditions. (a) LCOA of aqueous NRR with a H_2_ purge. (b) Capital intensity calculated with the base case assumptions in 2050. (c) Sensitivity analysis of aqueous NRR (purge) at a constant *j* of 0.3 A cm^−2^. (d) LCOA of aqueous NRR with H_2_ recovery (PSA). (e) Operational costs using the base case assumptions in 2050. (f) Current density as a function of the capital intensity, OPEX and the LCOA for aqueous NRR (purge) at FE of 90% and base case assumptions in 2050. Black lines in (a) and (d) indicate the LCOA of SMR HB calculated with optimistic, base case and conservative price scenarios. (a), (b), (d) and (e) The ARPA-e electrolyzer aspirational values (FE = 90%, *j* = 0.3 A cm^−2^) were used for the economic analysis.

The LCOA of aqueous NRR at ambient conditions for the process scheme with a N_2_/H_2_ purge or PSA recovery unit are under the base case conditions not competitive with SMR Haber–Bosch, and require more optimistic price projections (see Fig. 7a and d). The main economic issue with aqueous NRR is the relatively high operational costs (∼$450 per tNH_3_) related to the electrolyzer due to unavoidable energy losses by activation overpotentials and ohmic losses. Consequently, Fig. 7e indicates that approximately 70% of the electricity costs and 50% of the OPEX are associated with the electrolyzer's electrical input. The capital costs of the NRR electrolyzer comprises 55% of the total capital cost and is ∼$2000 per tNH_3_ more expensive than a PEMEL. The latter is justifiable since a hybrid flow cell configuration is more complex in operation due to potential flooding issues of the GDE. This problem is typically circumvented by carefully controlling the pressure gradient over the GDE.^71^ The hybrid flow cell does also consumes more power per tNH_3_, therefore, the balance of plant (BoP) can be higher due to additional pressure regulators, rectifiers with a larger capacity, and miscellaneous auxiliary equipment.

The process design with a N_2_/H_2_ purge (Fig. S2, ESI†), hence without a N_2_ recycle, consumes evidently more “fresh” N_2_ feedstock from the ASU. Consequently, the CAPEX of the ASU (18% of total capital costs) is roughly $1200 per tNH_3_ more expensive than when a N_2_ recycle is considered. For electrified Haber–Bosch, recycling N_2_ or a N_2_/H_2_ mixture is more straightforward because N_2_/H_2_ can be re-compressed, mixed with the N_2_/H_2_ feedstock and fed into the synthesis reactor. A mixture of N_2_/H_2_ can in principle be used as feed for the NRR electrolyzer since H_2_ is inert. But, if H_2_ is not separated from the recycle loop, it will accumulate, cross-over to the anode and form an explosive mixture with O_2_ or recombine into water. To realize a H_2_ separation step, an additional capital injection of 11% has to be invested into a N_2_/H_2_ PSA ($638 per tNH_3_), storage infrastructure for recycle buffering ($1286 per tNH_3_) and H_2_ compressors ($488 per tNH_3_) with a −4% gain of the OPEX. It becomes clear that by comparing the LCOA trend in Fig. 7a and d, purging the N_2_/H_2_ product stream is from an economic point of view more attractive because N_2_/H_2_ separation is considered to be technically challenging, wherein a minimum feed composition of 60 mol% H_2_/N_2_ is typically required with very low H_2_ recoveries (∼50%).^27^

Steering towards H_2_ production with NH_3_ as a by-product (FE < 50%) is not preferred because NH_3_ has more intrinsic value, and H_2_ can be produced more efficiently in a PEMEL or AEL. This indicates that steering towards a near unity ammonia FE should be the main objective as is supported by our sensitivity analysis (Fig. 7c). When considering the purge scenario, O_2_ market price fluctuations have a substantial effect on the LCOA (±27%), especially in comparison with electrified Haber–Bosch (Fig. 6c and f). This is related to the vast quantities of O_2_ (230 tonnes per day) that are being produced by the cryogenic distillation unit due to the large demand for N_2_. In case the location of the plant does not allow O_2_ export to the market, the LCOA increases by ∼$550 per tNH_3_.

The necessary cost reductions to reach SMR Haber–Bosch parity (∼$800 per tNH_3_) are highlighted in Fig. S14c (ESI†). It becomes clear that very optimistic electrolyzer costs ($5600 per m^2^) and electricity prices ($0.025 per kW h) have to be realized for cost competitive NH_3_ production. Additionally, the LCOA is heavily influenced by the electrolyzer performance metrics. Fig. 7f presents an optimal *j* window between 0.5–0.9 A cm^−2^, where the LCOA is approaching its minima. By assuming an electrolyzer CAPEX of $5850 per m^2^ (base case) and a very optimistic electricity price of $0.02 per kW h, a “minimum” FE as a function of the *j* can be estimated. The results are displayed in Fig. S15 (ESI†) and highlights three regions: FE > 80% at 0.3 A cm^−2^, FE > 70% at 0.4 A cm^−2^ and FE > 65% between 0.5–1 A cm^−2^. Operating at *j* < 0.3 A cm^−2^ is not preferable because the capital costs increase exponentially with the electrode area. The earlier used aspirational values from the ARPA-e REFUEL program (90% FE, 0.3 A cm^−2^) are reasonable and fall within the estimated range. Nevertheless, this analysis extends the aqueous NRR opportunity window and can be used as guideline for experimentalists.

## Economic analysis of NRR at elevated temperatures

**Fig. 8:**
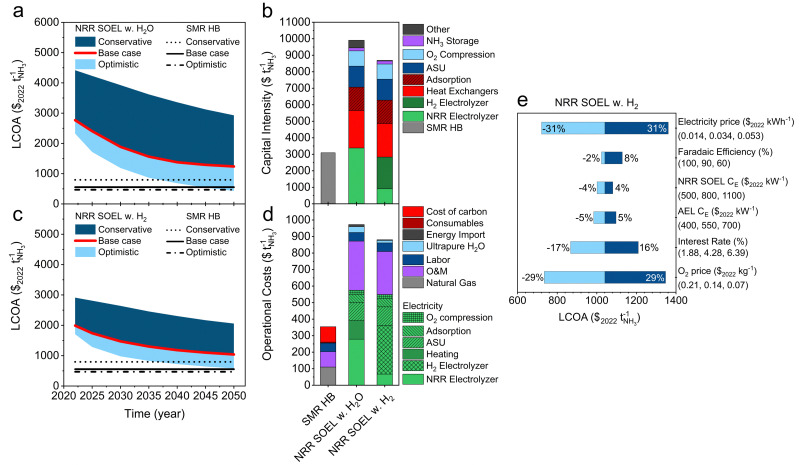
Economic analysis of high temperature NRR. (a) LCOA of NRR SOEL with water oxidation. (b) Capital intensity calculated with the base case assumptions in 2050. (c) LCOA of NRR SOEL with hydrogen oxidation. (d) Operational costs calculated with the base case assumptions in 2050. (e) Sensitivity analysis of NRR SOEL with hydrogen oxidation at a constant *j* of 0.3 A cm^−2^. Black lines in (a) and (d) indicate the LCOA of SMR HB calculated with optimistic, base case and conservative price scenarios. (a)–(d) The ARPA-e electrolyzer aspirational values (FE = 90%, *j* = 0.3 A cm^−2^) were used for the economic analysis.

Fig. 8a and c show that both the NRR SOEL with water and with hydrogen pathways are under the base case assumptions not cost competitive with SMR Haber–Bosch, and require a more optimistic economic scenario. In contrast with aqueous NRR, the CAPEX and OPEX of the NRR SOEL unit are not dominating the plant costs. The majority of the investment is related to conventional process units, such as heat exchangers, air separation units and adsorption columns, which account for roughly 65% of the fixed capital costs. According to our analysis, NRR SOEL with hydrogen (containing two electrolyzers) is more cost effective than the NRR SOEL with water. This is counterintuitive, but can be explained based on differences in the heat integration and the electrolyzer capital costs. NRR SOEL with hydrogen has almost an ideal heat integration scenario, limiting the demand for high quality heat, which saves up to $100 per tNH_3_ on the OPEX. Additionally, Fig. 8b illustrates that the NRR SOEL unit with hydrogen oxidation is less capital intensive due to its lower power density (1.6 *versus* 5.9 kW per m^2^ for NRR SOEL with water oxidation), which directly affects the BoP as discussed previously.

The sensitivity analysis in Fig. 8e and Fig. S16 (ESI†) indicates that the electricity price has the largest effect on the feasibility. Unsurprisingly, the absence of a N_2_ recycle in the high temperature process means that the ASU is producing large quantities of O_2_, which have to be sold for additional revenue. At the ARPA-e REFUEL aspirational values (FE = 90%, *j* = 0.3 A cm^−2^), Fig. S14e (ESI†) illustrates that NRR SOEL with hydrogen only becomes competitive with SMR Haber–Bosch at very optimistic electricity prices (≤$0.02 per kW h) and SOEL capital costs ($800 per m^2^). We estimated new aspirational values for the NRR SOEL with hydrogen oxidation using the same approach as discussed for aqueous NRR. By assuming a NRR SOEL and AEL CAPEX of $1209 per m^2^ and $564 per kW (base case assumptions in 2050) at an electricity price of $0.02 per kW h, the trend in Fig. S17 (ESI†) can be divided into three segments: FE > 90% at 0.4 A cm^−2^, FE > 85% at 0.5 A cm^−2^ and FE > 80% between 0.6–1 A cm^−2^. These performance requirements are significantly higher than aqueous NRR because improvements in the CAPEX and OPEX of the NRR SOEL unit have only a limited effect on the plant's economics.

NRR SOEL with water has even a smaller opportunity window, wherein electricity must decrease to unrealistic market prices (≤$0.018 per kW h) when assuming an electrolyzer CAPEX of $3000 per m^2^. Electricity prices up to $0.01 per kW h have been reported during peak periods of surplus renewable power.^72^ An electrochemical NH_3_ plant could theoretically operate along the volatile trend of low electricity market prices. The scale of the plant increases according to an assumed capacity factor, which results in higher investment costs. Wang *et al.* investigated the matter and observed an increase of the LCOA with $100 per tNH_3_ at a 0.2–0.3 capacity factor.^15^ Another issue is the compatibility with intermittent operation, which can especially be challenging for high temperature electrolysis, upstream and downstream units.

## Economic assessment of Li-mediated NRR

**Fig. 9:**
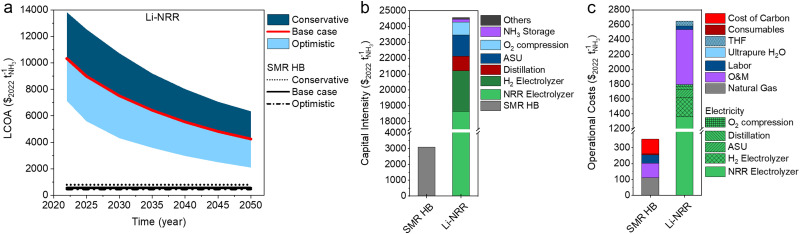
Economic analysis of Li-mediated NRR. (a) LCOA of Li-NRR. The black lines indicate the LCOA of SMR HB calculated with optimistic, base case and conservative price scenarios. (b) Capital intensity calculated with base case assumptions in 2050. (c) Operational costs calculated with the base case assumption in 2050. (a)–(c) The ARPA-e electrolyzer aspirational values (FE = 90%, *j* = 0.3 A cm^−2^) were used for the economic analysis.

Among the assessed sustainable NH_3_ pathways, Li-mediated NRR is the most expensive process and cannot become cost competitive with SMR Haber–Bosch even when considering the most optimistic cost factors (Fig. 9a). Due to the complexity of the electrolyzer system (hybrid flow cell, compatibility with organic electrolytes, moisture free operation) and significant power demand, the BoP will be excessive and comparable with other energy intensive electrochemical processes, such as chlor-alkali (∼$30 000 per m^2^).^63^ With a base case estimate of $18 650 per m^2^ (in 2050), approximately 75% of the capital intensity is directly related to the electrolyzer system (Fig. 9b). The operational costs in Fig. 9c show that the electricity consumption of the Li-NRR electrolyzer accounts for almost 50% of the total OPEX (∼$1360 per tNH_3_), mainly due to its low EE. By changing to a more compact cell design, a zero gap membrane electrode assembly without ohmic losses, the LCOA can be reduced by ∼50% (Fig. S18a, ESI†), but this is still not sufficient.

Another strategy is to find an alternative mediator with a significantly lower deposition potential. Although Ca has recently been identified as an active mediator besides Li,^42^ its reduction potential differs only +0.2 V *vs.* Li, which results in a limited gain in the OPEX. We decided to do a preliminary techno-economic screening, whereby Mg and Al are implemented as potential mediators (+0.7 V and +1.37 *vs.* Li). We assume a zero-gap electrolyzer configuration with the Li-plating activation overpotential and the same upstream and downstream units as used in the Li-NRR process. Under the base case assumptions (in 2050), Mg-NRR or Al-NRR allow a LCOA reduction of −$262 and −$547 per tNH_3_ with respect to Li-NRR in MEA configuration. These cost saving scenarios are insufficient and do not allow mediated NRR to compete with other sustainable ammonia processes (as illustrated in Fig. S18, ESI†). This incentivises the search for mediators beyond Al in order to enable mediated NRR as a compelling approach.

## Future outlook

**Fig. 10:**
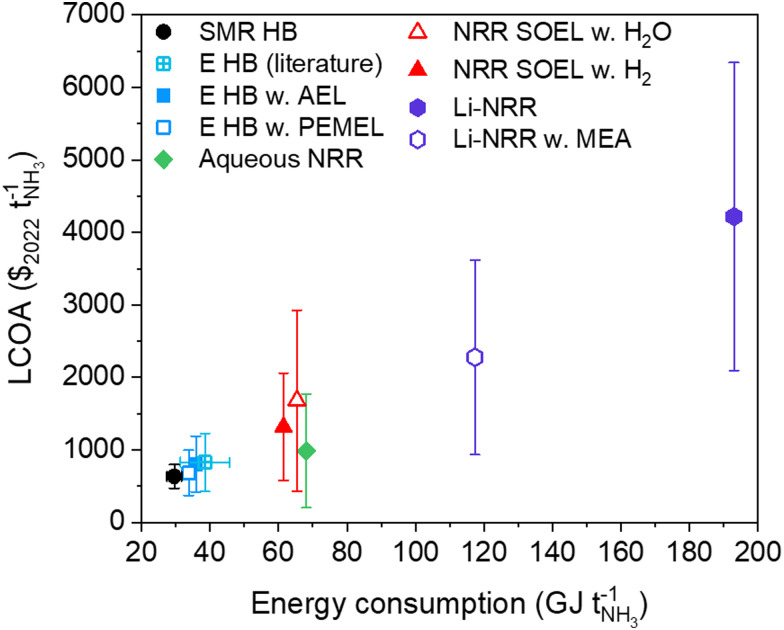
LCOA *versus* the energy consumption of the ammonia production routes discussed in this work. The *y*-error bars indicate the LCOA at optimistic and conservative cost scenarios in 2050 (from Table S17, ESI†). The data points represent the average between the optimistic and conservative cost assumptions, and not necessarily the base case assumptions. The *x*-error bar of SMR HB (black) represents the best available technology (27.4–31.8 GJ per tNH_3_). The variation among the reported literature values on the LCOA ($827 ± 398 per tNH_3_) and energy input (38.6 ± 7.1 GJ per tNH_3_) of electrified HB at a similar production capacity were taken from ref. 8 and 73–75, and added for comparison (sapphire).

SMR Haber–Bosch will be around for several decades until the technology can be phased out with a zero-emissions alternative. The transition rate towards green ammonia will mainly depend on the level of inducible carbon tax by governmental policies, future levelized cost of renewable electricity and reductions in the electrolyzer manufacturing costs. Among the options for sustainable ammonia synthesis at a small scale plant (91 tonnes per day), electrified Haber–Bosch remains the most promising technology in terms of maturity, costs and energy efficiency (see Fig. 10). Nonetheless, research exploration for alternative pathways must continue.

Aqueous NRR at ambient conditions is thermodynamically the most favorable approach, but the energy losses associated with activation overpotentials, ohmic losses, N_2_ feedstock production and distillation are often overlooked and decrease the energy efficiency of the process significantly. The low single-pass conversion and inability to recycle unreacted N_2_ demands an ASU with a large capacity, which will also produce vast quantities of O_2_. Selling O_2_ as a commodity is therefore essential to stimulate cash flow. Unfortunately, the current state of the aqueous NRR field is orders of magnitude away (*j* < 0.001 A cm^−2^, FE < 1%) from reaching our newly defined electrolyzer aspirational values (FE > 80% at 0.3 A cm^−2^, FE > 70% at 0.4 A cm^−2^ and FE > 65% between 0.5–1 A cm^−2^). Moreover, numerous publications that claim to have activated N_2_ are dubious and irreproducible,^76–79^ which can mostly be assigned to extraneous sources of NH_3_ or the electroreduction of NO_*x*_ species.^80^ It remains to be seen if aqueous NRR will ever be experimentally demonstrated unambiguously at the intended *j* and FE.

High temperature NRR combined with water oxidation is as challenging as aqueous NRR at room temperature, wherein reported *j* (<0.01 A cm^−2^) and FE (<1%) remain at a bare minimum.^81^ On the contrary, high temperature NRR with H_2_ oxidation allows N_2_ activation to be more selective (FE > 70%).^81^ Yet, both the FE and the current densities obtained at lab scale do not meet with the bare minimum *j* and FE (FE > 90% at 0.4 A cm^−2^), hence remain impractical for industrial applications. More progress has been made in the Li-NRR field, where current densities of 1 A cm^−2^ and FEs near unity were reported,^31,32^ continuous flow and membrane electrode assembly cells have been developed,^30,40,82^ and Ca has been identified as an active N_2_ mediator.^42^ These achievements have progressed the mediated NRR field tremendously, but due to the fundamentally low energy efficiencies of the electrochemical conversion step and the overall complexity of the process, ammonia production at a competitive cost price will be a major challenge for its future application.

## Conclusion

In this work, we designed detailed process models for the electrochemical production of NH_3_ to gain insights into the main bottlenecks of the process and to understand what process conditions are required to reach economic parity with SMR Haber–Bosch. Electrified Haber–Bosch with PEMEL is so far the most attractive process. However, current PEMEL investment costs and electricity prices need to be reduced to $615 per kW and $0.035 per kW h, which can be achieved within two decades according to future price projections. Aqueous NRR at ambient conditions needs even more optimistic scenarios and only becomes promising if the electricity price drops below $0.025 per kW h at $5600 per m^2^ (electrolyzer CAPEX). In addition to this, the NRR performance has to be increased to FE > 80% and *j* ≥ 0.3 A cm^−2^, a daunting task when comparing to the current state of the field. On the contrary, numerous experimental reports show that NRR in a SOEL with hydrogen oxidation is more selective (FE > 70%), but current densities remain at industrially irrelevant scales. Additionally, we find that SOEL based processes tend to be more capital intensive due to the additional requirement of heat exchangers and more auxiliary equipment. Hence, high temperature NRR is only cost competitive at the most optimistic and perhaps unrealistic economic scenario (≤$0.02 per kW h, ≤$800 per m^2^). Li-NRR has progressed tremendously over the last years in terms of scale, continuity, ammonia yield and selectivity. Unfortunately, the inherently low energy efficiency (<13%) of the electrolyzer causes disproportionally high operational costs. The EE can be improved by developing MEA-type electrolyzers to circumvent electrolyte conductivity losses or by implementing an alternative mediator with a more positive plating potential than Li, such as Mg or Al. For a small scale plant at 91 tonnes per day, Li-mediated NRR is under the most optimistic economic assumptions not economically feasible. This means that Li-NRR and also Ca-NRR remain interesting subjects for scientific research, but might never be integrated into a profitable application or process. Future research has to focus on the identification of mediators beyond Li and Ca. For now, electrified Haber–Bosch remains the only compelling electrolysis based pathway for sustainable ammonia production.

## Author contributions

B. I. and R. K. conceived the work. A. V. and D. V. D. S. developed initial versions of the process models and B. I. finalized them. M. R., M. P. F., A. S. K., W. D. J., F. M. M. and R. K. gave mentorship during the design phase of the process models. The economic analysis was performed by B. I. and A. V. with input from M. R. The manuscript was written by B. I. The project was supervised by R. K. All authors helped interpretating the results and contributed in the editing and revision process of the manuscript.

## Data availability

The data supporting this article have been included as part of the ESI.† Additional data is available on reasonable request at the authors.

## Conflicts of interest

There are no conflicts to declare.

## Supplementary Material

EE-017-D4EE03299C-s001
