# Supplementary material for: Techno-economic assessment of different small-scale electrochemical NH3 production plants
Source: Energy Environ Sci. 2024 Oct 3;17(21):7983–98. doi: 10.1039/d4ee03299c (PMC11462118; doi:10.1039/d4ee03299c)
Supplement: EE-017-D4EE03299C-s001 [file EE-017-D4EE03299C-s001.pdf]

Supplementary Information (SI) for Energy & Environmental Science.
This journal is © The Royal Society of Chemistry 2024

Electronic Supplementary Information (ESI)

Techno-economic Assessment of Different Small-scale Electrochemical NH_3 Production Plants

Boaz Izelaar [a], Mahinder Ramdin [a], Alexander Vlierboom [a], Mar Pérez-Fortes [b], Deanne van der Slikke [a], Asvin Sajeev Kumar [a], Wiebren de Jong [a], Fokko M. Mulder [c], Ruud Kortlever [a]*

[a] Process and Energy Department, Faculty of Mechanical Engineering, Delft University of Technology, 2628 CB Delft, The Netherlands

[b] Engineering, Systems and Services Department, Faculty of Technology, Policy and Management, Delft University of Technology, 2628 BX Delft, The Netherlands

[c] Chemical Engineering Department, Faculty of Applied Sciences, Delft University of Technology, 2629 HZ Delft, The Netherlands

*Corresponding Author; E-mail: R.Kortlever@tudelft.nl

Contents

S1 Process Flow Diagrams, Stream Summary and Equipment List S3
S1.1 Electrified Haber-Bosch S3
S1.2 Aqueous NRR at Ambient Conditions S7
S1.3 NRR SOEL with Water Oxidation S13
S1.4 NRR SOEL with Hydrogen Oxidation S16
S1.5 Li-mediated NRR S20
S2 Supplementary Figures S24
S3 Supplementary Tables S32
S4 Supplementary Methods S37
S4.1 NRR Electrolyzers S37
S4.1.1 Gibbs Free Energy and the Equilibrium Potential S37
S4.1.2 Activation Overpotentials and Ohmic Losses S38
S4.2 H_2 Electrolyzers S38
S4.3 Air Separation Unit S39
S4.4 Heat Exchangers, Compressors and Pumps S39
S4.5 Distillation S40
S4.6 Adsorption S42
S4.7 Storage Tanks S45
S4.8 Haber-Bosch Synthesis Loop S46
S4.9 Techno-economic Assumptions S47
Supplementary References S50

S1 Process Flow Diagrams, Stream Summary and Equipment List

S1.1 Electrified Haber-Bosch

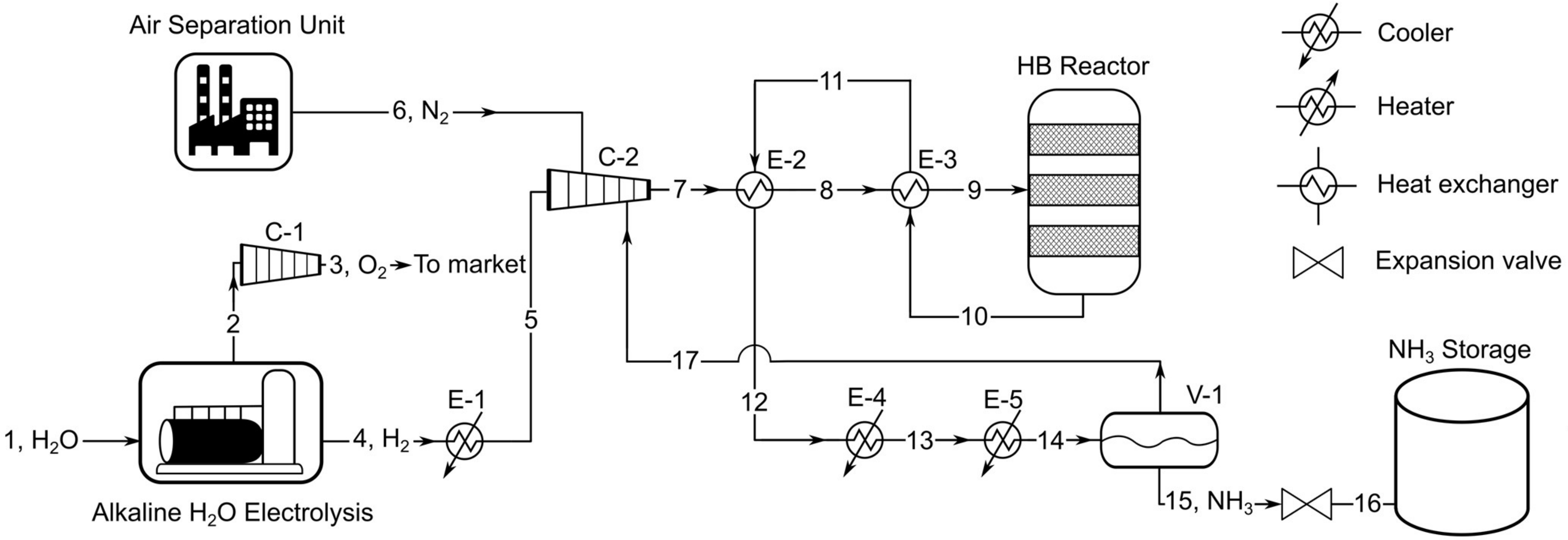

Figure S1. Process flow diagram of the electrified Haber-Bosch process with an alkaline water electrolyzer (AEL). The electrified Haber-Bosch with a proton exchange membrane electrolyzer (PEMEL) is identical, except that the feed-gas compressor (C-2) requires less compression stages.

Table S1. Stream summary of the electrified Haber-Bosch process with AEL (Figure S1).

**Property**	**1**	**2**	**3**	**4**	**5**	**6**	**7**
Temperature (°C)	25	80	40	80	40	25	40
Pressure (bar)	1.01	1.01	163	1.01	1.01	6	157
Mole flows (kmol h^{-1})	370.43	166.47	166.47	334.06	334.06	111.37	1830.5
NH_3 (kmol h^{-1})							54.835
H_2 (kmol h^{-1})				334.06	334.06		1331.75
N_2 (kmol h^{-1})						111.37	443.82
O_2 (kmol h^{-1})		166.47	166.47				
KOH (kmol h^{-1})							
H_2O (kmol h^{-1})	370.43						
Mole fractions							
NH_3							0.03
H_2				1	1		0.73
N_2						1	0.24
O_2		1	1				
KOH							
H_2O	1						
Mass flow (kg h^{-1})	6667.77	5326.69	5326.69	673.42	673.42	3119.75	16054.1
**Property**	**8**	**9**	**10**	**11**	**12**	**13**	**14**
Temperature (°C)	220	400	619	437	239	239	-5
Pressure (bar)	156	155	152	151	150	149	147
Mole flows (kmol h^{-1})	1830.5	1830.5	1608.54	1608.54	1608.54	1608.54	1608.54
NH_3 (kmol h^{-1})	54.835	54.835	276.79	276.79	276.79	276.79	276.79
H_2 (kmol h^{-1})	1331.75	1331.75	998.81	998.81	998.81	998.81	
N_2 (kmol h^{-1})	443.82	443.82	332.94	332.94	332.94	332.94	998.81
O_2 (kmol h^{-1})							332.94
KOH (kmol h^{-1})							
H_2O (kmol h^{-1})							
Mole fractions							
NH_3	0.03	0.03	0.17	0.17	0.17	0.17	0.17
H_2	0.73	0.73	0.62	0.62	0.62	0.62	0.62
N_2	0.24	0.24	0.21	0.21	0.21	0.21	0.21
O_2							
KOH							
H_2O							
Mass flow (kg h^{-1})	16054.1	16054.1	16054.1	16054.1	16054.1	16054.1	16054.1

Table S1 (continued). Stream summary of the electrified Haber-Bosch process with AEL (Figure S1).

**Property**	**15**	**16**	**17**
Temperature (°C)	-5	-34	-5
Pressure (bar)	145	1.01	145
Mole flows (kmol h^{-1})	223.25	223.25	1385.29
NH_3 (kmol h^{-1})	221.92	221.92	54.87
H_2 (kmol h^{-1})			
N_2 (kmol h^{-1})	0.34	0.34	997.83
O_2 (kmol h^{-1})	0.98	0.98	332.60
KOH (kmol h^{-1})			
H_2O (kmol h^{-1})			
Mole fractions			
NH_3	0.995	0.995	0.04
H_2	0.004	0.004	0.72
N_2	0.001	0.001	0.24
O_2			
KOH			
H_2O			
Mass flow (kg h^{-1})	3790.98	3790.98	12263.2

Table S2. Equipment list of the electrified Haber-Bosch process with AEL (Figure S1). CW: Cooling water

**Main Equipment**	**C-1**	**C-2**	**E-1**	**E-2**	**E-3**	**E-4**
Type	6-stage compressor	6-stage compressor	Cooler	Heat Exchanger	Heat Exchanger	Cooler
Power (kW)	2701	1137.92				
Heat (kW)	2234 (intercoolers)	1184.12	108.5	2923	2776	3062
Area (m^2)	347 (intercoolers)	314.72	104	53.02	50.96	87.8
U (kW m^{-2})	-	-	42.2	310.4	287.4	574.1
Medium	CW	CW	CW			CW

**Main Equipment**	**E-5**	**V-1**	**Air separation unit**
Type	Condensor	Flash drum	Pressure-swing adsorption
Power (kW)			1372.03
Heat (kW)	1746		
Area (m^2)	71.23		
U (kW m^{-2})	658.6		
Medium	Ammonia		

Table S3. Stream data modification when replacing AEL with PEMEL in the electrified HB process (referring to Figure S1).

**Property**	**2**	**4**	**5**
Temperature (°C)	80	80	40
Pressure (bar)	35	35	35
Mole flows (kmol h^{-1})	166.47	334.06	334.06
NH_3 (kmol h^{-1})			
H_2 (kmol h^{-1})		334.06	334.06
N_2 (kmol h^{-1})			
O_2 (kmol h^{-1})	166.47		
KOH (kmol h^{-1})			
H_2O (kmol h^{-1})			
Mole fractions			
NH_3			
H_2		1	1
N_2			
O_2	1		
KOH			
H_2O			
Mass flow (kg h^{-1})	5326.69	673.42	673.42

Table S4. Equipment data modifications when replacing AEL with PEMEL in the electrified HB process (referring to Figure S1).

**Main Equipment**	**C-1**	**C-2**	**E-1**
Type	2-stage compressor	4-stage compressor	Cooler
Power (kW)	333.2 kW	1203	
Heat (kW)	411.2 (intercoolers)	708 (intercoolers)	108
Area (m^2)	28.6 (intercoolers)	67.3 (intercoolers)	16.03
U (kW m^{-2})	-	-	276
Medium	CW	CW	CW

S1.2 Aqueous NRR at Ambient Conditions

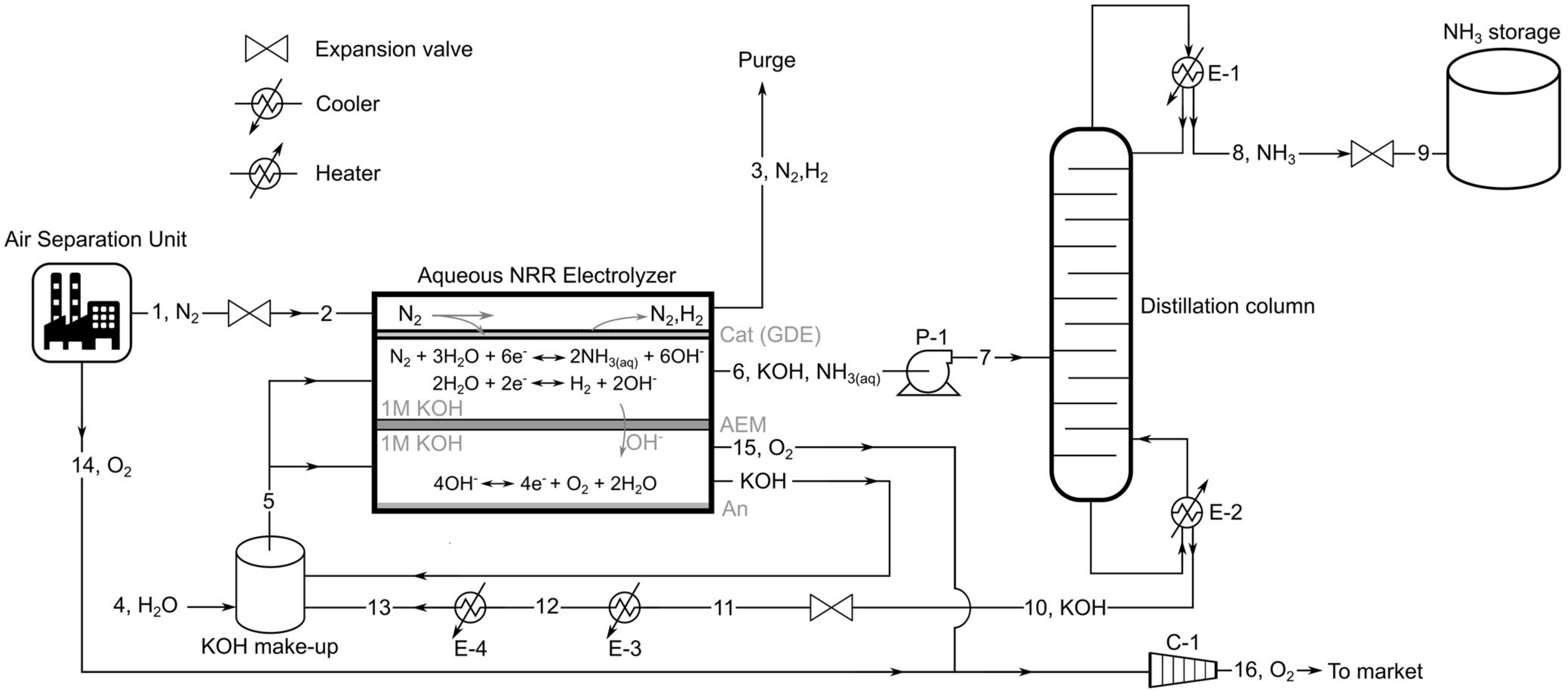

Figure S2. Process flow diagram of aqueous NRR at ambient conditions with a H_2 purge.

Table S5. Stream summary of the aqueous NRR process with a H_2 purge at a FE of 90% (Figure S2).

**Property**	**1**	**2**	**3**	**4**	**5**	**6**	**7**	**8**
Temperature (°C)	25	25	25	25	25	25	25	-24.8
Pressure (bar)	6	1.01	1.01	1.01	1.01	1.01	3.5	3.5
Mole flows (kmol h^{-1})	1110.92	1110.92	1035.82	666.0	2665.72	2221.80	2221.80	223.07
NH_3 (kmol h^{-1})					0.218	222.18	222.18	221.96
H_2 (kmol h^{-1})			36.99					
N_2 (kmol h^{-1})	1109.81	1109.81	998.83					
O_2 (kmol h^{-1})	1.11	1.11						
KOH (kmol h^{-1})					39.58	39.58	39.58	
H_2O (kmol h^{-1})				666.0	2625.916	1960.03	1960.03	1.11
Mole fractions								
NH_3					0.00008	0.1	0.1	0.995
H_2			0.036					
N_2	1	1	0.964					
O_2	1	1	0.964					
KOH					39.58	0.0178	0.0178	
H_2O				1	2625.92	0.882	0.882	0.005
Mass flow (kg h^{-1})	31121.24	31121.24	28055.1	12016.11	49531.36	41315.2	41315.2	3800.16
**Property**	**9**	**10**	**11**	**12**	**13**	**14**	**15**	**16**
Temperature (°C)	-33	140	101	40	25	25	25	40
Pressure (bar)	1.01	3.5	1.01	1.01	1.01	6	1	163
Mole flows (kmol h^{-1})	222.08	2038.21	2038.21	2038.21	2038.21	298.79	166.47	465.27
NH_3 (kmol h^{-1})	221.96	0.218	0.218	0.218	0.218			
H_2 (kmol h^{-1})								
N_2 (kmol h^{-1})								
O_2 (kmol h^{-1})						298.79	166.47	465.27
KOH (kmol h^{-1})		39.58	39.58	39.58	39.58			
H_2O (kmol h^{-1})	1.11	1958.92	1958.92	1958.92	1958.92			
Mole fractions								
NH_3	0.995	0.0001	0.0001	0.0001	0.0001			
H_2								
N_2								
O_2						1	1	1
KOH		0.0194	0.0194	0.0194	0.0194			
H_2O	0.005	0.961	0.961	0.961	0.961			
Mass flow (kg h^{-1})	3800.16	37515.1	37515.1	37515.1	37515.1	9561.11	5326.9	14888.02

Table S6. Equipment list of the aqueous NRR process with a H_2 purge at a FE of 90% (Figure S2).

**Main Equipment**	**P-1**	**E-1 (Distillation)**	**E-2 (Distillation)**	**E-3**	**E-4**	**C-1**	**Air separation Unit**
Type	Pump	Condenser	Reboiler	Cooler	Cooler	6-stage compressor	Cryogenic Distillation
Power (kW)	3.489					2393.4	11490.66
Heat (kW)		-806.8	6967.4	-4062	-602.5	-2345.9 (intercoolers)	
Area (m^2)		71.93	44.69	43.65	10.32	498.7 (intercoolers)	
U (kW m^{-2})		497.7	5230.8	2139.9	1031.1		
Medium		Propylene	Steam	CW	Ammonia	CW	

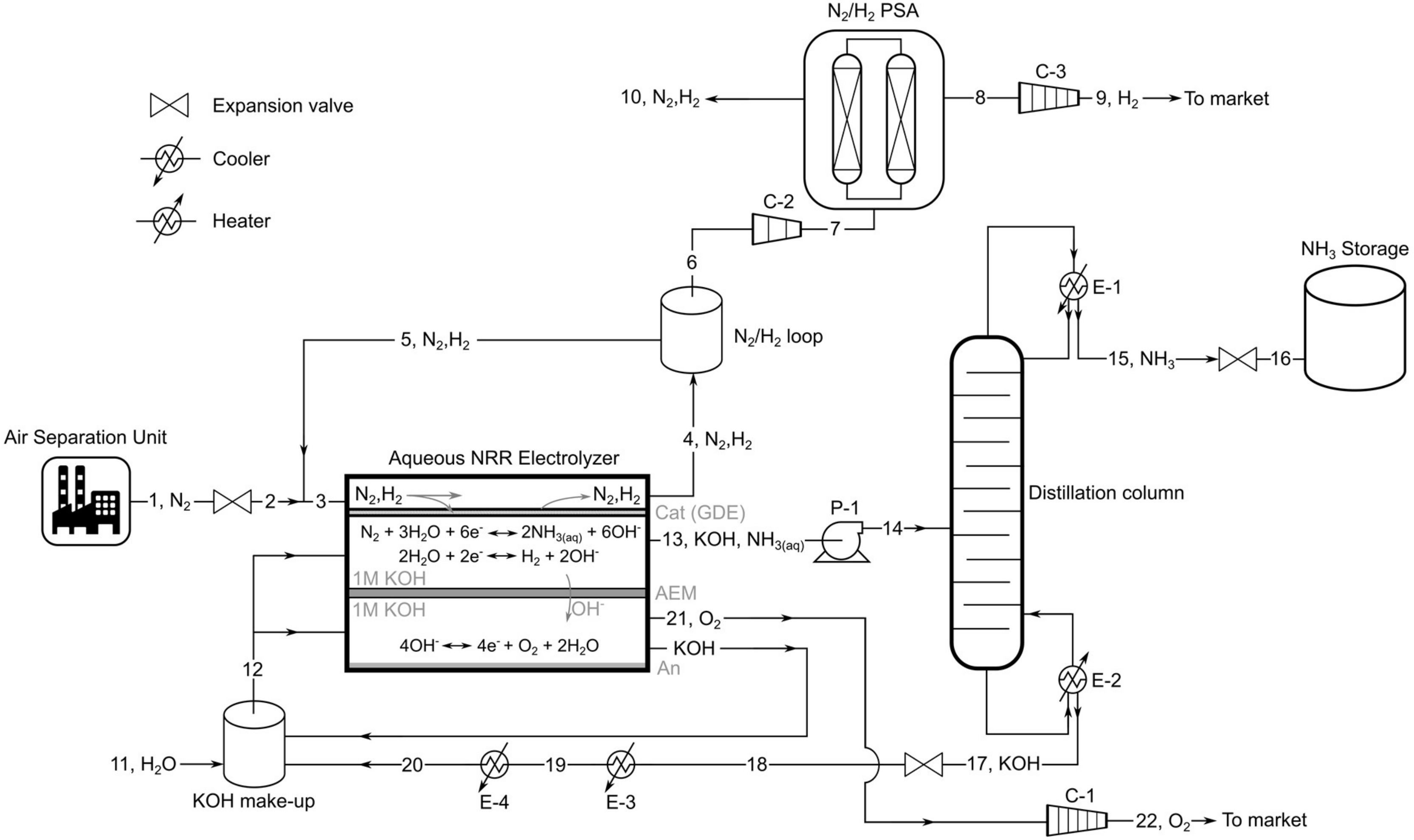

Figure S3. Process flow diagram of the aqueous NRR process at ambient conditions including a N_2/H_2 PSA separation step, N_2 recycle stream and storage vessel.

Table S7. Stream summary of the aqueous NRR process at a FE of 90% with a N_2/H_2 PSA (Figure S3).

**Property**	**1**	**2**	**3**	**4**	**5**	**6**	**7**
Temperature (°C)	25	25	25	25	25	25	40
Pressure (bar)	6	1.01	1.01	1.01	1.01	1.01	7
Mole flows (kmol h^{-1})	135.64	135.64	2570.97	2495.98	2435.33	61.654	61.654
NH_3 (kmol h^{-1})							
H_2 (kmol h^{-1})			1461.2	1498.19	1461.20	36.99	36.99
N_2 (kmol h^{-1})	135.64	135.64	1109.77	998.79	974.13	24.66	24.66
O_2 (kmol h^{-1})							
KOH (kmol h^{-1})							
H_2O (kmol h^{-1})							
Mole fractions							
NH_3							
H_2	1	1	0.57	0.6	0.6	0.6	0.6
N_2			0.43	0.4	0.4	0.4	0.4
O_2							
KOH							
H_2O							
Mass flow (kg h^{-1})	3799.8	3799.8	34034.29	30999.92	30234.49	765.43	765.43
**Property**	**8**	**9**	**10**	**11**	**12**	**13**	**14**
Temperature (°C)	40	40	40	25	25	25	25
Pressure (bar)	6	350	6	1.01	1.01	1.01	3.5
Mole flows (kmol h^{-1})	18.64	18.64	43.02	666.0	2665.72	2221.80	2221.80
NH_3 (kmol h^{-1})					0.218	222.18	222.18
H_2 (kmol h^{-1})	18.50	18.50	18.50				
N_2 (kmol h^{-1})	0.14	0.14	24.52				
O_2 (kmol h^{-1})							
KOH (kmol h^{-1})					39.58	39.58	39.58
H_2O (kmol h^{-1})				666.0	2625.916	1960.03	1960.03
Mole fractions							
NH_3					0.00008	0.1	0.1
H_2	0.9925	0.9925					
N_2	0.0075	0.0075					
O_2							
KOH					39.58	0.0178	0.0178
H_2O				1	2625.92	0.882	0.882
Mass flow (kg h^{-1})	41.2	41.2	724.23	12016.11	49531.36	41315.2	41315.2

Table S7 (continued). Stream summary of the aqueous NRR process at a FE of 90% with a N_2/H_2 PSA (Figure S3).

**Property**	**15**	**16**	**17**	**18**	**19**	**20**	**21**	**22**
Temperature (°C)	-24.8	-33	140	101	40	25	25	40
Pressure (bar)	3.5	1.01	3.5	1.01	1.01	1.01	1	163
Mole flows (kmol h^{-1})	223.07	222.08	2038.21	2038.21	2038.21	2038.21	166.47	166.47
NH_3 (kmol h^{-1})	221.96	221.96	0.218	0.218	0.218	0.218		
H_2 (kmol h^{-1})								
N_2 (kmol h^{-1})								
O_2 (kmol h^{-1})							166.47	166.47
KOH (kmol h^{-1})			39.58	39.58	39.58	39.58		
H_2O (kmol h^{-1})	1.11	1.11	1958.92	1958.92	1958.92	1958.92		
Mole fractions								
NH_3	0.995	0.995	0.0001	0.0001	0.0001	0.0001		
H_2								
N_2								
O_2							1	1
KOH			0.0194	0.0194	0.0194	0.0194		
H_2O	0.005	0.005	0.961	0.961	0.961	0.961		
Mass flow (kg h^{-1})	3800.16	3800.16	37515.1	37515.1	37515.1	37515.1	5326.9	5326.9

Table S8. Equipment list of the aqueous NRR process at a FE of 90% with a N_2/H_2 PSA (Figure S3).

**Main Equipment**	**P-1**	**E-1 (Distillation)**	**E-2 (Distillation)**	**E-3**	**E-4**	**C-1**	**C-2**
Type	Pump	Condenser	Reboiler	Cooler	Cooler	6-stage compressor	3-stage compressor
Power (kW)	3.489					1093.14	169.48
Heat (kW)		-806.8	6967.4	-4062	-602.5	-1063.21 (intercoolers)	-155.45 (intercooler)
Area (m^2)		71.93	44.69	43.65	10.32	301.03 (intercooler)	43.73 (intercooler)
U (kW m^{-2})		497.7	5230.8	2139.9	1031.1		
Medium		Propylene	Steam	CW	Ammonia	CW	CW

**Main Equipment**	**C-3**	**Air separation Unit**
Type	6-stage compressor	Cryogenic Distillation
Power (kW)	93.37	1671.91
Heat (kW)	86.37 (intercooler)	
Area (m^2)	4.74 (intercooler)	
U (kW m^{-2})		
Medium	CW	

S1.3 NRR SOEL with Water Oxidation

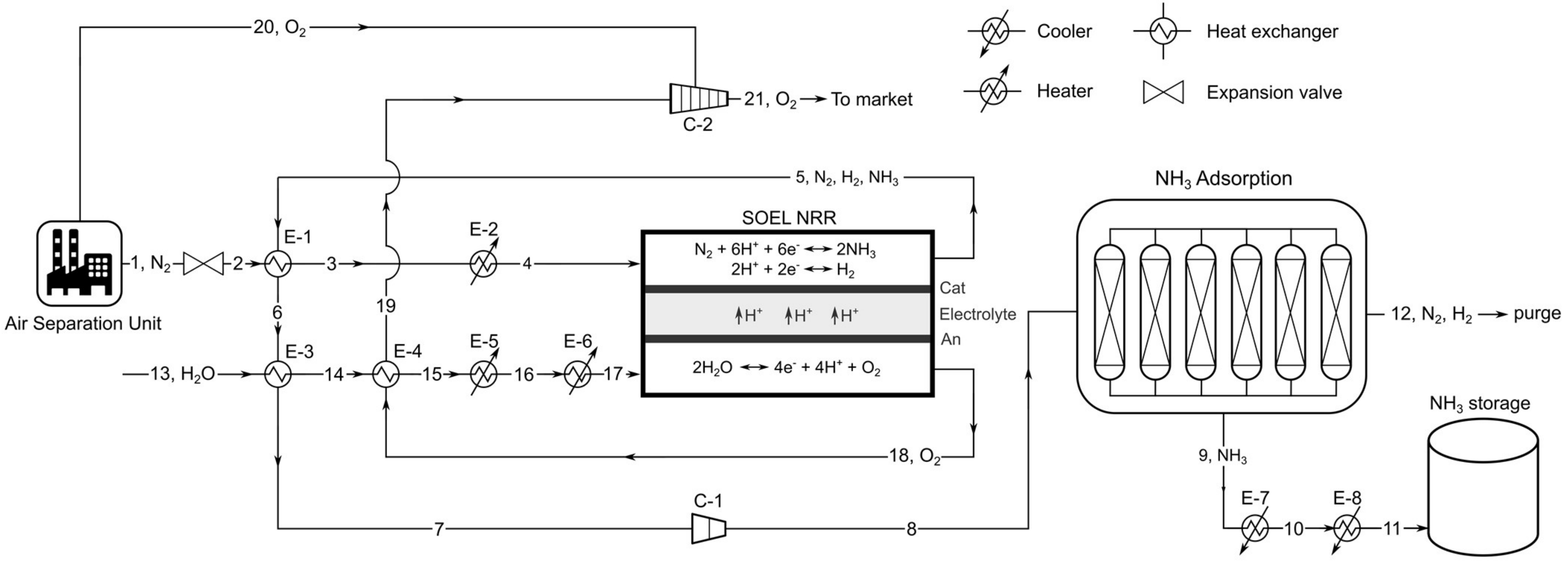

Figure S4. Process flow diagram of the NRR SOEL process with water oxidation.

Table S9. Stream summary of the NRR SOEL process with water oxidation process at a FE of 90% (Figure S4).

**Property**	**1**	**2**	**3**	**4**	**5**	**6**	**7**
Temperature (°C)	25	25	540	550	550	167	40
Pressure (bar)	6	1.01	1.01	1.01	1.01	1.01	1.01
Mole flows (kmol h^{-1})	1233.1	1233.1	1233.1	1233.1	1397.51	1397.51	1397.51
NH_3 (kmol h^{-1})					246.62	246.62	246.62
H_2 (kmol h^{-1})					41.1	41.1	41.1
N_2 (kmol h^{-1})	1233.1	1233.1	1233.1	1233.1	1109.78	1109.78	1109.78
O_2 (kmol h^{-1})							
H_2O (kmol h^{-1})							
Mole fractions							
NH_3					0.176	0.176	0.176
H_2					0.029	0.029	0.029
N_2	1	1	1	1	0.794	0.794	0.794
O_2							
H_2O							
Mass flow (kg h^{-1})	34543.22	34543.22	34543.22	34543.22	35371.93	35371.93	35371.93
**Property**	**8**	**9**	**10**	**11**	**12**	**13**	**14**
Temperature (°C)	40	200	40	-33	200	25	98
Pressure (bar)	3.5	1.01	1.01	1.01	3.5	1.01	1.01
Mole flows (kmol h^{-1})	1397.51	223.07	223.07	223.07	1174.44	986.48	986.48
NH_3 (kmol h^{-1})	246.62	221.96	221.96	221.96	24.66		
H_2 (kmol h^{-1})	41.1	0.22	0.22	0.22	40.88		
N_2 (kmol h^{-1})	1109.78	0.89	0.89	0.89	1108.89		
O_2 (kmol h^{-1})							
H_2O (kmol h^{-1})						986.48	986.48
Mole fractions							
NH_3	0.176	0.995	0.995	0.995	0.021		
H_2	0.029	0.004	0.004	0.004	0.035		
N_2	0.794	0.001	0.001	0.001	0.94		
O_2							
H_2O						1	1
Mass flow (kg h^{-1})	35371.93	3805.6	3805.6	3805.6	31566.33	17771.66	17771.66

Table S9 (Continued). Stream summary of the NRR SOEL process with water oxidation process at a FE of 90% (Figure S4).

**Property**	**15**	**16**	**17**	**18**	**19**	**20**	**21**
Temperature (°C)	102	164	550	550	110	25	40
Pressure (bar)	1.01	1.01	1.01	1.01	1.01	6	163
Mole flows (kmol h^{-1})	986.48	986.48	986.48	184.96	184.96	331.99	516.95
NH_3 (kmol h^{-1})							
H_2 (kmol h^{-1})							
N_2 (kmol h^{-1})							
O_2 (kmol h^{-1})				184.96	184.96	331.99	516.95
H_2O (kmol h^{-1})	986.48	986.48	986.48				
Mole fractions							
NH_3							
H_2							
N_2							
O_2				1	1	1	1
H_2O	1	1	1				
Mass flow (kg h^{-1})	17771.66	17771.66	17771.66	5918.68	5918.68	10623.2	16531.9

Table S10. Equipment list of the NRR SOEL process with water oxidation process at a FE of 90% (Figure S4).

**Main Equipment**	**E-1**	**E-2**	**E-3**	**E-4**	**E-5**	**E-6**	**E-7**
Type	Heat Exchange r	Heat Exchanger	Heat Exchanger	Heat Exchanger	Heater	Heater	Cooler
Power (kW)							
Heat (kW)	5298	1635	724.6	108.1	11505	3894	-383.9
Area (m^2)	25000	2367	226.9	91.84	2865	1978	293.5
U (kW m^{-2})	5	22.9	18.6	6.2	51	5.3	21.5
Medium					MP Steam	Fired heat	CW
**Main Equipment**	**E-8**	**C-1**	**C-2**	**Air separation Unit**	**Adsorptio n**		
Type	Cooler	2-stage compressor	6-stage compressor	Cryogenic Distillation	6-zeolite adsorbers		
Power (kW)		2898	2713	12767	2927		
Heat (kW)	-159.9	-2748 (intercoolers)	-2788.3 (intercooler)				
Area (m^2)	266.7	1537 (intercooler)	586.23 (intercooler)				
U (kW m^{-2})	20.5						
Medium	Propylene	CW	CW				

S1.4 NRR SOEL with Hydrogen Oxidation

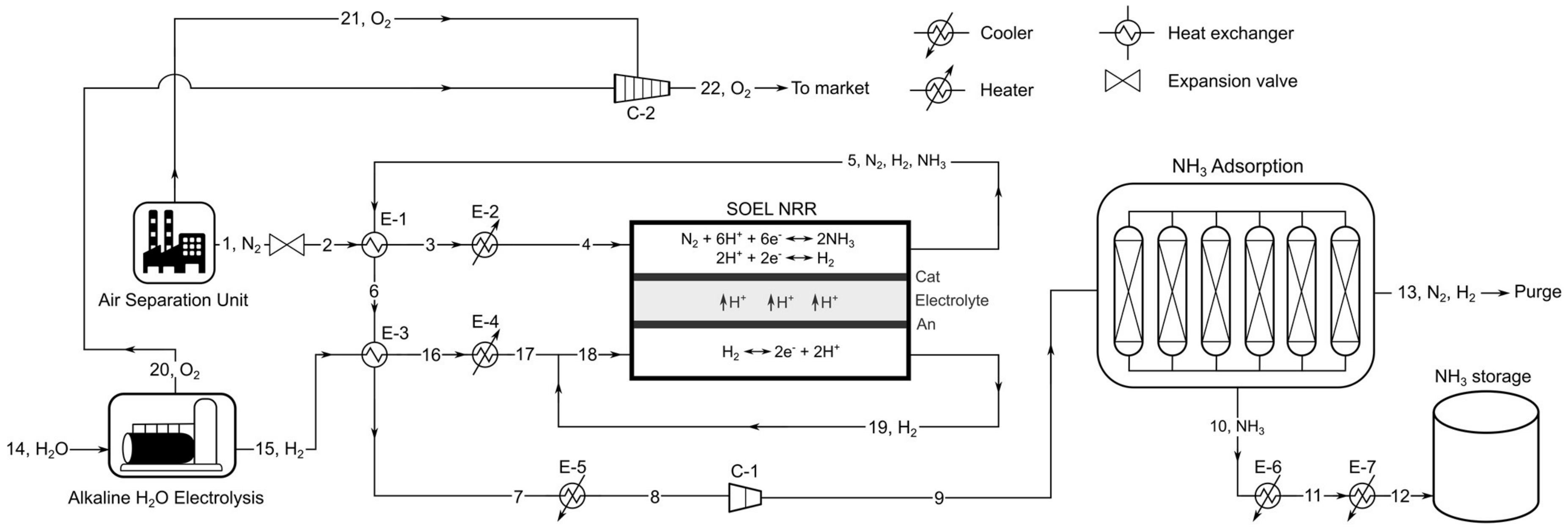

Figure S5. Process flow diagram of the NRR SOEL process with hydrogen oxidation including an additional alkaline water electrolyzer.

Property	1	2	3	4	5	6	7
Temperature (°C)	25	25	540	550	550	167	150
Pressure (bar)	6	1.01	1.01	1.01	1.01	1.01	1.01
Mole flows (kmol h^{-1})	1233.1	1233.1	1233.1	1233.1	1397.51	1397.51	1397.51
NH_3 (kmol h^{-1})					246.62	246.62	246.62
H_2 (kmol h^{-1})					41.1	41.1	41.1
N_2 (kmol h^{-1})	1233.1	1233.1	1233.1	1233.1	1109.78	1109.78	1109.78
O_2 (kmol h^{-1})							
H_2O (kmol h^{-1})							
Mole fractions							
NH_3					0.176	0.176	0.176
H_2					0.029	0.029	0.029
N_2	1	1	1	1	0.794	0.794	0.794
O_2							
H_2O							
Mass flow (kg h^{-1})	34543.22	34543.22	34543.22	34543.22	35371.93	35371.93	35371.93
**Property**	**8**	**9**	**10**	**11**	**12**	**13**	**14**
Temperature (°C)	40	40	200	40	-33	200	25
Pressure (bar)	1.01	3.5	1.01	1.01	1.01	3.5	1.01
Mole flows (kmol h^{-1})	1397.51	1397.51	223.07	223.07	223.07	1174.44	409.87
NH_3 (kmol h^{-1})	246.62	246.62	221.96	221.96	221.96	24.66	
H_2 (kmol h^{-1})	41.1	41.1	0.22	0.22	0.22	40.88	
N_2 (kmol h^{-1})	1109.78	1109.78	0.89	0.89	0.89	1108.89	
O_2 (kmol h^{-1})							
H_2O (kmol h^{-1})							409.87
Mole fractions							
NH_3	0.176	0.176	0.995	0.995	0.995	0.021	
H_2	0.029	0.029	0.004	0.004	0.004	0.035	
N_2	0.794	0.794	0.001	0.001	0.001	0.94	
O_2							
H_2O							1
Mass flow (kg h^{-1})	35371.93	35371.93	3805.6	3805.6	3805.6	31566.33	7442.85

Table S11. Stream summary of the NRR SOEL process with hydrogen oxidation at a FE of 90% (Figure S5).

Table S11 (Continued). Stream summary of the NRR SOEL process with hydrogen oxidation at a FE of 90% (Figure S5).

**Property**	**15**	**16**	**17**	**18**	**19**
Temperature (°C)	80	152	550	550	550
Pressure (bar)	1.01	1.01	1.01	1.01	1.01
Mole flows (kmol h^{-1})	369.93	369.93	369.93	3699.29	3329.36
NH_3 (kmol h^{-1})					
H_2 (kmol h^{-1})	369.93	369.93	369.93	3699.29	3329.36
N_2 (kmol h^{-1})					
O_2 (kmol h^{-1})					
H_2O (kmol h^{-1})					
Mole fractions					
NH_3					
H_2	1	1	1	1	
N_2					
O_2					1
H_2O					
Mass flow (kg h^{-1})	745.78	745.78	745.78	7457.77	6711.99

**Property**	**20**	**21**	**22**
Temperature (°C)	80	25	40
Pressure (bar)	1.01	6	163
Mole flows (kmol h^{-1})	184.96	331.99	516.95
NH_3 (kmol h^{-1})			
H_2 (kmol h^{-1})			
N_2 (kmol h^{-1})			
O_2 (kmol h^{-1})	184.96	331.99	516.95
H_2O (kmol h^{-1})			
Mole fractions			
NH_3			
H_2			
N_2			
O_2	1	1	1
H_2O			
Mass flow (kg h^{-1})	5918.7	10623.3	16541.9

Table S12. Equipment list of the NRR SOEL process with hydrogen oxidation at a FE of 90% (Figure S5).

**Main Equipment**	**E-1**	**E-2**	**E-3**	**E-4**	**E-5**	**E-6**	**E-7**
Type	Heat Exchanger	Heat Exchanger	Heater	Heater	Cooler	Cooler	Cooler
Power (kW)							
Heat (kW)	5298	216.9	108.1	1200	1418	-383.9	-159.9
Area (m^2)	25000	1131	91.84	389.8	1350	293.5	266.7
U (kW m^{-2})	5	6.3	6.2	8.1	22.9	21.5	20.5
Medium			Fired heat	Fired heat	CW	CW	Propylene

**Main Equipment**	**C-1**	**C-2**	**Air separation Unit**	**Adsorption**
Type	2-stage compressor	6-stage compressor	Cryogenic Distillation	6-zeolite adsorbers
Power (kW)	2883	2713	12767.17	2927.822
Heat (kW)	-2748 (intercoolers)	-2788.3 (intercooler)		
Area (m^2)	1537 (intercooler)	586.23 (intercooler)		
U (kW m^{-2})				
Medium	CW	CW		

S1.5 Li-mediated NRR

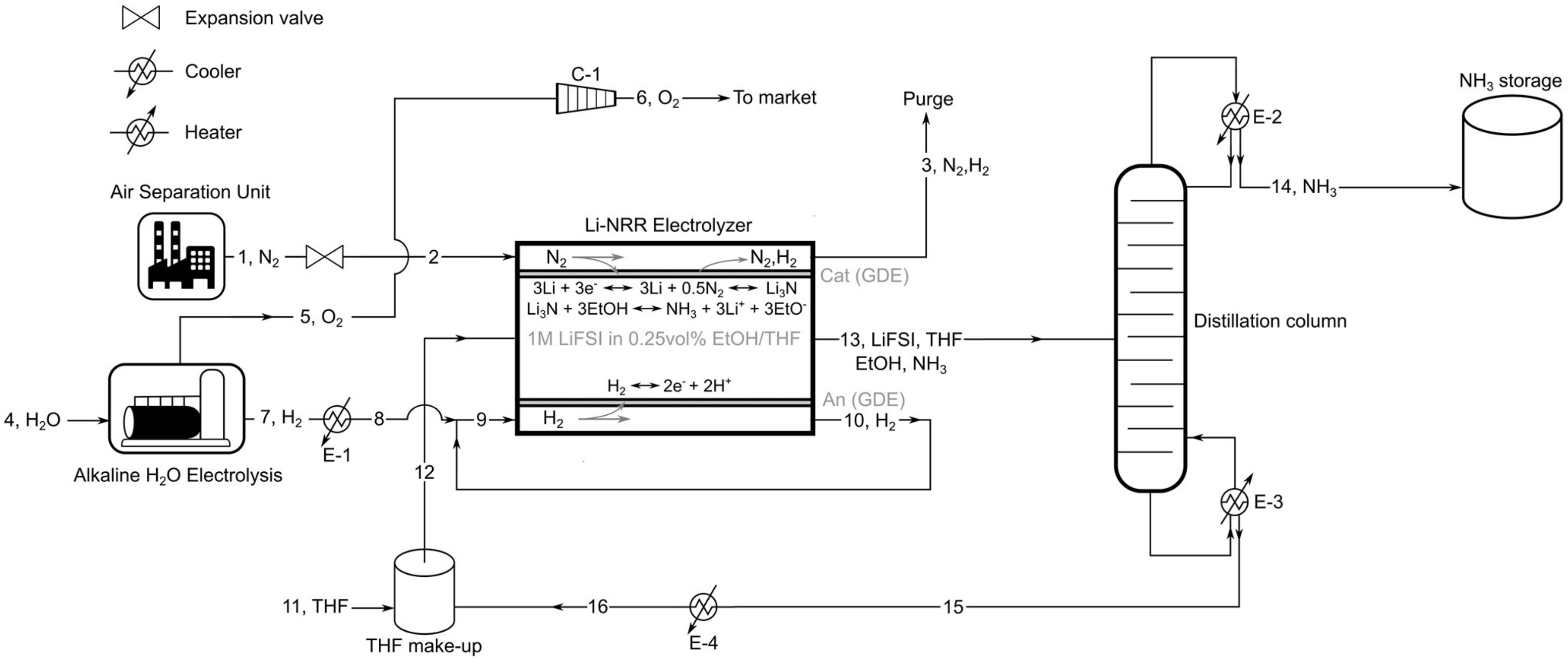

Figure S6. Process flow diagram of the Li-mediated NRR process with hydrogen oxidation and an additional alkaline water electrolyzer.

Table S13. Stream summary of the Li-mediated NRR process at a FE of 90% (Figure S6).

**Property**	**1**	**2**	**3**	**4**	**5**	**6**	**7**
Temperature (°C)	25	25	25	25	80	40	80
Pressure (bar)	6	1.01	1.01	1.01	1.01	163	1.01
Mole flows (kmol h^{-1})	1109.78	1109.78	1035.80	371.83	166.47	166.47	332.94
NH_3 (kmol h^{-1})							
H_2 (kmol h^{-1})			36.99				332.94
N_2 (kmol h^{-1})	1109.78	1109.78	988.8				
O_2 (kmol h^{-1})					166.47	166.47	
THF (kmol h^{-1})							
LiFSI (kmol h^{-1})							
EtOH (kmol h^{-1})							
H_2O (kmol h^{-1})				371.83			
Mole fractions							
NH_3							
H_2			0.0357				1
N_2	1	1	0.964				
O_2					1	1	
THF							
LiFSI							
EtOH							
H_2O				1			
Mass flow (kg h^{-1})	31088.9	31088.9	28054.6	6711.99	5326.81	5326.81	671.20
**Property**	**8**	**9**	**10**	**11**	**12**	**13**	**14**
Temperature (°C)	25	25	25	25	25	25	-34
Pressure (bar)	1.01	1.01	1.01	1.01	1.01	1.01	1.01
Mole flows (kmol h^{-1})	332.94	3329.4	2996.42	1.0921	2009.77	2230.73	223.07
NH_3 (kmol h^{-1})					1.12	223.07	221.96
H_2 (kmol h^{-1})	332.94	3329.4	2996.4				
N_2 (kmol h^{-1})							
O_2 (kmol h^{-1})							
THF (kmol h^{-1})				1.0921	1849.58	1849.577	1.12
LiFSI (kmol h^{-1})					152.56	151.56	
EtOH (kmol h^{-1})					6.52	6.517	
H_2O (kmol h^{-1})							
Mole fractions							
NH_3					0.0006	0.1	0.995
H_2	1	1	1				
N_2							
O_2							
THF				1	0.92	0.829	0.005
LiFSI					0.076	0.0679	
EtOH					0.0032	0.0029	
H_2O							
Mass flow (kg h^{-1})	671.20	6711.0	6040.79	78.75	162230.0	165823	3860.36

Table S13 (Continued). Stream summary of the Li-mediated NRR process at a FE of 90% (Figure S6).

**Property**	**15**	**16**
Temperature (°C)	50	25
Pressure (bar)	1.01	1.01
Mole flows (kmol h^{-1})	2008.677	2008.677
NH_3 (kmol h^{-1})	1.12	1.12
H_2 (kmol h^{-1})		
N_2 (kmol h^{-1})		
O_2 (kmol h^{-1})		
THF (kmol h^{-1})	1848.49	1848.49
LiFSI (kmol h^{-1})	151.56	151.56
EtOH (kmol h^{-1})	6.517	6.517
H_2O (kmol h^{-1})		
Mole fractions		
NH_3	0.0006	0.0006
H_2		
N_2		
O_2		
THF	0.92	0.92
LiFSI	0.076	0.076
EtOH	0.0032	0.0032
H_2O		
Mass flow (kg h^{-1})	162151.3	162151.3

Table S14. Equipment list of the Li-mediated NRR process at a FE of 90% (Figure S6).

**Main Equipment**	**E-1**	**E-2**	**E-3**	**E-4**	**C-1**	**Air separation Unit**
Type	Cooler	Condenser	Reboiler	Cooler	6-stage compressor	Cryogenic Distillation
Power (kW)					2440.75	11484.96
Heat (kW)	145.2	-2963.82	4116.39	1881	-2502.52 (intercooler)	
Area (m^2)	45.59	279.8	286.1	49.44	524.1 (intercooler)	
U ($kW\ m^{-2}$)	43.3	931.9	193.5	622.4		
Medium	Ammonia	Propylene	Electric heating	Propylene	CW	

S2 Supplementary Figures

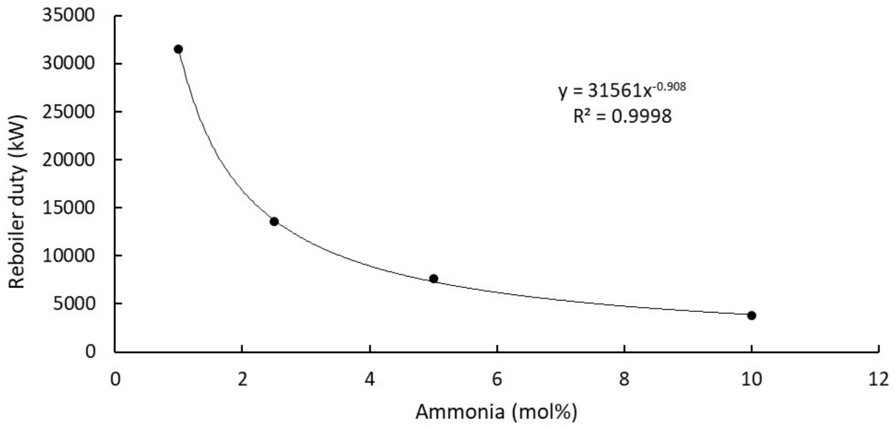

Figure S7. Reboiler duty as a function of the NH_3 mol fraction in the feed.

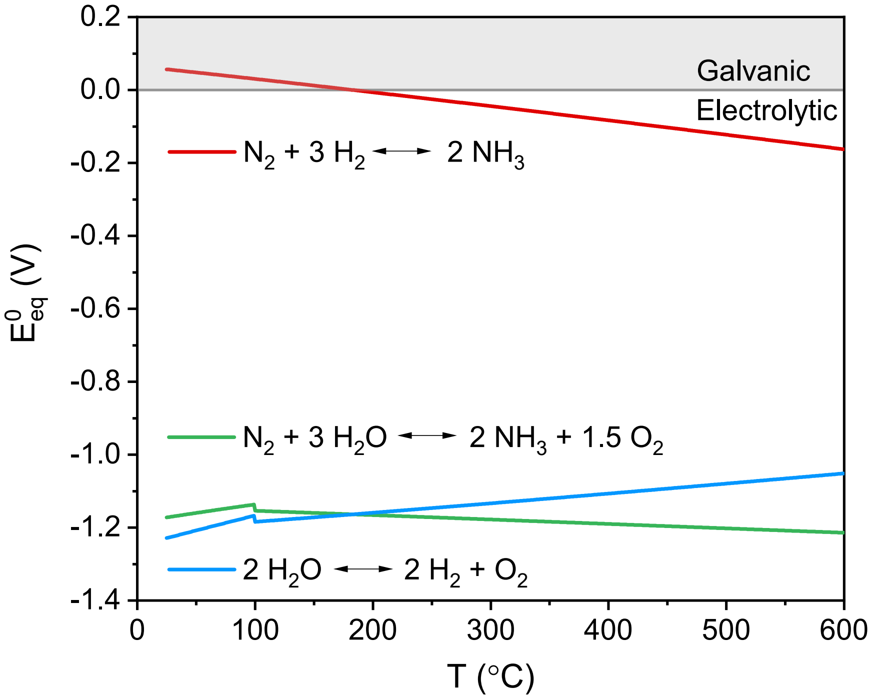

Figure S8. Standard equilibrium potentials for water electrolysis (blue), aqueous NRR (green) and indirect NRR (red) based on the Gibbs free energy.

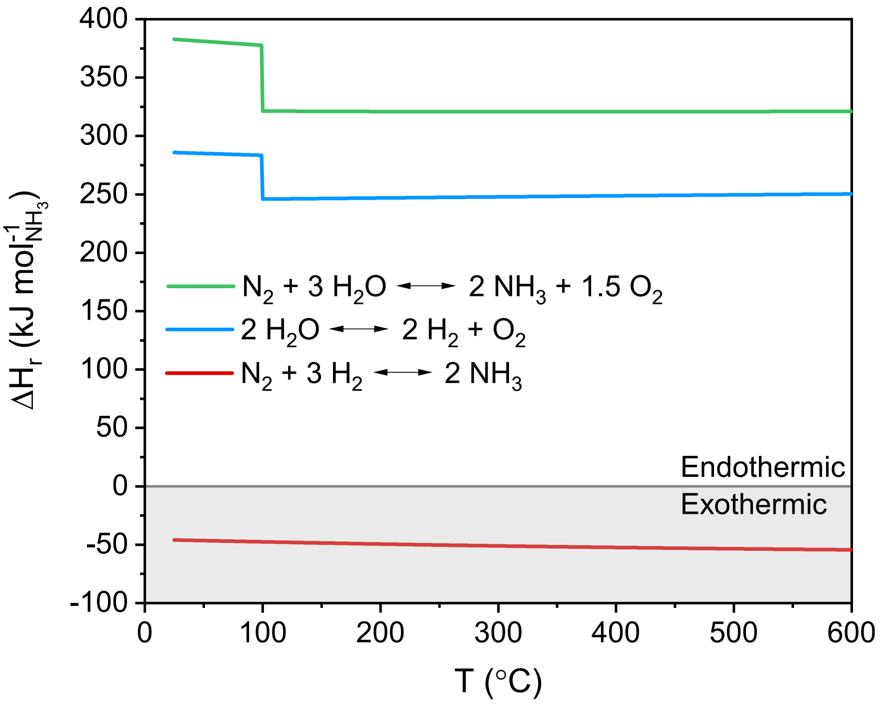

Figure S9. Reaction enthalpy diagram for water electrolysis (blue), aqueous NRR (green) and NRR with H_2 oxidation (red).

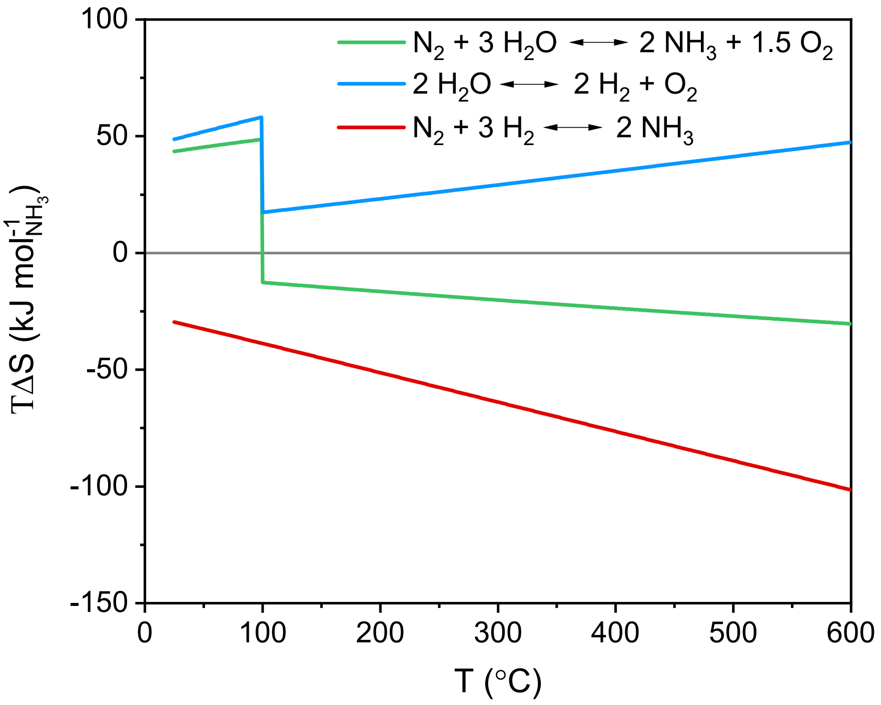

Figure S10. Reaction entropy diagram for water electrolysis (blue), aqueous NRR (green) and NRR with H_2 oxidation (red).

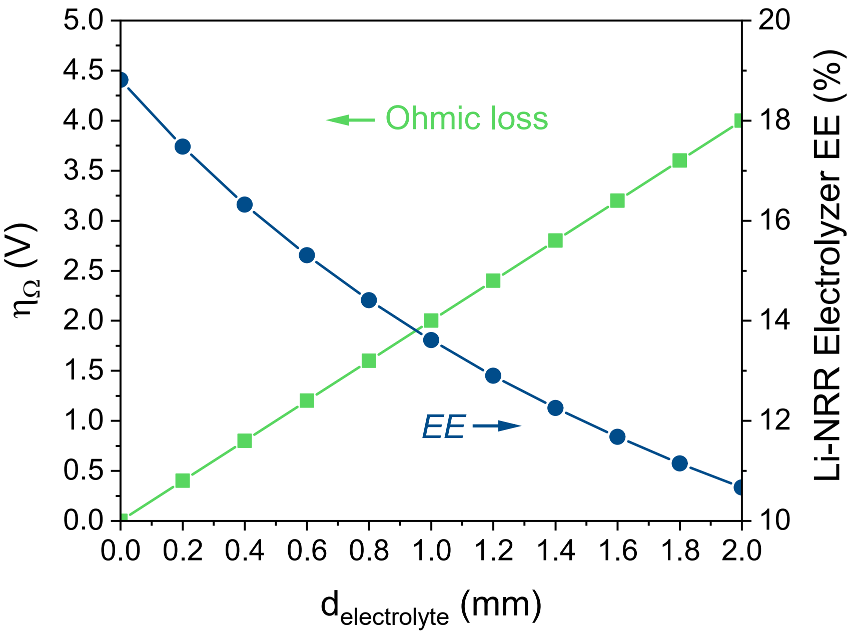

Figure S11. The Li-NRR electrolyzer energy efficiency at the aspirational values (0.3 A cm^{-2} and FE = 90%) including the energy input for H_2 production from water electrolysis.

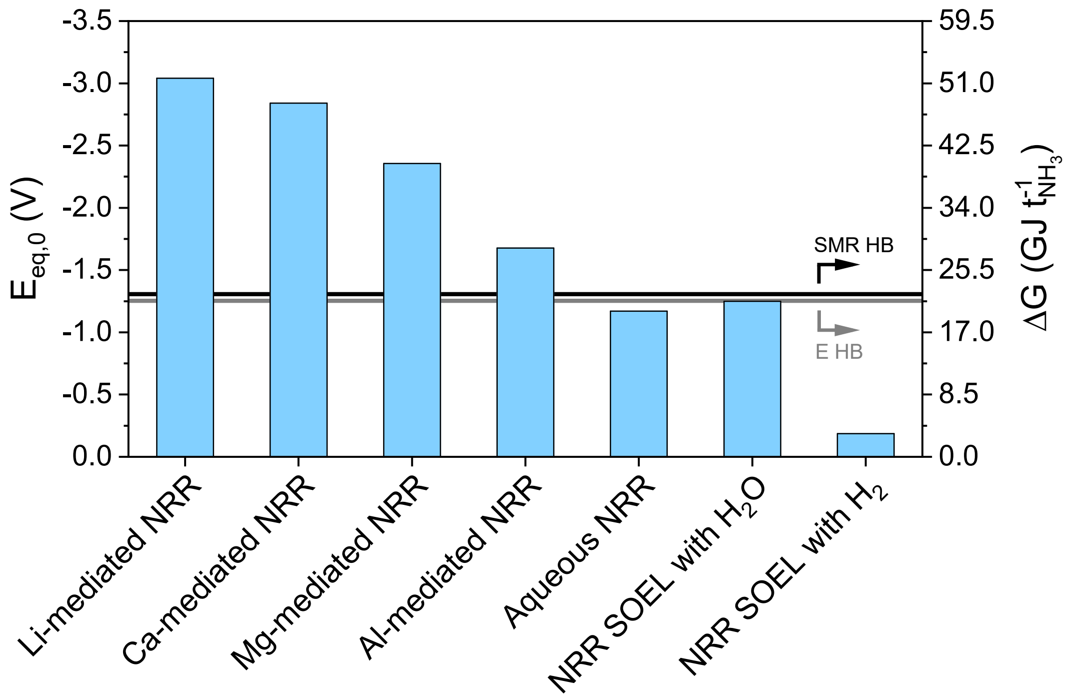

Figure S12. Standard equilibrium potentials and the Gibbs free energies for mediated NRR and other NRR electrolyzers. Additional energy input for H_2 production via water electrolysis is not included for indirect NRR electrolyzers. The thermodynamic minimum of electrified and SMR Haber-Bosch are also added for referencing. Standard equilibrium potentials for Li, Ca, Mg and Al plating are obtained from Bard & Faulkner.[1]

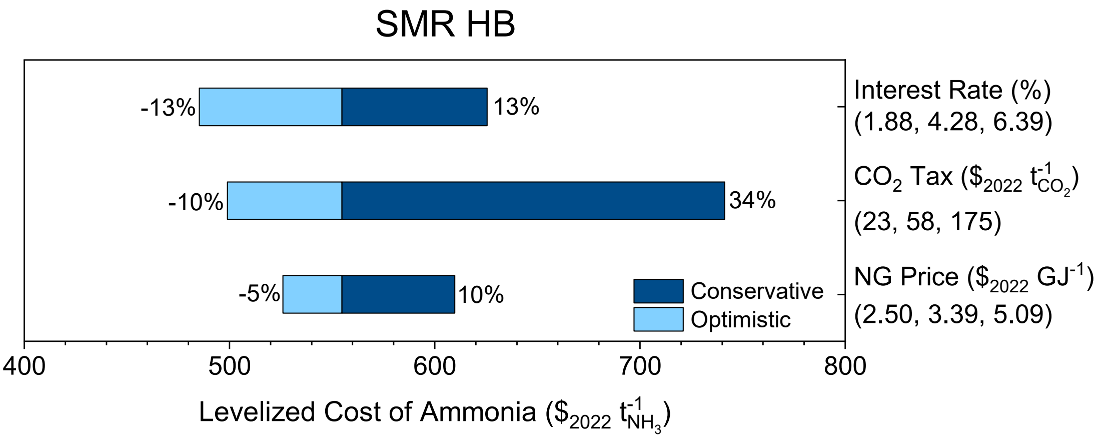

Figure S13. Sensitivity analysis of a small scale (91 tNH_3 per day) SMR HB plant.

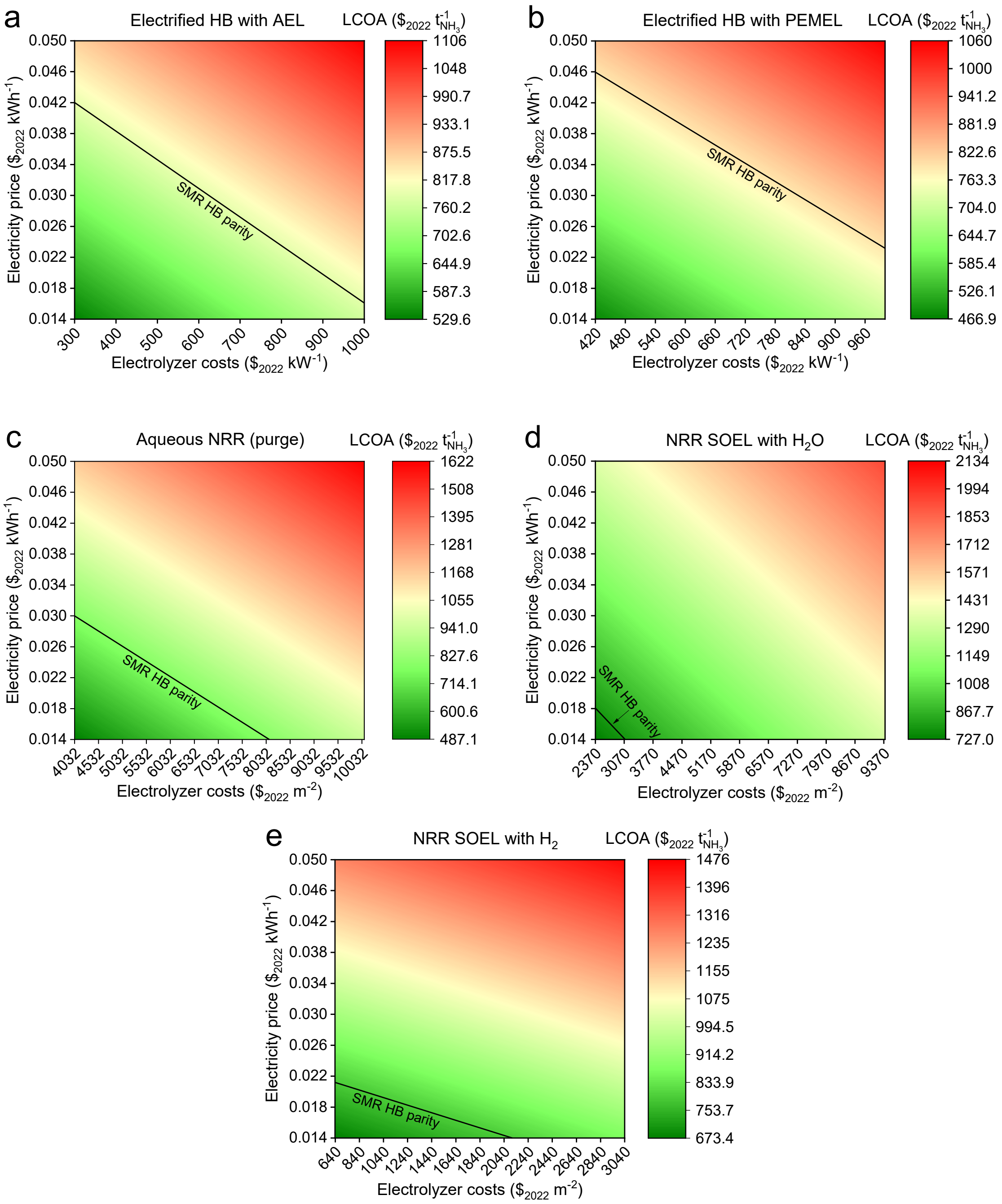

Figure S14. LCOA as a function of the electricity price and the electrolyzer costs for (a) electrified Haber-Bosch with AEL, (b) electrified Haber-Bosch with PEMEL, (c) aqueous NRR at ambient conditions with purge scenario, (d) NRR SOEL with water oxidation, (e) NRR SOEL with hydrogen oxidation. Base case assumptions for O_2, H_2O and energy import prices in 2050 are used. NRR electrolyzers operate at the ARPA-e aspirational values (0.3 A cm^{-2} and FE = 90%).

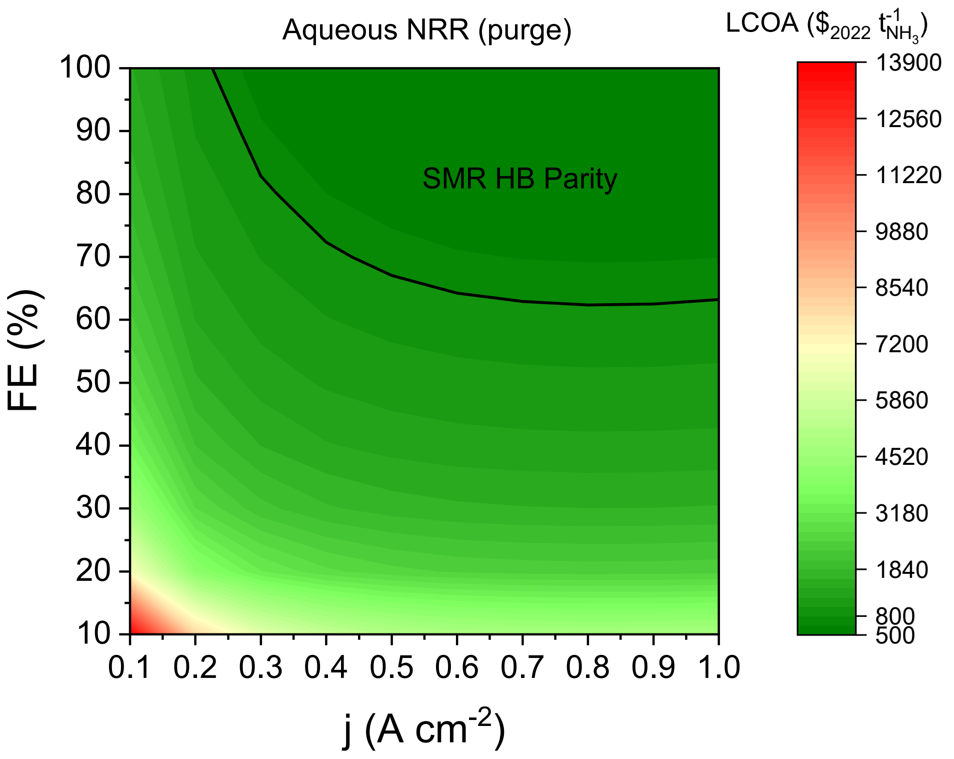

Figure S15. LCOA versus the FE and *j* for aqueous NRR at base case assumptions in 2050, using a more optimistic electricity price of \$0.02 per kWh.

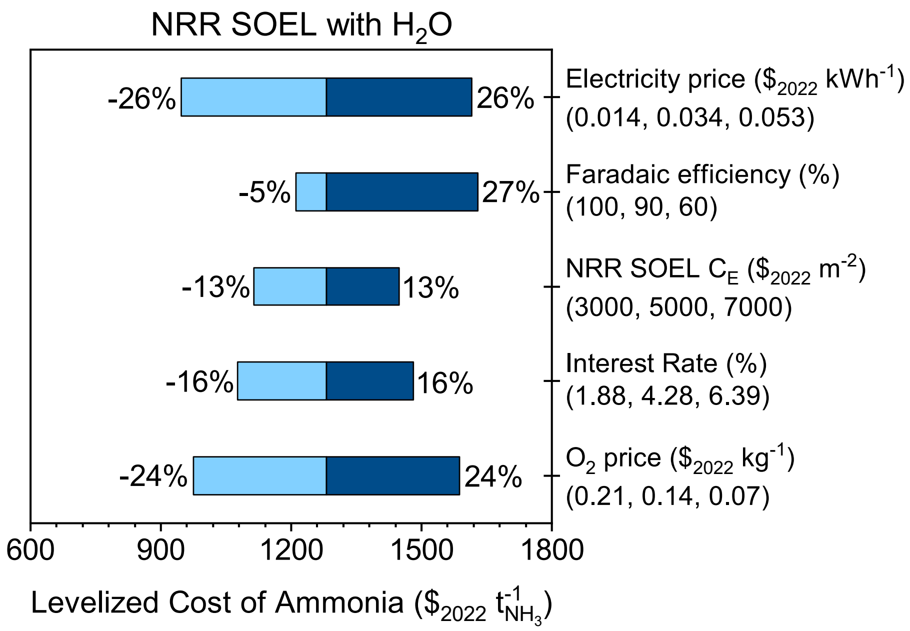

Figure S16. Sensitivity analysis of the NRR SOEL process with water oxidation at a constant *j* of 0.3 A cm^{-2}.

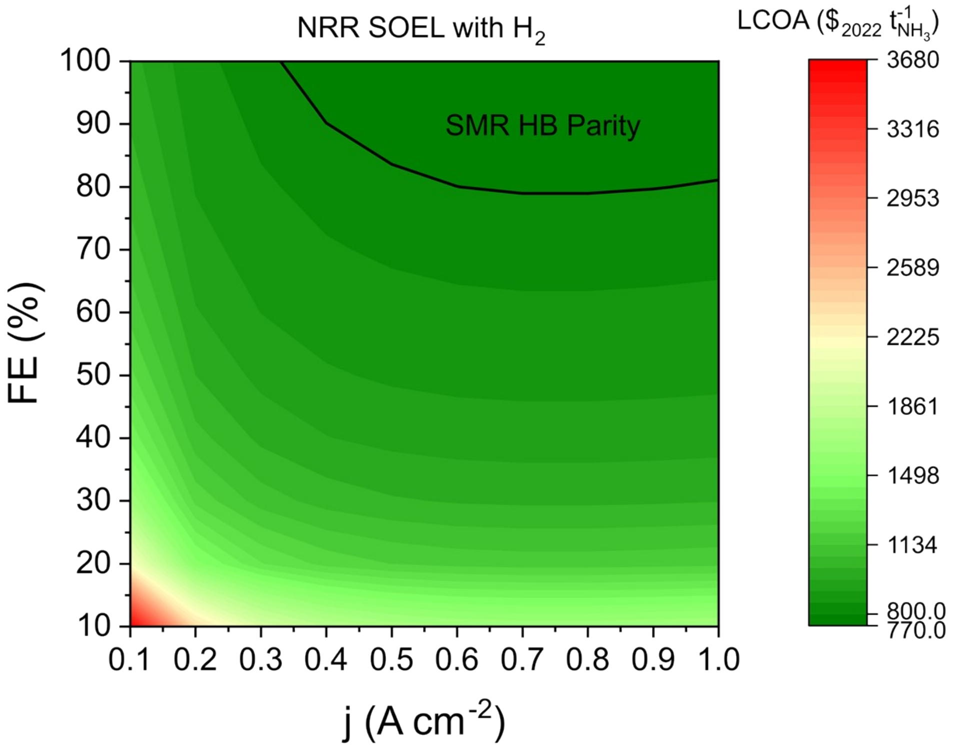

Figure S17. LCOA versus the FE and *j* for NRR SOEL with hydrogen oxidation at base case assumptions in 2050, using a more optimistic electricity price of $0.02 per kWh.

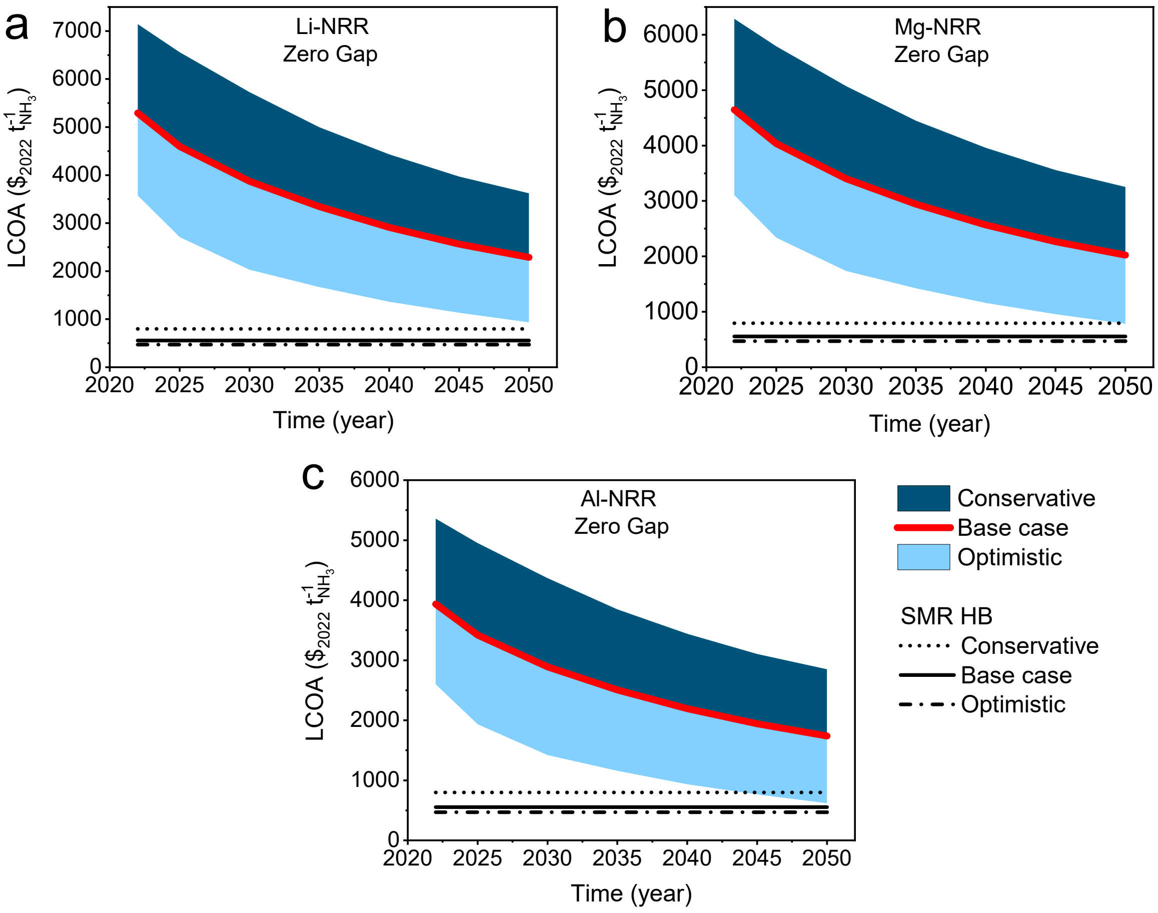

Figure S18. LCOA of mediated NRR processes using an "optimized" zero-gap membrane electrode assembly electrolyzer with (a) Li-NRR, (b) Mg-NRR and (c) Al-NRR.

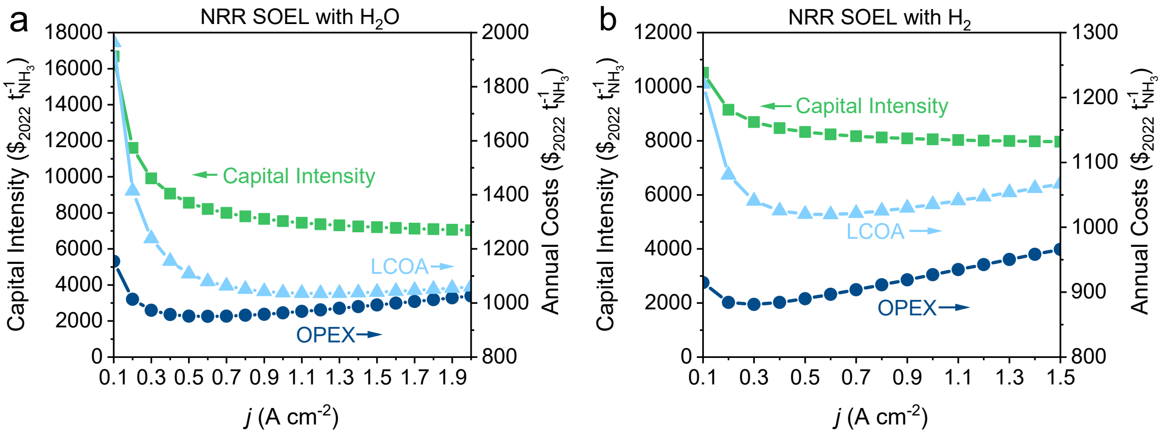

Figure S19. Current density as a function of the capital intensity, OPEX and LCOA for (a) NRR SOEL with water oxidation and (b) NRR SOEL with hydrogen oxidation at a FE of 90% using the base case assumption in 2050.

S3 Supplementary Tables

Table S15. An overview of the electrochemical model input parameters.

Quantity	Unit	Aqueous NRR (GDE)	NRR SOEL with OER	NRR SOEL with HOR	Li-NRR with HOR
E_{eq}^{0}	V	-1.17	-1.21	-0.14	-3.045
T	°C	25	550	550	25
P	atm	1	1	1	1
α_{cat}		0.5	0.5	0.5	0.4 [2]
α_{an}		0.5	0.5	0.5	0.5
n_{cat}	Electrons	6 (NRR)	6 (NRR)	6 (NRR)	1 (Li-plating)
n_{an}	Electrons	4 (OER)	4 (OER)	2 (HOR)	2 (HOR)
$j_{0,reduction}$	A cm^{-2}	10^{-22} [3] (NRR)	0.4 [4] (NRR)	0.4 [4] (NRR)	0.00001 [5] (Li)
$j_{0,oxidation}$	A cm^{-2}	10^{-12} [3] (OER)	0.13 [4] (OER)	0.53 [6] (HOR)	0.01 [7] (HOR)
R_{mem}	Ω cm	0.375 [8]	-	-	-
d_{mem}	mm	0.05 [8]	-	-	-
Electrolyte		1 M KOH	$Ce_{0.8}Sm_{0.2}O_2$	$Ce_{0.8}Sm_{0.2}O_2$	1 M LiFSI in THF
$\kappa_{electrolyte}$	S cm^{-1}	0.215 [9]	0.014 (650°C) [10]	0.014 (650°C) [10]	0.015 [11]
d_{gap}	mm	4	0.05 [10]	0.05 [10]	2

Table S16. Equipment cost data for conventional process units.[12-17]

**Equipment**	***a***	***b***	***S_{limits}***	***n***	***S_B***	***C_B* ($)**	***K_1***	***K_2***	***K_3***	**Equation**	**Ref**
Pump ($\$_{2010}$)	8000	240	0.2-126 L s^{-1}	0.9						S42	[12]
Compressor ($\$_{2010}$)	580000	20000	75-30000 kW	0.6						S42	[12]
Heat exchanger (U-tube and shell) ($\$_{2010}$)	28000	54	10-1000 m^2	1.2						S42	[12]
Vertical Pressure Vessels ($\$_{2010}$)	17400	79	120-250000 kg	0.85						S42	[12]
Vacuum pump (CEPCI 1000)			3-1670 L s^{-1}		170 L s^{-1}	22000				S43	[13]
Rotary compressor ($\$_{2001}$)			18-950 kW				5.0355	-1.8002	0.8253	S44	[14]
Heat exchanger (plate and frame) ($\$_{2010}$)	1600	210	1-500 m^2	0.95						S42	[12]
Storage tank (fixed roof) ($\$_{2001}$)			90-30000 m^3				4.8509	-0.3973	0.1445	S44	[14]
Make-up tank ($\$_{2010}$)	5800	1600	10-4000 m^3	0.7						S42	[12]
Cryogenic Distillation ($\$_{2010}$)				0.6	250 tN_2 d^{-1}	1823620				S43	[15]
ASU PSA ($\$_{2014}$)				1	89.98 tN_2 d^{-1}	565000				S43	[16]
N_2/H_2 PSA ($\$_{2011}$) (Total fixed capital costs)				0.6	66.72 $kmol_{H2}$ h^{-1}	2050585				S43	[17]

Table S17. Cost projections for electrolyzers, electricity, feedstock and commodity prices for conservative, base case and optimistic scenarios. The large collection of AEL and PEMEL cost estimates reported by Glenk et al.,[18] were used for our analysis. Their exponential fitting through the data points was extrapolated until 2050 and used as the base case scenario (for AEL and PEMEL). A collection of their highest and lowest reported estimates were used to inter- and extrapolate a trend until 2050, which was implemented as conservative and optimistic price assumptions. The base case capital costs for water SOEL were taken from Bohm et al., who used technology learning curve models to predict reductions in the manufacturing cost trend over time.[19] Optimistic and conservative price scenarios were extrapolated from survey data from Schmidt et al.[20] The equipment costs of the NRR electrolyzers were derived from water electrolyzer data and converted from $ per unit power to $ per unit area via their respective power densities (more details discussed in Section S4.9). The optimistic renewable electricity price is interpolated from utility solar PV price forcasts from ref [21,22]. The conservative scenario is adapted from Bogdanov et al. (North America).[23] The base case represents the average between the conservative and optimistic scenario. H_2 commodity pricing was extrapolated from ref [24]. The base case cost assumption for the O_2 price is the average Europe export tariff in 2021.[25] Optimistic cost price is the average O_2 price in Belgium in 2021.[25] Conservative O_2 price is assumed. Ultrapure H_2O price is 4-11 $ per m^3 based on Hausmann et al. by combining utility PV solar with reverse osmosis.[26] The natural gas price was derived by using statistical analysis from the Henry Hub historical data between 1997-2023. The first quartile (optimistic), median (base case) and third quartile (conservative) of the Henry Hub natural gas spot price historical data (1997-2023) were implemented for the scenarios.[27] The CO_2 tax is based on the IPCC 2022 mitigation report (chapter 11).[28] All data is inflation corrected to 2022.

Quantity	Scenario	2022	2025	2030	2035	2040	2045	2050	Unit
C_E	Conservative	1773	1639	1439	1263	1108	973	854	$ per kW
water AEL[a]	Base Case	1307	1194	1028	884	761	655	564	
	Optimistic	839	751	623	517	429	356	296	
C_E	Conservative	2689	2237	1646	1211	891	656	482	$ per kW
water PEMEL[a]	Base Case	1901	1641	1284	1005	787	615	482	
	Optimistic	1258	1120	922	760	626	515	425	
C_E	Conservative	5550	5166	4583	4067	3608	3201	2840	$ per kW
water SOEL[b]	Base Case	3095	2688	1830	1259	954	831	758	
	Optimistic	3081	2513	1789	1274	907	645	460	
C_E	Conservative	24985	21706	17273	13853	11196	9118	7482	$ per m^2
aqueous NRR[c]	Base Case	17963	15876	12946	10581	8667	7115	5854	
	Optimistic	11745	10474	8653	7149	5907	4880	4032	
C_E	Conservative	32859	30583	27135	24076	21362	18954	16817	$ per m^2
NRR SOEL with	Base Case	18326	15912	10832	7451	5649	4921	4486	
H_2O[d]	Optimistic	18241	14876	10591	7150	5368	3822	2721	
C_E	Conservative	8852	8239	7310	6486	5755	5106	4531	$ per m^2
NRR SOEL with	Base Case	4937	4287	2918	2007	1522	1326	1209	
H_2[e]	Optimistic	4914	4008	2853	1926	1446	1030	733	
C_E	Conservative	79588	69142	55023	44127	35663	29045	23832	$ per m^2
Li-NRR[f]	Base Case	57219	50572	41239	33704	27607	22664	18649	
	Optimistic	37413	33363	27564	22774	18816	15546	12845	
C_E	Conservative	33508	29111	23166	18579	150151	12229	10034	$ per m^2
Li-NRR[f]	Base Case	24091	21292	17363	14190	1623	9542	7852	
(MEA-type)	Optimistic	15752	14047	11605	9588	7922	6545	5408	
C_E	Conservative	27941	24273	19317	15491	12520	10197	8367	$ per m^2
Mg-NRR[f]	Base Case	20088	17754	14478	11832	9692	7957	6547	
(MEA-type)	Optimistic	13134	11713	9677	7994	6606	5458	4509	
C_E	Conservative	21784	19003	15123	12128	9802	7983	6550	$ per m^2
Al-NRR[f]	Base Case	15726	13899	11334	9263	7588	6229	5125	
(MEA-type)	Optimistic	10283	9170	7576	6259	5171	4273	3530	
Electricity price	Conservative	0.049	0.03	0.02	0.018	0.016	0.015	0.014	$ per kWh
	Base Case	0.056	0.046	0.041	0.038	0.036	0.0345	0.034	
	Optimistic	0.062	0.062	0.061	0.058	0.056	0.054	0.053	
H_2 price	Conservative	4.5	2.76	2.27	2.03	1.91	1.83	1.77	$ per kg
	Base Case	4	2.47	2.02	1.81	1.68	1.61	1.56	
	Optimistic	3.25	2.15	1.73	1.54	1.42	1.36	1.31	
O_2 price	Conservative	0.07	0.07	0.07	0.07	0.07	0.07	0.07	$ per kg
	Base Case	0.14	0.14	0.14	0.14	0.14	0.14	0.14	

	Optimistic	0.21	0.21	0.21	0.21	0.21	0.21	0.21	
Ultrapure H_2O	Conservative<br>Base Case<br>Optimistic	11<br>7.5<br>4	11<br>7.5<br>4	11<br>7.5<br>4	11<br>7.5<br>4	11<br>7.5<br>4	11<br>7.5<br>4	11<br>7.5<br>4	\$ per m^3
Natural gas	Conservative<br>Base Case<br>Optimistic	5.66<br>3.77<br>2.79	5.66<br>3.77<br>2.79	5.66<br>3.77<br>2.79	5.66<br>3.77<br>2.79	5.66<br>3.77<br>2.79	5.66<br>3.77<br>2.79	5.66<br>3.77<br>2.79	\$ per GJ
CO_2 Tax	Conservative<br>Base Case<br>Optimistic	175<br>58<br>23	175<br>58<br>23	175<br>58<br>23	175<br>58<br>23	175<br>58<br>23	175<br>58<br>23	175<br>58<br>23	\$ per tCO_2

[a] Extrapolated costs from Glenk et al.[18]
[b] Extrapolated costs from Schmidt et al and Bohm et al.[20,19]
[c] Average equipment cost between AEL and PEMEL. Converted to \$ per area with a power density of 11.2 kW per m^2 (see Table S24).
[d] \$ per kW price as a water SOEL. Converted to \$ per area with a power density of 5.92 kW per m^2 (see Table S24).
[e] \$ per kW price as a water SOEL. Converted to \$ per area with a power density of 1.59 kW per m^2 (see Table S24).
[f] \$ per kW price as aqueous NRR. Converted to \$ per area with a power density of 35.7 kW per m^2 (see Table S24).

Table S18. Lang factors adapted from "Smith – Chemical Process Design and Integration".[29]

Inside Battery Limit (ISBL)	f_M	1 - 3.4*
	f_P	1 - 1.9*
	f_T	1 - 2.1*
	f_{pip}	0.7
	f_{erec}	0.4
	$f_{I\&C}$	0.2
	f_{elec}	0.1

$$C_{ISBL} = C_E\left(f_m f_p f_T\left(1+f_{pip}\right)+f_{er}+f_{I\&C}+f_{elec}\right)\#(S1)$$

Outside Battery Limit (OSBL)	f_{util}	0.5
	$f_{off\text{-}sites}$	0.2
	f_{build}	0.2
	$f_{side\ prep}$	0.1

$$C_{OSBL} = C_E\left(f_{util}+f_{off-sites}+f_{build}+f_{side\ prep}\right)\#(S2)$$

Total fixed capital cost (TFC)	$f_{design\&eng}$	1
	f_{cont}	0.2

$$C_{TFC} = \frac{C_{ISBL}+C_{OSBL}+C_E f_{design\&eng}}{f_{cont}}\#(S3)$$

Total capital cost (TC)	$f_{work\ cap}$	0.2

$$C_{TC} = \frac{C_{TFC}}{f_{work\ cap}}\#(S4)$$

* Factors are process condition dependent.

Table S19. General assumptions for the OPEX.

Days of operation	333.3	days
Workers	22	
Annual work hours	1791	hours/year
Salary	71640	$/year
O&M	3 % of Total Capital [30]	$
SMR H-B Consumables	3 [30]	$/t NH_3
Interest rate	4.28	%

S4 Supplementary Methods

S4.1 NRR Electrolyzers

S4.1.1 Gibbs Free Energy and the Equilibrium Potential

The following half-reactions were considered for direct and indirect electrochemical NH_3 synthesis.

$$N_2 + 6\,H_2O + 6\,e^{-} \rightarrow 2\,NH_3 + 6\,OH^{-} \quad (NRR) \# (S5)$$

$$6\,OH^{-} \rightarrow \frac{3}{2}O_2 + 3\,H_2O + 6e^{-} \quad (OER) \# (S6)$$

$$N_2 + 3\,H_2O \rightarrow 2\,NH_3 + \frac{3}{2}O_2 \quad (Overall\ reaction) \# (S7)$$

$$N_2 + 6\,H^{+} + 6\,e^{-} \rightarrow 2\,NH_3 \quad (NRR) \quad \# (S8)$$

$$3\,H_2 \rightarrow 6\,H^{+} + 6e^{-} \quad (HOR) \# (S9)$$

$$N_2 + 3\,H_2 \rightarrow 2\,NH_3 \quad (Overall\ reaction) \# (S10)$$

The standard Gibbs free energy is the thermodynamic minimum of the reaction (S7 and S10) and can be calculated by Equation S11 using tabulated standard enthalpies and entropies of formation (NIST database) and stoichiometric coefficients of each reactant and product. Equation S12 shows a calculation example for aqueous NRR.

$$\Delta G^0 = \Delta H^0 - T\Delta S^0 \# (S11)$$

$$\begin{aligned} \Delta G^0 &= \left(\left[v_{O_2}\Delta H_f^{O_2} + v_{NH_3}\Delta H_f^{NH_3}\right] - \left[v_{N_2}\Delta H_f^{N_2} + v_{H_2O}\Delta H_f^{H_2O}\right]\right) \\ &\quad - T\cdot\left(\left[v_{O_2}\Delta S_f^{O_2} + v_{NH_3}\Delta S_f^{NH_3}\right] - \left[v_{N_2}\Delta S_f^{N_2} + v_{H_2O}\Delta S_f^{H_2O}\right]\right) \\ &= ([1.5\cdot 0 + 2\cdot -45.9] - [1\cdot 0 + 3\cdot -285.83]) \\ &\quad - 98\cdot([1.5\cdot 205.15 + 2\cdot 192.77] - [1\cdot 191.61 + 3\cdot 69.95])\cdot 10^{-3} \\ &= (-91.8 + 857.49) - 298\cdot(693.265 - 401.46)\cdot 10^{-3} = 765.69 - 86.95789 \\ &= 678.73211\,kJ = 339.366\,\frac{kJ}{mol\,NH_3} = 19.928\,\frac{GJ}{tNH_3} \# (S12) \\ &\# \end{aligned}$$

The standard Gibbs free energy is related to the standard equilibrium potential via the Faraday constant (96485 C/mol). Three electrons are required to produce one mol of NH_3, thus for aqueous NRR:

$$E_{eq}^0 = -\frac{\Delta G^0}{nF} = -\frac{339366}{3\cdot 96485.33} = -1.172\,V \# (S13)$$

The equilibrium potential is calculated via the Nernst law with N_2, O_2 and NH_3 partial pressures of 1, 1 and 0.1 atm, respectively:

$$E_{eq} = E_{eq}^0 - \frac{RT}{nF}\ln\left(\frac{p_{NH_3}^2}{p_{N_2}p_{O_2}^{1.5}}\right) \# (S14)$$

S4.1.2 Activation Overpotentials and Ohmic Losses

An additional overpotential is required to overcome the activation barrier of an electrochemical reaction. It is estimated that the minimum overpotential for NRR must be at least 0.4 V.[31,32] The activation overpotential increases with the current density and can be estimated by approximations of the Buttler-Volmer equation. If the exchange current density (j_0) is relatively small with respect to j ($j/j_0 > 4$) the Tafel equation (Equation S15 and S16) can be considered. In case j_0 is large ($j/j_0 < 1$), which is often the case for high temperature electrolyzers, the hyperbolic sine approximation (Equation S17 and S18) is more appropriate.[33]

$$\eta_{cat} = -\frac{RT}{n_{cat}F\alpha}\ln\frac{j}{j_{0,c}}\#(S15)$$

$$\eta_{an} = \frac{RT}{n_{an}F\alpha}\ln\frac{j}{j_{0,a}}\#(S16)$$

$$\eta_{cat} = -\frac{RT}{n_{cat}F\alpha}\sinh^{-1}\left(\frac{j}{j_0}\right)\#(S17)$$

$$\eta_{an} = \frac{RT}{n_{an}F\alpha}\sinh^{-1}\left(\frac{j}{j_0}\right)\#(S18)$$

The ohmic losses are associated with the transport of ions in the membrane (Equation S19) and the electrolyte (Equation S20). The membrane transport losses are usually small because they are < 1 mm. On the contrary, the gap between the working and the counter electrode (d_{gap}) in liquid electrolyzers can be up to a few mm thick. This means that electrolytes with a poor conductivity will have a large influence on the overall cell voltage. The concentration overpotential due to mass transport limitations is not considered in this work.

$$\eta_{mem} = j \cdot d_{mem} \cdot R_{mem}\#(S19)$$

$$\eta_{\Omega} = \frac{j \cdot d_{gap}}{\kappa}\#(S20)$$

S4.2 H_2 Electrolyzers

The energy consumption of the AEL and PEMEL for electrified Haber-Bosch and the indirect NRR processes are based on commercially available models from Nel (A485) and Siemens (Silyzer 300), respectively.[34] All relevant details are listed in Table S20.

Table S20. H_2 electrolyzer operating conditions based on commercially available electrolyzers. Data acquired from ref [34-36].

Quantity	Unit	AEL (Nel A485)	PEMEL (Siemens Silyzer 300)
E_{system}	$kWh\ Nm_{H_2}^{-3}$	4	4.59
EE_{LHV}[a]	%	75	65
T	C	80	80
P	Bar	1.013	35
H_2O Consumption	$L_{H_2O}\ kg_{H_2}^{-1}$	10	10

[a] based on the LHV of H_2 (3 kWh Nm^{-3})

S4.3 Air Separation Unit

Pressure swing adsorption (PSA) for air separation is economically attractive at N_2 capacities < 500 t per day.[37] The energy consumption of a PSA ASU depends on the purity of the N_2 product stream, which can very between 1.12 – 1.584 GJ per tN_2 with corresponding purities between 98 – 99.9 vol% N_2.[38] Vast quantities of O_2 can effect the current efficiency, therefore it is desired to have the highest possible N_2 feed purity. Hence, we assume a PSA energy consumption of 1.584 kJ per tN_2. The energy consumption of the cryogenic distillation unit can vary between 0.44 – 1.33 GJ per tN_2, which depends mainly on the N_2 capacity and final gas pressure.[38] The N_2 demand for our process is relatively small compared to a industrial scale Haber-Bosch plant, therefore we assume a single cryogenic column, which is less capital intensive, but consumes more energy (1.33 GJ per tN_2).

S4.4 Heat Exchangers, Compressors and Pumps

The necessary heating or cooling duties of all exchangers are calculated in Aspen Plus, which uses the first law of thermodynamics. The actual energy input in the form of work depends on the exchange medium, wherein steam (from an electric boiler) was used for hot utilities, cooling water (CW) for cold utilities up to 35 °C, and various refrigerants for cold utilities < 35 °C. The energy input of the electric steam boiler can simply be calculated with Equation S21 implementing a boiler efficiency of 0.95.[39,40]

$$W_{boiler} = \frac{Q_{demand}}{\eta_{boiler}} \#(S21)$$

The amount of required cooling water for the intercoolers is calculated by:

$$Q_{intercooler} = \dot{m}_{CW} \cdot C_{P,H_2O} \cdot \Delta T \#(S22)$$

Where Q is obtained from Aspen and a ΔT of 10 °C is assumed. Additional work input is required for the cooling water pumps, which can be calculated by the following heuristic:[41]

$$W_{CW} = \frac{0.0972 \cdot \dot{m}_{CW} \cdot \Delta P}{\eta_{pump}} \#(S23)$$

With $\dot{m}_{CW}$, the cooling water mass flow obtained from Q_{cool}, ΔP is the pressure drop in the tubing (assumed to be 2 bar) and η_{pump} is 85% for a reciprocating pump.

The cold utilities < 35 °C are based on a Carnot refrigeration cycle, in which the duty of the compressor can be calculated by the coefficient of performance (COP):

$$COP = \frac{Q}{W_{comp}} = \frac{0.6T_{evap}}{(T_{cond} - T_{evap})} \#(S24)$$

Where T_{evap} is the evaporation temperature of the selected refrigerant and T_{cond} is the temperature of the condenser. We assume a minimum temperature difference of 10 °C between the hot and cold stream (for all heat exchangers). Therefore, T_{cond} is atleast -10 °C lower than the temperature of the hot stream.

The area of all exchangers were obtained from Aspen Plus, which calculates the overall heat transfer coefficient and the logarithmic mean temperature difference between the hot and cold streams (Equation S25). A U-tube and shell type is considered in case A > 10 m^2. A plate and frame model is selected for smaller heat exchangers. The equipment cost of pumps and refrigeration compressors are also included in the total capital costs.

$$Q = U \cdot A \cdot \Delta T_{LM} \#(S25)$$

All compressors are simulated in Aspen Plus as polytropic using the ASME calculation method. The pressure ratio is 2.5. The polytropic and mechanical efficiencies are 0.75 and 0.95, respectively. The pressure ratio determines the required compressor stages to reach the desired final pressure. As an example, O_2 needs to be pressurized up to 163 bar to reach market requirements. Therefore, a six multistage compressor including intercoolers is implemented (see Figure S20). We assumed that the 1st intercooler has no ΔP, the 2nd intercooler a ΔP = 0.5 psi, and the 3rd intercooler a ΔP of 1 psi or 1 bar in case the pressure > 15 bar. The total energy input of a multistage compressor can be expressed as:

$$W_{comp} = \sum_{i=1}^{No.\ stages} (W_{stage,i} + W_{CW,i}) \#(S26)$$

The total equipment cost of the compressor unit includes the individual compressor stages, U-tube and shell intercoolers and CW pumps.

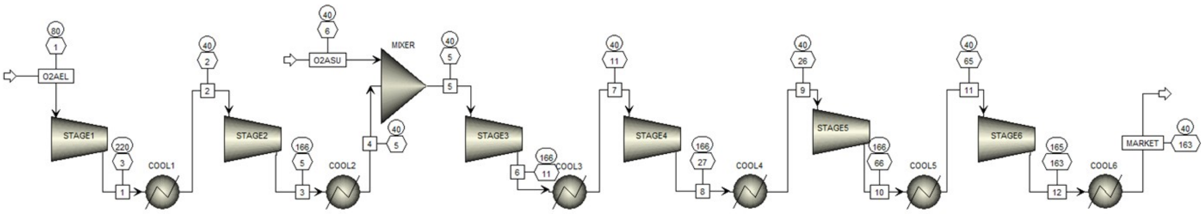

Figure S20. Aspen simulation of a multistage O_2 compressor with intercooling.

S4.5 Distillation

Distillation columns were designed in Aspen Plus using the RADFRAC model. The column was optimized at an NH_3 distillate purity of 99.5% and NH_3 recovery of 99.9%. Figure S7 shows that the NH_3 feed composition has a significant influence on the reboiler duty. We assume a NH_3 feed composition of 10 mol% to minimize the reboiler duty. In general, a minimum amount of stages is required to ensure the desired distillate purity. Beyond this minimum, the number of stages is a trade-off between the equipment and operational costs, since more stages reduce the energy input of the condenser and reboiler. Herein, we focused particularly on minimizing the energy input. The column design specs are illustrated in Figure S21.

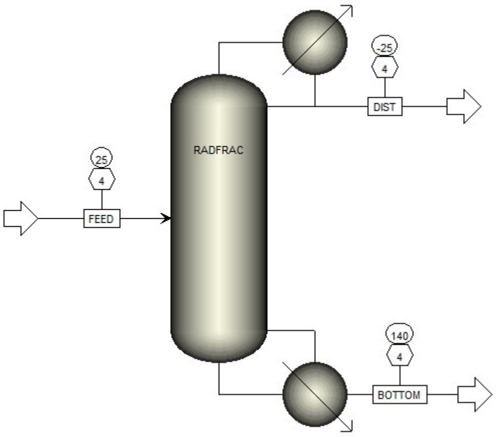

**Quantity**	**Unit**	**Aqueous NRR (ambient)**	**Li-NRR**
$N_{theoretical}$	#	17	14
Feed stage	#	8	9
Top stage T	C	-24.8	-33.6
Bottom stage T	C	140.16	49.9
Condenser Duty	kW	-806.8	-2963.8
Reboiler Duty	kW	6967	4116.4
Reflux Ratio		0.826	1.02
Boilup Ratio		0.31	0.24
N_{actual}	#	20	16
L_C	m	10	8
D_C	m	1.322	3.427

Figure S21. Example of the RADFRAC model in Aspen Plus (left) and its design specs (right). ELECNRTL was used as the property package.

The column sizing was based on standard methods available in chemical engineering textbooks, such as "Towler and Sinnot – Chemical Engineering Design".[12] The actual number of stages to estimate the column length is calculated via the plate efficiency:

$$N_{actual} = \frac{N_{theoretical}}{\eta_{plate}} \#(S27)$$

$$\eta_{plate} = \frac{log\left(1 + \eta_{mw}\left(\frac{mV}{L} - 1\right)\right)}{\log\left(\frac{mV}{L}\right)} \#(S28)$$

$$L_C = \frac{N_{actual}}{l_{plate}} \#(S29)$$

With a murphy plate efficiency (η_{mw}) of 0.9 for an ammonia-water mixture,[12] m the slope of the equilibrium line, l_{plate} the plate spacing of 0.5 m, V and L the molar vapor and liquid flow rate, respectively. The diameter of the column is obtained with the vapor flow rate and the maximum allowable superficial velocity (u_v) using the Souders-Brown equation:

$$D_c = \sqrt{\frac{4V_w}{\pi\rho_v u_v}} = 3.43\ m \#(S30)$$

$$u_v = \left(-0.171 l_{plate}^2 + 0.27 l_{plate} - 0.047\right)\left[\frac{\rho_L - \rho_v}{\rho_v}\right]^{0.5} \#(S31)$$

Where V_w is the mass vapor flow rate, ρ_v the distillate density and ρ_L the bottom liquid density. The wall thickness is related to the maximum allowable stress (σ_{max}), D_c, and the pressure:

$$\sigma_{max} = \frac{PD_c + 1.2t_{wall}P}{2t_{wall}} \#(S32)$$

The design pressure is assumed to be 10% above the working pressure. Values for σ_{max} are tabulated for different steels and temperatures, which can be used to extract t_{wall}. In our case, the column is made from stainless steel grade 304. The head and closure of the column are assumed to have a hemicircular shape and require 60% of the column wall thickness. The sum of the column, condenser and reboiler represent the total equipment cost of the distillation unit.

S4.6 Adsorption

The adsorption column was designed and optimized in Aspen Adsorption. The adsorption cycle consists of adsorption, column regeneration by heating under vacuum and cooling. Zeolite 13x is selected as adsorbent material. The gas adsorption equilibrium isotherms were modelled with the Langmuir approach:

$$q = \frac{q_s \cdot b \cdot p}{1 + b \cdot p} \#(S33)$$

Where, q is the adsorbed gas concentration, q_s is the saturation sorbate concentration, b is the adsorption equilibrium constant. The kinetics of the adsorption and desorption process is also influenced by temperature. Therefore, the adsorption equilibrium constant for NH_3 is expanded in the form of the van 't Hoff equation;[42]

$$b_{NH_3} = b_{0,NH_3} \cdot e^{\left(-\frac{\Delta H_{ads,NH_3}}{RT}\right)} \#(S34)$$

Table S21. Adsorption equilibrium isotherm data acquired from Helminen et al. and Park et al.[42,43]

Quantity		Unit
q_s	7.51 [42] (NH_3)<br>3.16 [43] (N_2)<br>4.15 [43] (H_2)	mmol g^{-1}
$b_{0,NH3}$	0.735 [42] (NH_3)	kPa^{-1}
b	$1.2 \cdot 10^{-3}$ [43] (N_2)<br>$5.34 \cdot 10^{-5}$ [43] (H_2)	kPa^{-1}
ΔH_{ads}	63.3 [42] (NH_3)	kJ mol^{-1}

With ΔH_{ads} representing the heat of adsorption that is specific for the adsorbent material. T_{ads} is set to 313 K in order to minimize the cooling cost from the SOEL product stream and regeneration step. The adsorption capacity is generally higher at room temperature. The adsorbed gas concentration N_2 and H_2 are to some extent inert to the zeolite, hence Equation S34 is not included in the Langmuir model for N_2 and H_2. The adsorption time, t_{ads}, was set to 600 s, which is slightly before NH_3 breakthrough occurs as shown in Figure S22. The fixed bed mass transfer coefficients for the gases (Equation S35) can be derived from the Colburn-Chilton correlations for the diffusion mass transport (Equation S36 and S37), where v_s is the superficial velocity.[44]

$$k = 1.17 \cdot v_s \cdot Re^{-0.415} Sc^{-0.667} \#(S35)$$

$$j_D = \frac{k}{v_s} \cdot Sc^{0.667} \#(S36)$$

$$j_D = 1.17 Re^{-0.415} \quad 10 < Re < 2500 \#(S37)$$

Aspen Adsorption uses time-dependent partial differential equations (PDEs) to solve the mass, momentum and energy balances during the dynamic simulation. The first-order upwind difference scheme (UDS1) with 40 nodes in 1D was used to discretize the PDEs. The material balance equations govern the adsorption kinetics and the mass transfer coefficients which are solved via a linear lumped resistance model. For the momentum balance, the pressure drop is calculated via the Ergun equation.[45] It is assumed that the column is isothermal. By using the model input parameters of Table S21 and S22, the optimal length (3 m) and diameter (2 m) of the column were obtained from Aspen Adsorption.

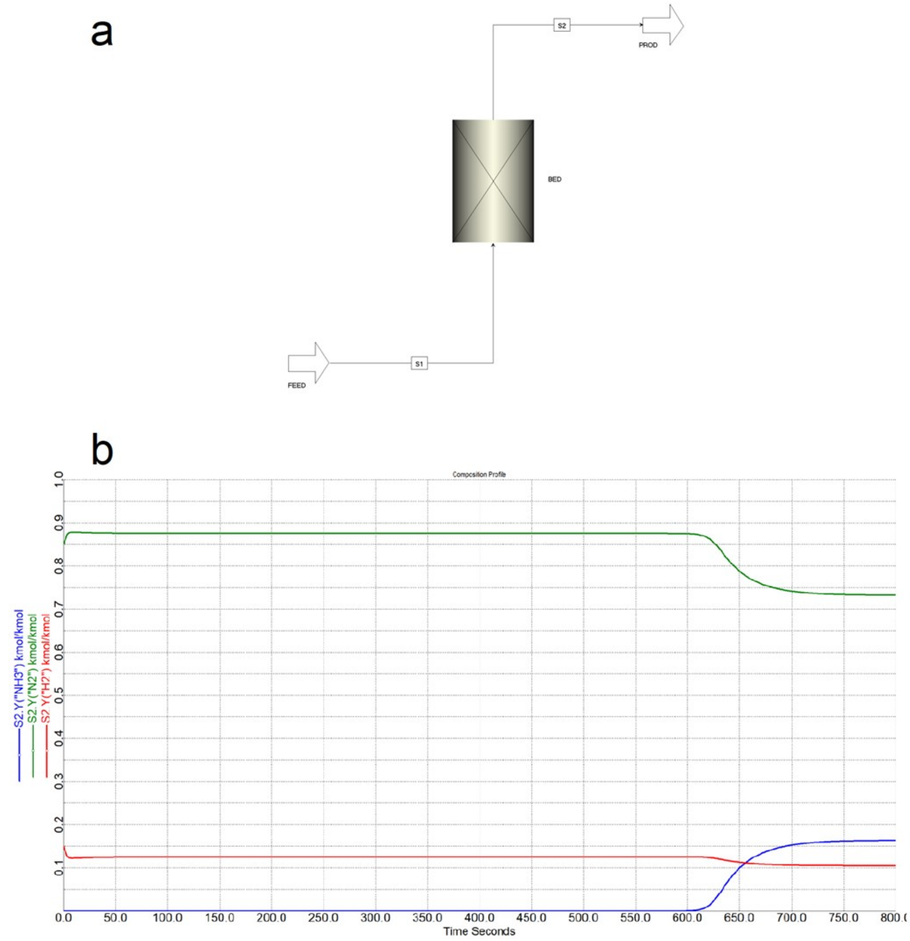

Figure S22. (a) Example of the model in the Aspen Adsorption simulation environment. (b) Obtained NH_3 breakthrough curve.

Table S22. A summary of the Aspen Adsorption model parameters.

Quantity		Unit
D_{bed}	2	m
h_{bed}	3	m
$d_{zeolite13x}$	2 [42]	mm
$\rho_{zeolite13x}$	647 [42]	kg m^{-3}
$C_{p,zeolite13x}$	943 [46]	J kg^{-1} K^{-1}
SF	1	
ε_i	0.35	
ε_p	0.6	
T_{ads}	313.15	K
v_s	0.42 [45]	m s^{-1}
p_i	56.98 (NH_3)<br>256.40 (H_2)<br>36.63 (N_2)	kPa
μ	$9.815 \cdot 10^{-6}$ (NH_3)<br>$1.746 \cdot 10^{-5}$ (N_2)<br>$8.743 \cdot 10^{-6}$ (H_2)	cP
ρ_{gas}	0.398 (NH_3)<br>2.948 (N_2)<br>0.030 (H_2)	kg m^{-3}
D_{gas}	$2.28 \cdot 10^{-5}$ [47] (NH_3)<br>$2.19 \cdot 10^{-5}$ [48] (N_2)<br>$8.5 \cdot 10^{-5}$ [49] (H_2)	m^2 s^{-1}
K	0.031 (NH_3)<br>0.041 (N_2)<br>0.044 (H_2)	s^{-1}

Although not modelled, it is assumed that the fixed bed is heated internally by steam during the regeneration step. Since the volume is relatively large, we assume that t_{reg} is 1500 s. The heat input can be estimated by rewriting the heat balance over the column during the regeneration step:[50]

$$Q_{reg} = \frac{m_{zeolite}\left(C_{p,zeolite} \cdot \left(T_{reg} - T_{ads}\right) + \Delta H_{ads} \cdot \left(q_{ads} - q_{reg}\right)\right)}{t_{reg}} \quad \#(S38)$$

Where, q_{ads}-q_{reg} is the work capacity of the fixed bed and T_{reg} = 473.15 K. It is not possible to desorb all NH_3, therefore a 90% recovery is assumed. The heat is supplied by steam from an electric boiler with an efficiency of 95%.[39] The vacuum pump power is calculated via the following heuristic:[51]

$$W_{vacuum} = 21.4 \cdot (SF)^{0.924} \quad \#(S39)$$

$$SF = \frac{\frac{1}{2}\dot{m}_{NH_3} \cdot \sqrt{273.15 + T_{reg}} \cdot 28.96}{293.15 \cdot Mw_{NH_3} \cdot P_{vacuum}} \#0.02 < SF < 16 \qquad \#(S40)$$

Where SF is the size factor and $\dot{m}_{NH3}$ is the NH_3 mass flow leaving the column. To calculate the cooling duty in order to reach T_{ads}, the heat of adsorption can be excluded from the heat balance:

$$Q_{cool} = \frac{m_{zeolite} \cdot C_{p,zeolite} \cdot \left(T_{reg} - T_{ads}\right)}{t_{cool}} \quad \#(S41)$$

With t_{cool} = 1100 s (assumed). Nonetheless, the usage of cooling water will only include the work of the cooling pump as explained earlier in section S4.4. Hence, the total energy input for the adsorption column is:

$$W_{ads} = W_{compression} + \frac{Q_{reg}}{\eta_{steam}} + W_{vacuum} + W_{CW} \#(S42)$$

By combing the adsorption, regeneration and cooling time in an adsorption schedule (Figure S23), six adsorption columns are required to enable continuous operation. Thus, the equipment cost of the adsorption unit consist of 6 columns, 1 compressor, 1 vacuum pump and a CW pump.

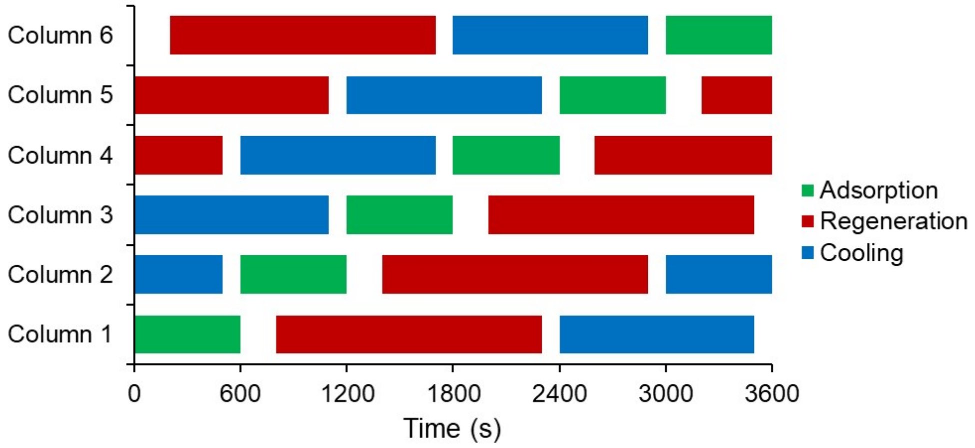

Figure S23. Adsorption, regeneration and cooling schedule of six adsorption columns.

S4.7 Storage Tanks

NH_3 is stored at -33 °C and 1 atm in a refrigerated double walled storage tank. The capacity of the storage tank is designed to accommodate 30 days of continuous production plus 10% freeboard.[15] The internal tank has a total volume of 4400 m^3 when taking a liquid NH_3 density of 682 kg per m^3. The internal tank is sized by D_{int}/H_{int} = 0.75 as a heuristic. The diameter of the external tank is 2 m wider than $D_{internal}$, while keeping the height constant. An additional refrigeration cycle is designed to reduce NH_3 boil-off losses, which are assumed to be 0.04% of the production capacity.[15]

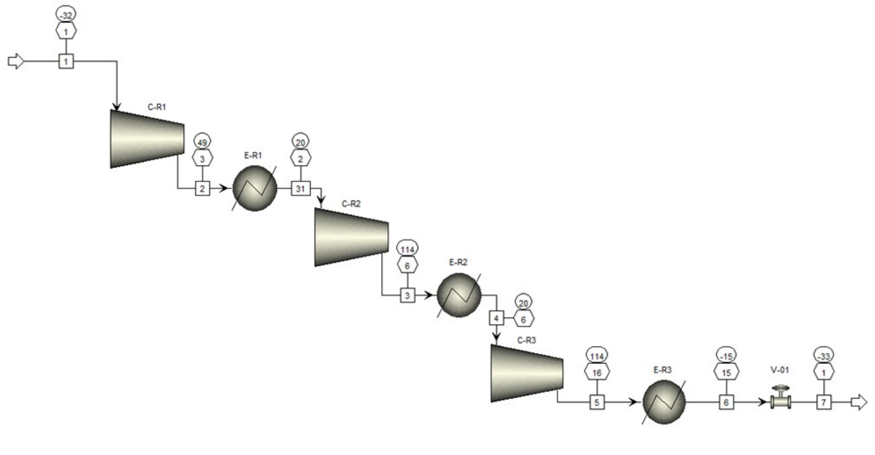

Figure S24. Refrigeration loop to recycle the NH_3 boil-off gasses.

S4.8 Haber-Bosch Synthesis Loop

For the electrified Haber-Bosch process with AEL, the feed gas compressor is simulated as a 6th stage compressor with CW intercoolers. The recycle stream enters the multistage compressor in the 6th stage (see Figure S1), but is simulated separately in Aspen (Figure S25).

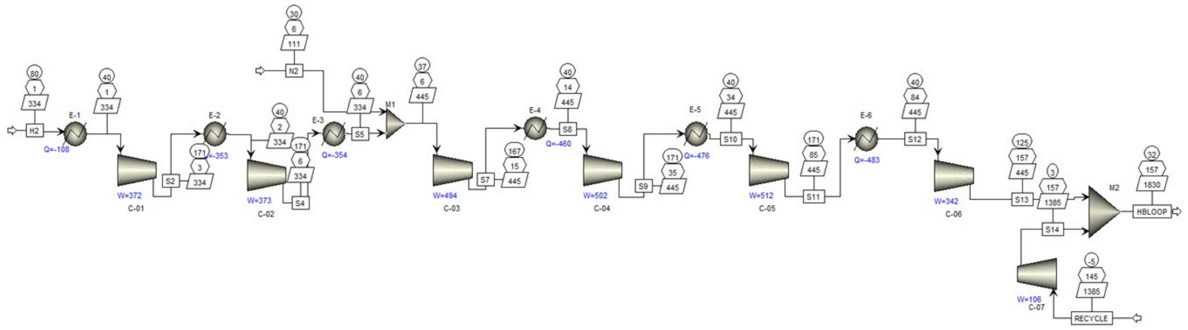

Figure S25. Aspen Plus simulation of the feed gas compressor in the electrified Haber-Bosch process.

Figure S26 shows the synthesis loop with heat integration, the reactor, condensation and a flash drum. The heat exchangers, including the coolers have a pressure drop of 1 bar. The Haber-Bosch reactor is modelled as a stoichiometric reactor (RStoic) at 400 °C and 155 bar with reaction S43.

$$N_2 + 3\ H_2 \rightarrow 2\ NH_3 \#(S43)$$

The Haber-Bosch reaction is exothermic, thus for convenience, the heat of reaction (53.8 kJ per mol) was added separately to the product stream. The product stream was used in the heat exchanger network for heat transfer to the reactant stream. Although not implemented here, it is possible to produce additional low pressure (1.57 GJ per tNH_3) or medium pressure steam (0.87 GJ per tNH_3) from the remaining heat for energy export.

The reactor is sized based on the catalyst bed. The total amount of required catalyst is calculated via Temkin-Pyzhev kinetics given by Equations S44-S46.[15] The input parameters are summarized in Table S23.

$$r_{NH_3} = 2 \cdot Mw_{NH_3} \frac{f}{\rho_{cat}} \left(k_1 \cdot \frac{p_{N_2} \cdot p_{H_2}^{1.5}}{p_{NH_3}} - k_2 \frac{p_{NH_3}}{p_{H_2}^{1.5}} \right) \left[\frac{kg\ NH_3}{kg\ cat\ hr} \right] \#(S44)$$

$$k_1 = 1.79 \cdot 10^4 \cdot e^{-\frac{20800}{RT}} \#(S45)$$

$$k_2 = 2.57 \cdot 10^{16} \cdot e^{-\frac{47400}{RT}} \#(S46)$$

Table S23. Haber-Bosch reaction kinetic data.

**Quantity**	**Unit**	
p_{N_2}	bar	37.589
p_{H_2}	bar	112.767
p_{NH_3}	bar	4.64
f (activity factor)		2
R	cal mol^{-1} K^{-1}	1.9872
ρ_{cat}	kg m^{-3}	2650

The dimensions of the catalyst bed can be scaled according to a reference reactor as is further discussed in Morgan et al. (page 142). Using this method, the height and diameter of the bed is 6.98 m and 0.58 m, respectively. Catalyst costs are categorized as consumables, which are listed as operational costs (see Table S19).

The flash drum has a 5 min half-full hold-up time,[52] thus with a liquid NH_3 stream of 123 L per min, the necessary volume is 1.23 m^3. As a heuristic, we assume an optimal L/D of 3, which can range between 2.5-5 m.[52] Thus, D and L are 0.8 m and 2.4 m, respectively. The wall thickness of both the reactor and the flash drum is calculated with a similar approach as the distillation column for the equipment cost.

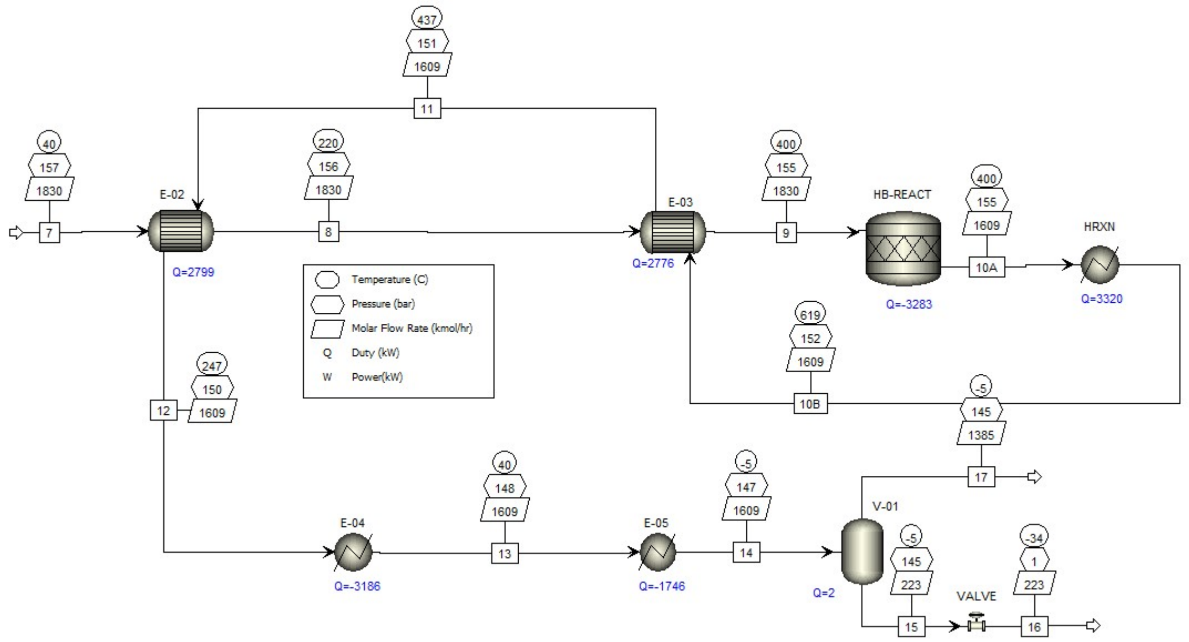

Figure S26. Aspen Plus simulation of the Haber-Bosch synthesis loop. RKS-BM was used as the property package.

S4.9 Techno-economic Assumptions

The capital costs of the NRR electrolyzers were derived from cost projections of commercial H_2O electrolyzers. Since these are given in $ per unit power ($ per kW), we used this metric as a base price. For the aqueous NRR and the Li-NRR electrolyzer, we assumed that their respective price is somewhere between the AEL and PEMEL, thus $C_{E,NRR} = (C_{E,AEL} + C_{E,PEMEL})/2$. The costs (in $ per kW) of the NRR SOEL are assumed to be the same as a water SOEL. The power density (kW per m^2) is used to convert $ per kW to $ per m^2. The latter is more useful if the *j* is used for the sensitivity analysis. Figure S27 shows that the $ per kW metric is insensitive to changes in the *j* because it is related to the electrolyzer power, which increases linearly with respect to the *j*, while the required electrode area decays exponentially with the *j*. For water electrolyzers, this issue is less relevant because the cost metrics ($ per kW) are already based on their performance criteria (0.4 A cm^{-2} for AEL and 2 A cm^{-2} for PEMEL).[20,34] Estimating the power density is somewhat arbitrary because its value depends strongly on the selected *j* and E_{cell}. To be consistent, a *j* of 0.4 A cm^{-2} (based on commercial AEL) was selected for all electrolyzers. The corresponding E_{cell} for AEL and PEMEL were taken from Buttler and

Spliethoff.[34] The E_{cell} (at 0.4 A cm^{-2}) of the NRR electrolyzers were calculated with our electrochemical model.

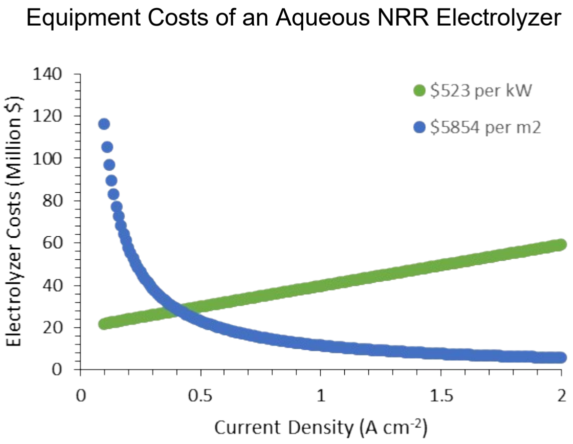

Figure S27. Comparison between two different methods for calculating the electrolyzer equipment cost. The Aqueous NRR electrolyzer is used as an example with cost data from Table S24.

The capital cost estimates of the electrolyzers for 2025 and 2050 are listed in Table S24. The capital costs of the AEL in 2025 ($8119 per m^2) are similar to earlier reported estimates ($5250 and $7800 per m^2).[20,53,54] It is evident that PEMEL ($10502 per m^2) is currently (2025) more expensive than AEL due to the requirement of expensive metals, such as Pt and IrO_x. Even higher estimates of ~$30000 per m^2 for PEMEL were reported elsewhere.[55] The stack of the AEL and PEMEL are usually around 40-50% of the total costs. The other 50-60% are system related equipment (balance of plant), such as rectifiers, heat exchangers, compressors, gas purifiers and storage facilities.[56] This means that the balance of plant (BoP) is different for each electrolyzer system.

The aqueous NRR electrolyzer is roughly 1.5 times more expensive ($15876 per m^2) than the PEMEL, which is justifiable because of the increased complexity of a GDE-type system. H_2O SOEL capital costs in the literature vary between $5600-16000 per m^2 with a more optimistic estimate from Schmidt et al. ($5600 per m^2),[20] and more conservative from Ramdin et al. (~$16000 per m^2).[54] In our case, the capital costs of the NRR SOEL with water ($15912 per m^2) is more comparable with the conservative estimate, while the NRR SOEL with H_2 ($4287 per m^2) is more similar to the estimate from Schmidt et al. NRR SOEL with H_2 oxidation is generally more stable and has a lower voltage drop across the ceramic material. This could mean that less reinforcement material is required with respect to NRR SOEL with H_2O oxidation, leading to a relatively lower stack cost. Additionally, the NRR SOEL with H_2 consumes less power, which can indicate that smaller and cheaper rectifiers are necessary.

The capital costs of the Li-NRR electrolyzer (~$50000 per m^2) is somewhat comparable to the chlor-alkali process (~$30000 per m^2),[53] which is known to be capital intensive. Our cost of merit is reasonable considering the complexity of the Li-NRR system (GDE-based, organic electrolyte, moisture free operation, etc) and the fact that the power density is higher than the chlor-alkali electrolyzer (35.7 vs. 15 kW per m^2).

At last, it is expected that future electrolyzers will become significantly cheaper due to constant investment in research & development and scale-up of the manufacturing capacity.[20] This is reflected in our capital cost estimates for 2050, which allowed us to investigate the relationship between electrolyzer costs and the LCOA, but also to estimate the necessary cost reductions to achieve SMR Haber-Bosch parity.

Table S24. Electrolyzer equipment cost estimation.

Quantity	Unit	AEL	PEMEL	Aqueous NRR	NRR SOEL with H_2O	NRR SOEL with H_2	Li-NRR
j	A cm^{-2}	0.4	0.4	0.4	0.4	0.4	0.4
E_{cell} [a]	V	1.7 [34]	1.6 [34]	2.8	1.5	0.4	8.9
$P_{density}$	kW m^{-2}	6.8	6.4	11.2	5.92	1.59	35.7
C_E (2025)	$\$_{2022}$ kW^{-1}	1194	1641	1418	2688	2688	1418
C_E (2050)	$\$_{2022}$ kW^{-1}	564 [18]	482 [18]	523 [b]	758 [c]	758 [c]	523 [b]
C_E (2025)	$\$_{2022}$ m^{-2}	8119	10502	15876	15912	4287	50572
C_E (2050)	$\$_{2022}$ m^{-2}	3835	3085	5854	4486	1209	18649

Quantity	Unit	Li-NRR (MEA)	Mg-NRR (MEA)	Al-NRR (MEA)
j	A cm^{-2}	0.4	0.4	0.4
E_{cell} [a]	V	3.8	3.1	2.5
$P_{density}$	kW m^{-2}	15	12.5	9.8
C_E (2025)	$\$_{2022}$ kW^{-1}	1418	1418	1418
C_E (2050)	$\$_{2022}$ kW^{-1}	523 [b]	523 [b]	523 [b]
C_E (2025)	$\$_{2022}$ m^{-2}	21270	17725	13896
C_E (2050)	$\$_{2022}$ m^{-2}	7845	6538	5125

[a] E_{cell} for the NRR electrolyzers are calculated at 0.4 A cm^{-2} using the assumptions from Table S15. [b] Average between AEL and PEMEL. [c] Assumed same price as a water SOEL.[20] MEA stands for membrane electrode assembly.

The following set of equations are used to calculate the levelized cost of ammonia (LCOA), which is the NH_3 selling price at which the end-of-life net present value (NPV) is equal to zero:

$$Revenue = LCOA \cdot NH_3\, capacity + H_2\, price \cdot H_2\, capacity + O_2\, price \cdot O_2\, capacity \# (S47)$$

$$Gross\ profit = Revenue - OPEX \# (S48)$$

$$Net\ profit = Gross\ profit - (Gross\ profit - Depreciation) \cdot Tax\ rate \# (S49)$$

$$Depreciation = \frac{Total\ equipment\ cost - Salvage\ value \cdot Total\ equipment\ cost}{Plant\ years} \# (S50)$$

$$Cash\ Flow = Net\ profit + Depreciation\ \# (S51)$$

$$NPV = 0 = \sum_{t=1}^{n} \frac{Cash\ Flow}{(1 + interest\ rate)^t} - total\ capital\ costs \# (S52)$$

In the 0th year (t = 0), the total capital costs are invested into the construction of the plant, while there is no revenue nor operational expenses. It is assumed that the plant is fully operational in year one (t ≥ 1). We used 25% tax rate, 25% salvage value and 4.28% interest rate with a linear depreciation scheme.

Supplementary References

1 Bard, A. J., Faulkner, L. R. & White, H. S. *Electrochemical methods: fundamentals and applications*. (John Wiley & Sons, 2022).

2 Akolkar, R. Mathematical model of the dendritic growth during lithium electrodeposition. *Journal of Power Sources* **232**, 23-28 (2013).

3 Kugler, K., Ohs, B., Scholz, M. & Wessling, M. Towards a carbon independent and CO2-free electrochemical membrane process for NH3 synthesis. *Physical Chemistry Chemical Physics* **16**, 6129-6138 (2014). https://doi.org:10.1039/c4cp00173g

4 Ni, M., Leung, M. K. & Leung, D. Y. Electrochemical modeling of hydrogen production by proton-conducting solid oxide steam electrolyzer. *International Journal of Hydrogen Energy* **33**, 4040-4047 (2008).

5 Liu, Y. *et al.* Insight into the critical role of exchange current density on electrodeposition behavior of lithium metal. *Advanced Science* **8**, 2003301 (2021).

6 Ni, M. Modeling of SOFC running on partially pre-reformed gas mixture. *International Journal of Hydrogen Energy* **37**, 1731-1745 (2012).

7 Fu, X. *et al.* Continuous-flow electrosynthesis of ammonia by nitrogen reduction and hydrogen oxidation. *Science* **379**, 707-712 (2023).

8 Dioxide Materials. *Sustainion Anion Exchange Membranes*, <https://dioxidematerials.com/technology/sustainion-membranes/> (2023).

9 Gilliam, R., Graydon, J., Kirk, D. & Thorpe, S. A review of specific conductivities of potassium hydroxide solutions for various concentrations and temperatures. *International Journal of Hydrogen Energy* **32**, 359-364 (2007).

10 Fernandez, C. A. *et al.* Opportunities for intermediate temperature renewable ammonia electrosynthesis. *Journal of Materials Chemistry A* **8**, 15591-15606 (2020). https://doi.org:10.1039/d0ta03753b

11 Du, H.-L. *et al.* Electroreduction of nitrogen with almost 100% current-to-ammonia efficiency. *Nature 2022 609:7928* **609**, 722-727 (2022). https://doi.org:10.1038/s41586-022-05108-y

12 Towler, G. & Sinnott, R. *Chemical engineering design: principles, practice and economics of plant and process design*. (Butterworth-Heinemann, 2021).

13 Woods, D. R. *Rules of thumb in engineering practice*. (John Wiley & Sons, 2007).

14 Turton, R., Bailie, R. C., Whiting, W. B. & Shaeiwitz, J. A. *Analysis, synthesis and design of chemical processes*. (Pearson Education, 2008).

15 Morgan, E. R. *Techno-economic feasibility study of ammonia plants powered by offshore wind*. (University of Massachusetts Amherst, 2013).

16 Bañares-Alcántara, R. *et al.* Analysis of Islanded Ammonia-based Energy Storage Systems. *University of Oxford*, 1-150 (2015).

17 Mivechian, A. & Pakizeh, M. Hydrogen recovery from Tehran refinery off-gas using pressure swing adsorption, gas absorption and membrane separation technologies: Simulation and economic evaluation. *Korean Journal of Chemical Engineering* **30**, 937-948 (2013).

18 Glenk, G. & Reichelstein, S. Economics of converting renewable power to hydrogen. *Nature Energy* **4**, 216-222 (2019).

19 Böhm, H., Zauner, A., Rosenfeld, D. C. & Tichler, R. Projecting cost development for future large-scale power-to-gas implementations by scaling effects. *Applied Energy* **264**, 114780 (2020).

20 Schmidt, O. *et al.* Future cost and performance of water electrolysis: An expert elicitation study. *International journal of hydrogen energy* **42**, 30470-30492 (2017).

21 International Rewenable Energy Agency (IRENA). Renewable power generation costs in 2022. (2022).

22 International Rewenable Energy Agency (IRENA). Future of Solar Photovoltaic: Deployment, investment, technology, grid intergration and socio-economic aspects (A global Energy Transformation: paper). (2019).
23 Bogdanov, D. *et al.* Radical transformation pathway towards sustainable electricity via evolutionary steps. *Nature communications* **10**, 1-16 (2019).
24 International Rewenable Energy Agency (IRENA). Making The Breakthrough - Green hydrogen policies and technology costs. (2021).
25 INDEXBOX. EU - Oxygen - Market Analysis, Forecast, Size, Trends and Insights. (2022).
26 Hausmann, J. N., Schlögl, R., Menezes, P. W. & Driess, M. Is direct seawater splitting economically meaningful? *Energy & Environmental Science* **14**, 3679-3685 (2021).
27 Administration, U. S. E. I. *Henry Hub Natural Gas Spot Price*, <https://www.eia.gov/dnav/ng/hist/rngwhhdm.htm> (2023).
28 Bashmakov, I. A. *et al.* IPCC. Climate Change 2022: Mitigation of Climate Change. Industry. (2022).
29 Smith, R. *Chemical process: design and integration*. (John Wiley & Sons, 2005).
30 Appl, M. *Ammonia: Principles and Industrial Practice*. (1999).
31 Van Der Ham, C. J. M., Koper, M. T. M. & Hetterscheid, D. G. H. Challenges in reduction of dinitrogen by proton and electron transfer. *Chemical Society Reviews* **43**, 5183-5191 (2014). https://doi.org:10.1039/c4cs00085d
32 Skúlason, E. *et al.* A theoretical evaluation of possible transition metal electro-catalysts for N2 reduction. *Physical Chemistry Chemical Physics* **14**, 1235-1245 (2012). https://doi.org:10.1039/c1cp22271f
33 Noren, D. & Hoffman, M. A. Clarifying the Butler–Volmer equation and related approximations for calculating activation losses in solid oxide fuel cell models. *Journal of Power Sources* **152**, 175-181 (2005).
34 Buttler, A. & Spliethoff, H. Current status of water electrolysis for energy storage, grid balancing and sector coupling via power-to-gas and power-to-liquids: A review. *Renewable and Sustainable Energy Reviews* **82**, 2440-2454 (2018). https://doi.org:10.1016/j.rser.2017.09.003
35 Nel Hydrogen. *Nel Hydrogen Electrolyzers - The World's Most Efficient and Reliabel Electrolyzers*, <https://nelhydrogen.com/wp-content/uploads/2020/03/Electrolysers-Brochure-Rev-D.pdf> (2020).
36 Siemens Energy. *Overview of the PEM Silyzer Family*, <https://4echile.cl/wp-content/uploads/2020/10/20200930-SE-NEB-PEM-Electrolyzer-and-Applications_EW.pdf> (2020).
37 Sánchez, A. & Martín, M. Scale up and scale down issues of renewable ammonia plants: Towards modular design. *Sustainable Production and Consumption* **16**, 176-192 (2018). https://doi.org:10.1016/j.spc.2018.08.001
38 Bocker, N. & Grahl, M. Nitrogen - Air separation technology. *Ullmann's Encyclopedia of Industrial chemistry* (2013).
39 Marsidi, M. *Technology Factsheet - Electric Industrial Boiler*, <https://energy.nl/wp-content/uploads/electric-industrial-boiler-7.pdf> (2018).
40 Marc Marsidi, Luuk Beurskens & Uslu, A. The role of renewable heat technologies in industry - a review of Dutch sectoral industry roadmaps. (2018).
41 Lin, B., Wiesner, T. & Malmali, M. Performance of a Small-Scale Haber Process: A Techno-Economic Analysis. *ACS Sustainable Chemistry & Engineering* **8**, 15517-15531 (2020). https://doi.org:10.1021/ACSSUSCHEMENG.0C04313
42 Helminen, J., Helenius, J., Paatero, E. & Turunen, I. Comparison of sorbents and isotherm models for NH3-gas separation by adsorption. *AIChE journal* **46**, 1541-1555 (2000).

43 Park, Y., Ju, Y., Park, D. & Lee, C.-H. Adsorption equilibria and kinetics of six pure gases on pelletized zeolite 13X up to 1.0 MPa: CO2, CO, N2, CH4, Ar and H2. *Chemical Engineering Journal* **292**, 348-365 (2016).

44 Knaebel, K. S. A “How to” guide for adsorber design. *Adsorption Research, Inc. Dublin, Ohio* **43016** (2007).

45 Ntiamoah, A., Ling, J., Xiao, P., Webley, P. A. & Zhai, Y. CO2 capture by temperature swing adsorption: use of hot CO2-rich gas for regeneration. *Industrial & Engineering Chemistry Research* **55**, 703-713 (2016).

46 Scuiller, E., Dutournié, P., Zbair, M. & Bennici, S. New Approach for Measuring the Specific Heat Capacity of Reactive Adsorbents Using Calorimetry. *Journal of Chemical & Engineering Data* **68**, 1865-1871 (2023).

47 Spiller, L. L. Determination of ammonia/air diffusion coefficient using nafion lined tube. *Analytical letters* **22**, 2561-2573 (1989).

48 Winn, E. B. The temperature dependence of the self-diffusion coefficients of argon, neon, nitrogen, oxygen, carbon dioxide, and methane. *Physical review* **80**, 1024 (1950).

49 The Engineering Toolbox. *Air - Diffusion Coefficients of Gases in Excess of Air*, <https://www.engineeringtoolbox.com/air-diffusion-coefficient-gas-mixture-temperature-d_2010.htm> (2018).

50 Salomone, F. *et al.* Process simulation and energy analysis of synthetic natural gas production from water electrolysis and CO2 capture in a waste incinerator. *Applied Energy* **343**, 121200 (2023).

51 Bannwarth, H. *Liquid ring vacuum pumps, compressors and systems: conventional and hermetic design*. (John Wiley & Sons, 2006).

52 Coker, A. K. & Ludwig, E. E. Ludwig's applied process design for chemical and petrochemical plants. *Applied process design for chemical and petrochemical plants* (2007).

53 Ramdin, M. *et al.* High-pressure electrochemical reduction of CO2 to formic acid/formate: effect of pH on the downstream separation process and economics. *Industrial & Engineering Chemistry Research* **58**, 22718-22740 (2019).

54 Ramdin, M. *et al.* Electroreduction of CO2/CO to C2 products: process modeling, downstream separation, system integration, and economic analysis. *Industrial & Engineering Chemistry Research* **60**, 17862-17880 (2021).

55 Spurgeon, J. M. & Kumar, B. A comparative technoeconomic analysis of pathways for commercial electrochemical CO 2 reduction to liquid products. *Energy & Environmental Science* **11**, 1536-1551 (2018).

56 International Rewenable Energy Agency (IRENA). Green Hydrogen Cost Reduction - Scaling up electrolysers to meet the 1.5 degree C climate goal. (2020).
